# Molecular dynamics simulations for understanding dual ubiquitination mechanisms to consider S-phase kinase-associated protein 2 (SKP2) as a potential drug target in breast cancer

**DOI:** 10.3389/fchem.2026.1786015

**Published:** 2026-05-29

**Authors:** Manshi Kumari Gupta, C. Sudandiradoss

**Affiliations:** Department of Biotechnology, School of Bio Sciences and Technology, Vellore Institute of Technology, Vellore, Tamilnadu, India

**Keywords:** breast cancer, dual ubiquitination, isopeptide bond, K48 linkage and K63 linkage, proteasomal degradation and activation, Skp2

## Abstract

**Introduction:**

Dual ubiquitination mediated by S-phase kinase-associated protein 2 (SKP2) plays a critical role in breast cancer progression by directing substrates toward either proteasomal degradation or signaling activation. However, the structural determinants governing SKP2 recognition of different ubiquitin-linked substrates, particularly p27 and Akt1, remain poorly understood.

**Methods:**

To investigate this mechanism, integrative computational approaches including protein–protein docking, molecular dynamics simulations, hydrogen-bond analysis, radius of gyration (Rg), root mean square deviation (RMSD), and MM/PBSA free-energy calculations were employed. Isopeptide bonds were constructed between the C-terminal Gly76 of ubiquitin and Lys134 of p27 (K48-linked) or Lys135 of Akt1 (K63-linked) to represent the post-ubiquitin transfer stage and preserve native ubiquitin conjugation geometry.

**Results:**

Computational analyses revealed distinct substrate recognition patterns by SKP2. The p27 complex adopted a compact interaction interface favorable for selective recognition, whereas Akt1 displayed a broader interaction surface supporting stable engagement. Docking studies demonstrated strong SKP2 interactions with UbK48-p27 and UbK63-Akt1 complexes, yielding HADDOCK scores of −103.8 and −85.5, respectively. Molecular dynamics simulations showed that UbK48 induced structural loosening in the SKP2-p27 complex (RMSD 1.61 nm; Rg 3.57 nm), consistent with proteasomal targeting, while UbK63 maintained a compact and stable SKP2-Akt1 assembly (RMSD 0.86 nm; Rg 3.33 nm), supporting sustained signaling activity. Hydrogen-bond and MM/PBSA analyses further demonstrated destabilization of SKP2-p27 (10.96 H-bonds; −61.63 kcal/mol) and stabilization of SKP2-Akt1 (14.42 H-bonds; −88.69 kcal/mol).

**Discussion:**

This study provides the first atomistic evidence of isopeptide-mediated dual ubiquitination by SKP2. The findings identify distinct structural determinants that govern substrate-specific outcomes toward degradation or activation, revealing potential regulatory interfaces within SKP2–substrate complexes. These insights establish a framework for developing computationally prioritized strategies to design targeted inhibitors aimed at disrupting oncogenic signaling and overcoming SKP2-driven drug resistance in breast cancer.

## 1 Introduction

Protein degradation forms a central component of overall protein turnover, allowing cells to rapidly reclaim amino acids and adjust to changing extracellular conditions. Among the systems that regulate this process, the ubiquitin proteasome system (UPS) operates as the primary post-translational pathway responsible for directing targeted protein breakdown (Wilkinson, 1987). By efficiently clearing misfolded or damaged proteins, the UPS maintains intracellular protein balance and supports essential processes such as apoptosis (Vucic et al., 2011) and controlled cell-cycle progression (Nakayama and Nakayama, 2006). Beyond these degradative roles, it is now well established that ubiquitin signaling also mediates several non-proteolytic functions, including metabolic regulation (Lee et al., 2019), DNA repair (Kim et al., 2021), autophagy (Shi and Kehrl, 2010), signal pathway modulation (Yang et al., 2009), and immune coordination (Dybas et al., 2019). Consequently, when UPS function is disrupted, these regulatory failures can drive diverse pathologies, most notably cancer (Senft et al., 2018), Parkinson’s disease (Wade Harper et al., 2018), and Alzheimer’s disease (Zhang et al., 2017).

Building on this central role of the UPS, it is important to consider the molecular basis of ubiquitin signaling itself. Ubiquitin, a highly conserved 76 amino-acid modifier, is attached to cellular substrates through isopeptide bonds formed between its C-terminal glycine and lysine residues located on the target protein or on ubiquitins within an existing chain (Pan et al., 2022). Because ubiquitin contains seven lysines and one methionine capable of receiving additional ubiquitin molecules, it can generate a broad repertoire of chain architectures with unique regulatory implications (Akutsu et al., 2016; Li and Ye, 2008). These architectures ultimately shape the functional outcome of the modification: UbK48 chains direct substrates to the proteasome for degradation (Mallette and Richard, 2012), while UbK63 chains support non-proteolytic roles, such as enabling protein activation, guiding localization, and stabilizing signaling platforms (Okamoto et al., 2018). Moreover, in addition to these canonical linkages, atypical chain types have recently been identified, further expanding the regulatory repertoire of ubiquitination (Chan et al., 2012).

Given the diversity of ubiquitin chain types, attention naturally turns to the enzymatic machinery that builds them. This complex system involves three enzyme types: E1 activators, E2 carriers - alongside E3 ligases. Of these, E3s determine target selection; here, the SCF group (Skp1/Cul-1/Rbx-1/SKP2) serves as a clear case (Bulatov and Ciulli, 2015). Within this complex, S-phase kinase-associated protein 2 (SKP2) functions as a critical substrate recognition factor, guiding proteins toward proteasomal degradation (Nakayama and Nakayama, 2006). Notably, SKP2 has attracted considerable attention due to its prominent role in tumorigenesis (Wei et al., 2012). This critical is consistently upregulated in a broad spectrum of malignancies, including prostate, gastric, lung, and breast cancers, along with lymphomas and melanoma (Arbini et al., 2011; Chan et al., 2012; Wang et al., 2014; Wei et al., 2012; William et al., 2024). Within breast cancer cohorts, elevated SKP2 expression aligns with heightened tumor aggressiveness and is indicative of adverse clinical outcomes. Importantly, SKP2 exerts a dual ubiquitination role in breast cancer by targeting both tumor suppressors and oncogenic proteins through distinct ubiquitin linkages (Davidovich et al., 2008; Ravaioli et al., 2008). This dual role highlights how SKP2 works in two ways - on one hand driving protein breakdown through UbK48-linked chains targeting p27, on the other triggering cell signals like Akt1 activation using UbK63-linked chains.

To show this dual role more clearly, a well-studied target of SKP2 is p27^Kip1 (p27), which blocks cyclin-dependent kinases and acts as a tumor suppressor in breast cancer. Instead of working alone, SKP2 teams up with the helper protein Cks1 to grab hold of p27 when it is been marked by phosphate groups. Because of this interaction, abnormal levels of SKP2 lead to faster breakdown of p27, pushing cells into S-phase sooner (Fujita et al., 2008). Phosphorylation of p27 at Thr187 is required for its recognition and ubiquitination by the nuclear SCF–SKP2 complex, a process that ultimately targets p27 for proteasomal degradation. Consistently, diminished p27 expression represents a recurrent molecular alteration in breast tumors and reliably correlates with enhanced malignancy and unfavorable patient prognosis (Fujita et al., 2008; Ravaioli et al., 2008).

In addition to regulating p27 turnover, SKP2 extends its activity to Akt1, a serine/threonine kinase that integrates PI3K-derived cues to control cell survival, metabolic flux and protein synthesis. Aberrant Akt signaling, in turn, underlies a wide spectrum of pathological states, spanning metabolic and autoimmune disorders to cardiovascular, neurological and oncogenic diseases (Chan et al., 2012). Although Akt1 activation has been predominantly attributed to PI3K-mediated phosphorylation, emerging evidence demonstrates that its regulation also depends on non-proteolytic ubiquitination. Specifically, SCF–SKP2 catalyzes UbK63-linked ubiquitin modification of Akt1, a modification that promotes Akt1 phosphorylation and membrane localization, thereby potentiating pathway activation (Meng et al., 2025; Yang et al., 2009). Also, SKP2 has long been viewed mainly as a factor that directs its targets toward degradation, recent findings show that this is not the full story. Akt1 emerges as a clear exception, functioning as a SKP2 substrate that is regulated through UbK63-linked ubiquitin rather than being routed to the proteasome (Feng et al., 2024). This adds an additional dimension to SKP2 biology: the same ligase can impose two very different outcomes depending on the substrate involved.

Taken together, these findings show SKP2 can attach two different ubiquitin chains: one kind marks the tumor suppressor p27 for breakdown using UbK48 links; at the same time, another type switches on the cancer-linked enzyme Akt1 by means of UbK63 linkages. Previous computational studies have laid a foundation for understanding these interactions, primarily through static docking and virtual screening aimed at disrupting the SKP2-Cks1-p27 interface. Research utilizing molecular dynamics (MD) has also characterized the intrinsic flexibility of the SKP2 F-box domain, suggesting that conformational plasticity is essential for substrate recruitment. Furthermore, structural bioinformatics has been employed to map the surface topography of the Leucine-Rich Repeat (LRR) domain to identify potential small-molecule binding pockets. However, these prior models typically treat the SKP2-substrate complex as a non-covalent recognition event, often overlooking the atomistic transitions and geometric constraints imposed by the actual isopeptide bond linkage. Specifically, the structural divergence between K48-linked degradation and K63-linked activation remains poorly defined in existing computational literature (Chandra et al., 2016; Wu et al., 2012). In breast tumors, these two processes occur simultaneously: Akt1 signaling becomes hyperactivated, while the progressive loss of p27 weakens a critical barrier that normally restrains cell proliferation. Although substantial experimental evidence supports this dual functional imbalance, the molecular basis underlying SKP2-mediated selectivity toward these substrates remains unclear. In particular, it is not yet fully understood how SKP2 distinguishes between different targets and determines the specific ubiquitin chain linkage to assemble. Therefore, elucidating the structural determinants of this selectivity remains an important unresolved question.

This unresolved structural question directly motivates the present study. Addressing this gap requires structural insight into how SKP2 engages with p27 for UbK48-linked degradation and with Akt1 for UbK63-linked activation. From a therapeutic perspective, inhibiting SKP2-mediated UbK48 ubiquitination of p27 could restore tumor suppressor function, while selectively blocking UbK63 ubiquitination of Akt1 may attenuate oncogenic signaling without broadly impairing SKP2’s other roles. Through integrative computational structural biology combining high-resolution protein-protein docking, atomistic molecular dynamics simulations, and quantitative interface energetics analyses, we will delineate the conformational landscapes and binding determinants that govern SKP2 interactions with p27 and Akt1. By doing so, we can mechanistically distinguish the structural cues that bias UbK48 *versus* UbK63 chain assembly, thereby providing predictive insight into SKP2’s dual ubiquitination role and enabling a rational framework for therapeutic intervention in breast cancer.

## 2 Materials and methods

### 2.1 SKP2-from sequence to structure

The amino acid sequences of human S-phase kinase-associated protein 2 (SKP2, UniProt ID: Q13309), cyclin-dependent kinase inhibitor 1B (p27, UniProt ID: P46527), K48-linked polyubiquitin (UbK48), RAC-alpha serine/threonine-protein kinase (Akt1, UniProt ID: P31749), and K63-linked polyubiquitin (UbK63) were obtained from the UniProt Knowledgebase (Apweiler et al., 2004), a curated repository that provides validated protein sequences together with functional annotations. Because accurate sequences are the foundation for reliable structural modeling, we next generated three-dimensional models using a combination of comparative modeling and artificial intelligence based prediction. This approach was adopted to ensure structural completeness and to obtain conformationally unbiased starting models, as several available experimental structures do not provide complete residue coverage and may represent SKP2 in complex-bound conformations. Therefore, AI-based modeling enabled us to generate complete and conformationally consistent structures suitable for downstream docking and molecular dynamics simulations. To begin, SKP2 was reconstructed through template-guided modeling, with template A2HY identified via the SAS utility in PDBsum (Laskowski, 2022). This template provided complete sequence identity and therefore ensured that the reconstructed SKP2 structure was faithful to the native protein. Building on this, p27 was modeled using homology-based reconstruction with the AlphaFold DB entry P46527.1. A as the primary structural template. Its reliability was subsequently evaluated using GMQE and QMEAN scoring metrics, which confirmed the accuracy of the generated model for subsequent docking and molecular dynamics simulations (Waterhouse et al., 2018). To ensure that the structural reconstruction of p27 accurately reflected its biological nature, an intrinsic disorder prediction analysis was performed using the IUPred2A web server (Mészáros et al., 2018). This step was essential to reconcile the atomistic model with the known classification of p27 as an intrinsically disordered protein (IDP) and to benchmark the conformational flexibility of the coil-heavy regions identified via the SAS utility in PDBsum. In addition, UbK48 was examined using a dual strategy. On one hand, we employed conventional homology-driven reconstruction; on the other, we applied NeuroSNAP (Roberts et al., 2024), a deep learning framework trained on large-scale structural datasets. This combined approach allowed us to capture conformations beyond canonical restraints, thereby reflecting the dynamic flexibility of polyubiquitin chains. Following this, Akt1 was reconstructed with emphasis on its kinase domain, which plays a central role in phosphorylation-mediated signaling. Template 6HHJ, identified through PDBsum alignment, guided this reconstruction and ensured accurate domain representation. By contrast, UbK63 was not modeled computationally but directly retrieved as an experimentally resolved crystal structure (PDB ID: 5GOJ) (Vallat et al., 2025). This choice guaranteed maximal accuracy by relying on crystallographic data rather than predictive modeling. Finally, to complement all reconstructed and retrieved structures, we extracted secondary structural features including α-helices, β-strands, domains, and topology diagrams from PDBsum, which provides schematic representations of protein architecture.

### 2.2 Structural quality assessment (SQA)

The reliability of the predicted protein structures was examined through a combination of established computational validation approaches. ERRAT (Colovos and Yeates, 1993) provided an evaluation of non-bonded atomic interactions, highlighting regions that deviate from patterns observed in high-resolution crystallographic data. Backbone stereochemistry was further analyzed with PROCHECK, which generated Ramachandran plots to map the distribution of residues across favored, allowed, and outlier regions of the φ–ψ dihedral angle space (Laskowski et al., 1993).

To complement these checks, MolProbity (Chen et al., 2010) offered an all-atom assessment of steric clashes, rotamer conformations, and backbone geometry, yielding refinement indicators such as clash score and overall MolProbity score. Global and local accuracy of the models was estimated with ProQ (Wallner and Elofsson, 2003), which applies machine-learning–based scoring functions and reports values including LGscore and MaxSub to reflect fold correctness. Finally, ProSA (Sippl, 1993) was then employed to calculate Z-scores, benchmarking each predicted model against experimentally solved structures and thereby providing an overall measure of structural plausibility. In combination with the other validation platforms, these results delivered a multidimensional assessment of stereochemical integrity, atomic packing, and fold stability. Collectively, the outcomes confirmed that the predicted models possess sufficient reliability to serve as the basis for subsequent structural and functional investigations.

### 2.3 Active sites prediction and molecular docking of SKP2-p27 and SKP2-Akt1 without ubiquitin linkage

Potential binding interfaces were identified using PrankWeb (Jendele et al., 2019), which integrates structural descriptors with evolutionary conservation scoring to highlight residues most likely to participate in intermolecular contacts. The modeled structures of SKP2, p27, and Akt1 were uploaded in PDB format, and the highest-ranked pockets were extracted. Importantly, residues that overlapped with conserved hotspots were designated as active interface residues, ensuring biologically relevant docking inputs.

Docking simulations were then performed with HADDOCK 2.4 (Van Zundert et al., 2016), an integrative platform that incorporates user-defined interface residues into its workflow. The protocol proceeds through three sequential stages: rigid-body energy minimization to generate initial complexes, semi-flexible simulated annealing to refine sidechain and backbone conformations, and explicit-solvent refinement to optimize the final structures under aqueous conditions. Also, HADDOCK does not rely on a fixed grid box like conventional docking tools; instead, it uses an information-driven approach guided by user-defined active and passive residues Initially, SKP2 was docked with p27 to characterize substrate-recognition orientation and interface complementarity. Subsequently, SKP2 was docked with Akt1 using the same guided procedure, with active residues defined by PrankWeb predictions and conserved interaction patches.

To independently validate the HADDOCK-derived binding modes and address potential biases in the predefined docking constraints, the complexes were re-modeled using AlphaFold 3 (AF3) (Abramson et al., 2024). Sequences for the respective proteins were submitted to the AlphaFold for multimeric structure prediction without the use of structural templates or distance constraints. The top-ranked AF3 models were subsequently superimposed onto the corresponding HADDOCK docking poses using the PyMOL Molecular Graphics System. Structural convergence was quantified by calculating the all-atom Root Mean Square Deviation (RMSD) for the entire substrate-bound complex. Furthermore, to evaluate the molecular recognition differences at the atomic level, a comparative superimposition was performed by aligning the conserved SKP2 backbone (Chain A) using the “*super”* command. This dual-validation approach ensured that the predicted binding interfaces were both structurally robust and consistent across different computational methodologies.

The resulting complexes were ranked according to HADDOCK scores and Z-scores, and the single most favorable cluster for each complex was selected as the representative model for downstream analysis.

To further characterize the binding energetics and identify the precise “atomic hotspots” within the validated interfaces, we performed a quantitative analysis using the PDBePISA (Proteins, Interfaces, Structures and Assemblies) tool (Battle, 2016). This analysis allowed for the determination of the interfacial buried surface area (BSA), the calculation of the solvation free energy gain (Delta G), and the systematic mapping of the hydrogen bond and salt bridge networks. This dual-validation and interface characterization approach ensured that the predicted binding orientations specifically the divergent C-terminal (p27) and N-terminal/middle (Akt1) footprints were both structurally robust and biologically relevant.

Finally, structural evaluation, interaction mapping, and visualization of the docked conformations were carried out in PyMOL (Rigsby and Parker, 2016), providing atomic-level insights into binding orientations and interface stability.

### 2.4 Modeling of post-transfer ubiquitin conjugates and molecular docking of SKP2–p27 and SKP2–Akt1 complexes

To represent biologically relevant post-transfer (ubiquitin) states, *in silico* conjugates were generated corresponding to K48-linked degradation and K63-linked signaling modifications. Specifically, an isopeptide bond was introduced between the C-terminal Gly76 of ubiquitin and the ε-amino group of the target lysine on each substrate. By constructing this covalent linkage, the computational models enable structural interrogation of ubiquitin-modified substrates in complex with SKP2 (Faggiano et al., 2016). The enzymatic transfer of ubiquitin from the E2 enzyme to the substrate was not explicitly modeled, as such processes require quantum mechanical/molecular mechanical (QM/MM) approaches; consequently, our analysis focused on the structural dynamics of the resulting post-transfer complexes.

In this study, we specifically utilized a monoubiquitin scaffold to characterize the initial structural determinants of linkage specificity. This approach is grounded in the “Nucleation-Elongation” model, which identifies the formation of the first isopeptide bond as the primary regulatory bottleneck for E3-substrate recognition (Pickart and Eddins, 2004). By simulating the 1:1 substrate-ubiquitin complex, we reduced the conformational entropy and structural noise associated with longer, disordered polyubiquitin chains (Faggiano et al., 2016). This allowed for a high-resolution analysis of the high-affinity salt bridges and hydrogen bonding networks that distinguish proteolytic from non-proteolytic signaling at the point of commitment (Komander and Rape, 2012).

Prior to isopeptide bond construction, ubiquitin and substrate structures were prepared in PyMOL (Rigsby and Parker, 2016) and UCSF Chimera (Pettersen et al., 2004). Terminal atoms were removed, side chains were cleaned, and Gly76 together with the target lysine was oriented in a geometry suitable for isopeptide linkage construction. Subsequently, the Gly76-Lys isopeptide bond was manually constructed using covalent-bond editing tools, and local distortions were corrected through loop and side-chain refinement in MODELLER (Eswar et al., 2003). Following refinement, geometry optimization and force-field parameterization of the modified environment were performed using AMBER/CHARMM (Lee et al., 2020) preparation utilities. In addition, a local energy minimization step was carried out after bond construction to relieve steric strain and unfavorable contacts introduced during covalent modeling, thereby ensuring a physically stable structure prior to docking. To further confirm chemical validity, the edited linkage was examined using Avogadro (Hanwell et al., 2012), verifying appropriate atom types and valence states.

Once validated, the Ub-p27 (K48-linked mimic) and Ub-Akt1 (K63-linked mimic) conjugates were treated as single covalently linked entities for docking. The refined structures were submitted to HADDOCK 2.4 (Van Zundert et al., 2016), with SKP2 defined as the receptor. Active and passive residues for both partners were specified based on PrankWeb predictions and known SKP2 recognition motifs. Finally, docking followed the standard three-stage HADDOCK protocol comprising rigid-body docking, semi-flexible refinement, and explicit solvent refinement yielding the final complexes for SKP2-UbK48-p27 and SKP2-UbK63-Akt1.

### 2.5 Post-docking interface and binding affinity analysis of SKP2–Substrate complexes

To comprehensively evaluate SKP2’s substrate recognition in the context of dual ubiquitination, post-docking analyses were performed for four complexes: SKP2-p27, SKP2-UbK48-p27, SKP2-Akt1, and SKP2-UbK63-Akt1. Structural interface characterization was conducted using the PDBsum server (Laskowski et al., 2018), which generated detailed maps of intermolecular contacts, including hydrogen bonds, salt bridges, hydrophobic interactions, and closest atomic distances. These contact profiles enabled comparative visualization of how ubiquitin linkage type and substrate identity influence SKP2’s binding orientation and interface architecture. To further resolve residue-level contact networks, LigPlot+ (Laskowski and Swindells, 2011) was employed. Although primarily designed for protein-ligand analysis, its DIMPLOT module effectively captured non-covalent interactions within protein–protein interfaces. These schematic diagrams revealed conformational adjustments at the SKP2 binding cleft, particularly those induced by the steric and electrostatic presence of covalently attached ubiquitin chains in the UbK48-p27 and UbK63-Akt1 conjugates.

Binding affinity estimation was performed using the PRODIGY server (Vangone and Bonvin, 2017). To ensure structural consistency with the docking results, the top-ranked representative conformation from the best HADDOCK cluster selected based on the lowest HADDOCK score and the most significant Z-score was used for each of the four complexes. This platform calculates interaction free energies (ΔG) and predicts dissociation constants (Kd) at 25 °C by correlating interfacial contact density with experimental binding affinities (Kastritis and Bonvin, 2010; Kastritis et al., 2011; Vreven et al., 2015). In this way, the post-docking evaluation provided a multidimensional characterization of SKP2’s substrate engagement, integrating geometric, chemical, and thermodynamic parameters to clarify its mechanistic role in dual ubiquitination.

### 2.6 Structural stability and dynamic behavior analysis (RMSD, RMSF, and Rg)

To evaluate the stability and dynamic properties of the docked protein complexes, molecular dynamics (MD) simulations were carried out with the GROMACS 4.5 package (Abraham et al., 2015; Pronk et al., 2013). The OPLS force field was applied to ensure accurate treatment of both bonded and non-bonded interactions. To maintain physiological relevance, the protonation states of all titratable residues were assigned corresponding to neutral pH (pH 7.0), following standard force field conventions. This ensures appropriate representation of electrostatic interactions and hydrogen bonding networks during the simulation. Subsequently, each complex was solvated in a cubic simulation box containing SPC216 water molecules, with a minimum distance of 1.0 nm maintained between the protein surface and the box boundary to prevent artefactual self-contacts. Finally, to ensure overall system neutrality, an appropriate number of sodium counter-ions were introduced.

Prior to production runs, all systems underwent energy minimization using the steepest descent algorithm (convergence threshold: 1000 kJ/mol/nm; maximum 50,000 steps) to eliminate steric clashes. Equilibration was carried out in two stages: an initial 50 ps under the NVT ensemble with Particle Mesh Ewald (PME) electrostatics, followed by NPT conditions to stabilize system pressure.

The production run extended for 200 ns at 300 K, with snapshots recorded every 100 ps. To ensure reproducibility, simulations were performed in triplicate. Analyses focused on structural descriptors: Root Mean Square Deviation (RMSD) and Root Mean Square Fluctuation (RMSF) were calculated using the protein backbone atoms (N-Cα-C) to evaluate global stability and residue-level flexibility, respectively. For the individual SKP2 framework, the RMSD was computed to establish a structural baseline, whereas for the multi-protein assemblies, the RMSD was calculated for the entirety of the substrate-bound and ubiquitin-linked complexes. In addition, potential energy profiles were monitored to confirm thermodynamic stability throughout the 200 ns window. Pre- and post-MD structures were aligned to detect conformational adjustments induced during equilibration.

To further validate the docking results, time-resolved intermolecular interaction analysis was conducted. Representative snapshots were extracted at 50, 100, 150, and 200 ns and processed via DIMPLOT (within the LIGPLOT + suite) (Laskowski and Swindells, 2011) to track the evolution of hydrogen bonds and hydrophobic contacts throughout the trajectory. Visualization and trajectory post-processing were conducted with VMD (Humphrey et al., 1996), plots were generated using Xmgrace, and final structural figures were prepared in PyMOL (Rigsby and Parker, 2016).

### 2.7 Lee-Richards algorithm-based SASA and hydrogen bond mechanisms underpinning complex stability

To examine the role of solvent interactions and hydrogen bonding in stabilizing SKP2–substrate complexes, we analyzed solvent-accessible surface area (SASA) (Durham et al., 2009) and hydrogen bond (H-bond) (Berendsen et al., 1995) profiles from the 200 ns MD trajectories. SASA was calculated using the gmx sasa utility, which implements the Lee-Richard’s algorithm to quantify the exposure of amino acid residues to solvent molecules. By monitoring SASA throughout the simulations, we identified changes in surface accessibility, hydrophobic burial, and solvent shielding associated with complex formation.

In parallel, H-bond analysis was carried out with the gmx hbond tool, applying geometric criteria that define a bond when the donor–acceptor distance is ≤0.35 nm and the donor–hydrogen–acceptor angle is ≥135°. This approach enabled quantification of bond number, occupancy, and persistence, distinguishing transient contacts from those that remained conserved throughout the trajectory. Particular emphasis was placed on intermolecular H-bonds at the protein-protein interface, since their frequency and durability directly reflect the strength and specificity of SKP2 recognition. By combining SASA and H-bond data, we developed an integrated view of how solvent exposure, interface rearrangements, and hydrogen-bonding networks contribute to complex stability during the 200 ns simulations. Time-dependent plots were generated in Xmgrace (Turner, 2005), and representative structural snapshots highlighting key changes were examined in VMD (Humphrey et al., 1996) and rendered in PyMOL (Rigsby and Parker, 2016). This approach ensured that quantitative trends were consistently supported through visual inspection.

### 2.8 Conformational landscape and collective motion analysis (FEL and PCA)

The collective motions and conformational preferences of SKP2–substrate complexes were explored through principal component analysis (PCA) in combination with free energy landscape (FEL) mapping. For PCA, the 200 ns MD trajectories were first aligned to their reference structures to eliminate global translational and rotational displacements, ensuring that only internal fluctuations were retained for analysis. Backbone atom deviations from their time-averaged coordinates were used to construct a covariance matrix which describes correlated motions between atomic pairs. Subsequent diagonalization of this matrix produced eigenvalues and eigenvectors, with the largest eigenvalues indicating the dominant modes of motion. By projecting the trajectories onto the leading eigenvectors, it was possible to visualize how each complex sampled conformational space across the simulation.

On the basis of these principal components, the FEL was generated to place the observed dynamics within an energetic framework. The first two components were selected as reaction coordinates, as they represent the most biologically meaningful collective motions. The distribution of trajectory frames along these coordinates was converted into free energy values using the Boltzmann relation, thereby linking conformational sampling to thermodynamic stability. Instead of describing the system in terms of isolated snapshots, this approach revealed a continuous energy surface in which densely populated regions corresponded to stable conformations, while sparsely visited areas reflected higher-energy states. The resulting two-dimensional landscape illustrated both the depth and spread of energy minima, providing a clear picture of how the complexes navigated between alternative conformations. By combining PCA with FEL analysis, we were able to link the dominant structural fluctuations directly to their energetic consequences. PCA clarified which collective motions governed the dynamics of the complexes, while FEL placed those motions into a thermodynamic framework that revealed the conformations most consistently favored during the simulation. This analysis provided a dual perspective that revealed the pathways through which the complexes transitioned between alternative states and underscored the stability of conformations that were repeatedly sampled. Computations were performed with GROMACS (Abraham et al., 2015), and the resulting energy surfaces were visualized as contour plots in Xmgrace (Turner). Graphical inspection complemented the numerical results, reinforcing the consistency of the observed trends.

### 2.9 MM/PBSA binding free energy calculations

Binding free energy was assessed for each simulated complex through the MM/PBSA method available in the gmx_MMPBSA pipeline (Genheden and Ryde, 2015). Snapshots were taken from the equilibrated segment of the 200 ns trajectories so that the calculations represented the stable conformational ensemble rather than transient fluctuations. This ensured that the reported values reflected the energetics of well-relaxed structures, providing a reliable basis for subsequent comparison across complexes.

For every snapshot, the free energies of the complex, receptor, and ligand were computed separately, and the binding energy was obtained by combining the molecular mechanics and solvation terms. The nonpolar contribution was estimated from solvent-accessible surface area values, representing the energetic cost of burying hydrophobic regions. Combined with the electrostatic component, these values defined the overall solvation energy, which reflects how solvent interactions influence complex stability.

The total binding free energy (ΔTOTAL) shown in the graphical output was then calculated by adding the gas-phase energy to the solvation term.

ΔG_gas=ΔVDWAALS+ΔEEL

Solvation effects were separated into polar and nonpolar components. The contribution of solvation was separated into polar and nonpolar components to capture the distinct effects of the solvent environment. The polar term was calculated using the Generalized Born/Poisson–Boltzmann model, which accounts for electrostatic interactions between the solute and surrounding solvent. In contrast, the nonpolar term was estimated from solvent-accessible surface area values, reflecting the energetic cost of burying hydrophobic regions. When combined, these two components yielded the overall solvation energy, providing a comprehensive measure of how the solvent influenced the stability of the complex.

ΔGsolv=ΔEGB+ΔESURF

ΔGbind=ΔGgas+ΔGsolv

Energy values from the selected MD snapshots were averaged so that each complex was represented by a stable mean rather than by individual fluctuations. Using this approach ensured that the comparison of binding strengths was based on consistent numerical estimates. The averaged results then revealed how different energetic contributions-van der Waals interactions, electrostatics, and solvation worked together to define the overall binding profile of the complexes.

## 3 Results

### 3.1 Three-dimensional structural modeling and topological characterization of SKP2, p27, UbK48, Akt1, and UbK63 proteins

**FIGURE 1:**
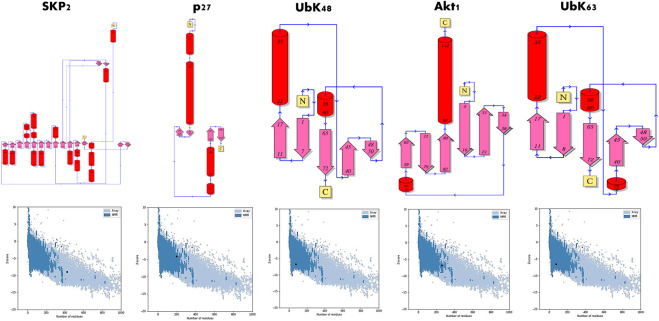
Secondary structure elements and overall quality assessment of human proteins: S-phase kinase-associated protein 2 (SKP2), UbK63-linked ubiquitin, RAC-alpha serine/threonine-protein kinase (AKT1), cyclin-dependent kinase inhibitor 1B (p27), and UbK48-linked ubiquitin. In the topology diagrams, red cylinders represent α-helices, pink arrows indicate β-strands forming β-sheets, and small blue arrows show the direction of the polypeptide chain from N- to C-terminus. The blue plots below each 3D structure depict ProSA Z-score profiles, reflecting model quality relative to experimentally determined X-ray and NMR structures.

**FIGURE 2:**
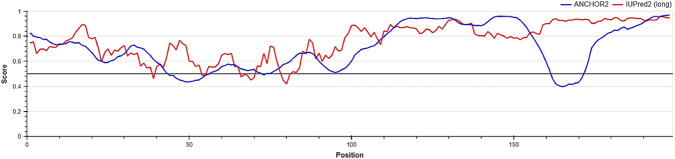
Intrinsic disorder and binding propensity profile of p27. IUPred2 scores (red) remain above the 0.5 threshold, confirming p27 as an intrinsically disordered protein. ANCHOR2 peaks (blue) at residues 110–150 and 175–198 identify functional motifs that undergo a disorder-to-order transition upon binding the SKP2 complex.

The three-dimensional (3D) structures and corresponding two-dimensional (2D) topology diagrams of SKP2, p27, UbK48, Akt1, and UbK63 were successfully obtained and analyzed using PDBsum (Figure 1). SKP2 was modeled using the high-confidence A2HY template, yielding a framework where 127 residues (30%) are organized into alpha-helices and 110 residues (26%) into beta-strands, with the remaining 187 residues (44%) distributed across flexible coil regions, consistent with its leucine-rich repeat (LRR) architecture. A high GMQE (100% sequence identity) score confirmed the reliability of the LRR fold. Unlike experimental multi-protein complexes that often contain unresolved residues, this template provided a structurally complete LRR domain, ensuring a continuous potential energy surface for MD simulations. This intact architecture, particularly the solvent-exposed beta-sheets, provides the necessary framework to accommodate the distinct geometric requirements of p27 and Akt1. In addition, p27 was built using similarity-based methods using the AlphaFold DB entry P46527.1. A as a structural template. The resulting p27 model displayed an architecture composed of 4 alpha-helices and 3 beta-strands, involving approximately 4% of the total residues, while the remaining sequence occupies extensive coil regions reflecting its natively disordered state. This layout supports its function as a cancer-inhibiting protein that loses stability upon SKP2-mediated UbK48-linked tagging. The reliability of this model was further substantiated by the Global Model Quality Estimate (GMQE), which confirmed 100.00% sequence identity to the native protein, validating that the substrate interface is structurally robust for investigating the atomistic determinants of p27 degradation. To validate the conformational nature of p27 (UniProt P46527), an intrinsic disorder prediction was performed using IUPred2A. As shown in Figure 2, the IUPred2 (long) score remains significantly above the 0.5 threshold for nearly the entire 198-residue sequence, peaking at 0.95 in the C-terminal region. This quantitative profile confirms that p27 is an intrinsically disordered protein (IDP). While the protein is predominantly unstructured, the ANCHOR2 profile identifies high-probability binding regions, notably between residues 110–150 and 175–198, where scores reach 0.96. These peaks represent functional ‘anchor’ motifs that undergo a disorder-to-order transition upon engagement with the SKP2 complex. This data reconciles the extensive coil-based architecture observed in our model with the protein’s native biological flexibility. This layout supports its function as a cancer-inhibiting protein, which loses stability once attacked by SKP2 via UbK48-linked tagging.

Moreover, UbK48 was analyzed through template-guided and machine-learning methods, resulting in a shape with 5 alpha helices, 8 beta strands, while including 2 coiled sections. Its basic layout aids its role in protein destruction, because linking to p27 helps mark it for processing. At the same time, Akt1’s kinase part was mapped using 6HHJ as reference; this showed a well-defined fold consisting of 96 residues (20%) partitioned into alpha-helices and 60 residues (13%) into beta-strands, connected by 103 residues (21%) located within loop zones, maintaining the characteristic bilayered kinase architecture. Such intricate arrangement highlights control abilities, especially once SKP2 triggers UbK63-type tagging. In comparison, UbK63 came from a solved crystal setup (PDB ID: 5GOJ), made up of 11 α-helices, alongside 29 β-strands and 41 coiled parts. Its shape fits well with non-degradative functions - attaching to Akt1 boosts enzyme activity instead of breaking it down.

Taken together, structural models and topology maps show clear differences in how secondary elements are arranged and how complex each protein’s shape is. These results establish a foundational framework for subsequent validation and mechanistic exploration.

### 3.2 Structural quality assessment (SQA) of proteins involved in SKP2-driven dual ubiquitination

The predicted models of SKP2, p27, UbK48, Akt1, and UbK63 were subjected to detailed structural evaluation, with the outcomes summarized in Table 1. Begin with, SKP2 demonstrated excellent stereochemical quality, supported by an ERRAT score of 94.97, 97.80% of residues in favored Ramachandran regions, and a MolProbity percentile of 100th percentile. Its ProQ LGscore of 11.38 and ProSA Z-score of −8.93 further reinforced the reliability of the model. Notably, the SKP2 Z-score falls within the range of structures typically determined by X-ray crystallography, consistent with its larger size and well-defined leucine-rich repeat (LRR) framework.

**TABLE 1:** Validation of modeled proteins using ERRAT, PROCHECK, MolProbity, ProQ, and ProSA. These analyses assess stereochemical quality, backbone geometry, and overall structural reliability, confirming the accuracy of the predicted protein models.

Protein	ERRAT (%)	PROCHECK (%)	MolProbity (Å)	ProQ (LGscore)	ProSA (z-score)
SKP2	94.97	97.80	0 100th percentile	11.38	−8.93
p27	76.27	98.46	1.54 94th percentile	5.32	−4.16
UbK48	100	95.5%	0.50 100th percentile	7.414	−6.79
AKT	95	96.89	0.71 99th percentile	7.01	−6.89
UbK63	98.36	98.63	3.06 96th percentile	7.08	−6.37

In contrast, p27 yielded comparatively moderate values, with an ERRAT score of 76.27, 98.46% favored residues, and a MolProbity percentile of 94th percentile. The ProQ LGscore of 5.32 and Z-score of −4.16 place it within the NMR-derived region of the ProSA plot. This transition from the X-ray to the NMR region across the different models is primarily a function of protein size; as Z-scores are length-dependent, smaller or more flexible proteins like p27 and Ubiquitin naturally exhibit scores closer to zero while remaining within the high-confidence bounds of native-like experimental structures. UbK48, on the other hand, achieved a perfect ERRAT score of 100% and 95.50% favored residues, accompanied by a ProQ LGscore of 7.41 and Z-score of −6.79.

Moving to Akt1, the kinase domain model displayed consistent reliability, with an ERRAT score of 95, 96% favored residues, and a MolProbity percentile of 99th percentile. Its ProQ LGscore (7.01) and Z-score (−6.89) aligned well with the structural complexity of its regulatory fold. Finally, UbK63, derived from crystallographic data, confirmed native-like folding with an ERRAT score of 98.36, 98.63% favored residues, a MolProbity percentile of 96th percentile, a ProQ LGscore of 7.08, and a Z-score of −6.73. Taken together, these validation metrics demonstrate that all five proteins exhibit acceptable stereochemical geometry, stable atomic packing, and fold reliability. Consequently, the validated models provide a solid foundation for mechanistic interpretation of SKP2-mediated dual ubiquitination.

### 3.3 Structural binding assessment of SKP2 with p27 and Akt1 under non-ubiquitinated conditions

To elucidate the structural basis of SKP2-mediated dual ubiquitination in breast cancer, docking simulations were performed to characterize its interaction interfaces with p27 and Akt1. The likely active spots in SKP2 are PHE20, ASP268, THR270, ARG294, LYS295, ARG344, LYS125, GLU271, GLU116, LYS119, ASN146, HIS148, ASP150, ASP171, GLN172, GLU194, SER196 - several stay identical across organisms yet point outward. On the other side, p27 holds critical points including PHE511, ASP159, TYR74, GLU75, GLY72, GLQ77, GLU80, GLU185, LYS73. At the same time, Akt1 features amino acids such as LYS251, DSP113, ASP134, ASP136, GLU79, ARG57, LYS135, TYR27, ARG25.

**FIGURE 3:**
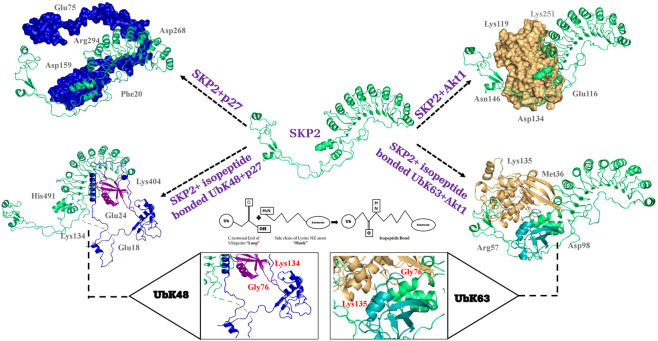
Molecular docking of SKP2 with p27 and Akt1 in the presence and absence of ubiquitin linkages. The panels illustrate binding orientations within conserved recognition pockets and substrate-specific binding cavities for four complexes: SKP2–p27, SKP2–UbK48–p27, SKP2–Akt1, and SKP2–UbK63–Akt1. Docking poses highlight the conserved residues that mediate substrate recognition as well as the distinct binding pockets remodeled by K48- and K63-linked ubiquitination. These structural comparisons emphasize how ubiquitin linkage type alters SKP2’s binding interface, thereby modulating substrate engagement and regulatory outcome.

Following docking and water refinement, the generated models were subjected to HADDOCK clustering based on RMSD criteria, yielding 14 clusters (85 structures) for SKP2–p27 and 7 clusters (90 structures) for SKP2-Akt1; detailed cluster statistics are provided in Supplementary Tables S1, S3. For the SKP2-p27 interaction, Cluster 2 was identified as the most probable binding pose, exhibiting a HADDOCK score of −103.8 ± 7.8 and a highly significant Z-score of −2.0. While the global RMSD for this cluster was 23.7 ± 7.4 Å, this value is a consequence of the structural architecture of the complex. SKP2 possesses a large, curved leucine-rich repeat (LRR) framework, while p27 is an inherently flexible, elongated protein. In such systems, minor conformational adjustments at the binding interface result in large atomic displacements at the distal, non-interfacial ends a “lever-arm” effect that geometrically inflates global RMSD without compromising the stability of the interface itself. The statistical significance of the Z-score (well below the −1.0 threshold) confirms that Cluster 2 is an energetically distinct and converged solution (Vangone and Bonvin, 2015). In contrast, SKP2–Akt1 showed a HADDOCK score of −85.1 ± 8.7 along with an RMSD of 8.4 ± 0.2 Å and a Z-score of −1.3, suggesting consistent interaction patterns. These results are listed in Table 2; meanwhile, Figure 3 presents key structural snapshots from the runs.

**TABLE 2:** Binding affinity and docking energy parameters for SKP2 complexes with p27 and Akt1 in the presence and absence of ubiquitin linkages.

Complex	Cluster analysis			Energy component breakdown					Protein-protein interface interactions analysis	
	HADDOCK score	RMSD (Å)	Z-score	VdW	Electrostatic	Desolvation	Restraint energy	BSA (Å^2^)	ΔG *(kcal/mol)*	Kd (M)
SKP2-p27	−103.8+/-7.8	23.7+/-7.4	−2.0	−51.1+/-6.3	−192.1+/-37.9	−23.1+/-7.1	89.1+/-61.8	1838.3+/-200.2	−10.8	1.2 × 10^−8^
SKP2-UbK48-p27	−120.9+/-2.1	0.3+/-0.2	−1.9	−62.6+/-2.1	−168.3+/-34.7	−32.7+/-2.5	79.9+/-19.9	1900.5+/-68.6	−10.6	1.6 × 10^−8^
SKP2–Akt1	−85.5+/-8.7	8.4+/-0.2	−1.3	−42.0+/-3.4	−323.6+/-32.9	2.2+/-4.6	190.2+/-54.3	1964.0+/-101.3	−10.6	1.6 × 10^−8^
SKP2-UbK63-Akt1	−78.1+/-0.8	7.2+/-0.0	−1.2	−39.7+/-1.0	−338.1+/-8.4	14.3+/-1.3	149.5+/-22.3	1895.7+/-27.5	−10.6	1.8 × 10^−8^

Reported values include HADDOCK score, RMSD, Z-score, van der Waals, electrostatic, desolvation, restraint violation energy, buried surface area (BSA), ΔG, and Kd, providing a comparative assessment of interaction strength and complex stability.

Energetic decomposition revealed that SKP2–p27 binding was driven by strong electrostatic forces (−192.1 ± 37.9) and favorable desolvation (−23.1 ± 7.1). For SKP2-Akt1, electrostatic energy contributed −323.6 ± 32.9, with desolvation energy of 2.2 ± 4.6. The buried surface area came out to 1838.3 ± 200.2 Å^2^ in the SKP2–p27 pair, whereas it reached 1964.0 ± 101.3 Å^2^ for SKP2–Akt1 - both showing broad interaction zones. The divergence in desolvation energy between the two complexes, despite their similar BSA values, reflects the distinct physicochemical properties of their respective interfaces. The positive desolvation energy in the SKP2-Akt1 complex correlates with its significantly more negative electrostatic component, suggesting an interface dominated by charged residues that carry a high hydration penalty during complex formation. In contrast, the highly favorable negative desolvation in SKP2-p27 indicates a more hydrophobic driving force, likely involving the leucine-rich repeat (LRR) framework of SKP2, which promotes a favorable entropic gain upon the release of water molecules. While one complex displays slightly lower values, the other demonstrates tighter packing due to more contact points between subunits.

**FIGURE 4:**
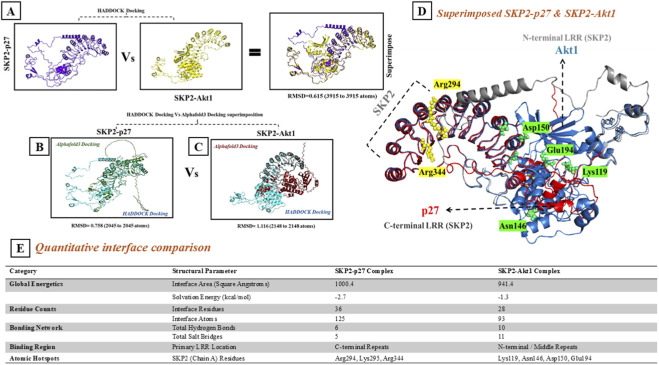
Comparative Structural and Quantitative Interface Analysis of SKP2–Substrate Complexes. **(A)** Superimposition-based comparative analysis of SKP2-p27 (purple) and SKP2-Akt1 (yellow) complexes obtained from HADDOCK docking. Alignment of the conserved SKP2 backbone shows an RMSD of 0.615 Å highlights the distinct spatial orientations of the intrinsically disordered p27 versus the globular Akt1 kinase on the same scaffold. **(B)** Structural validation of the SKP2-p27 complex. Superimposition of the HADDOCK-predicted pose (cyan) with the AlphaFold3 model (green) shows an RMSD of 0.758 Å confirms a highly consistent and reliable binding mode across independent methodologies. **(C)** Structural validation of the SKP2-Akt1 complex. Superimposition of the HADDOCK model (cyan) with the AlphaFold3 model (red) yields a RMSD of 1.116 Å demonstrates a strong structural convergence and validating the predicted interface. **(D)** Atomic-level mapping of divergent binding footprints. A detailed 3D superimposition reveals the physical basis for differential ubiquitination. The p27 substrate (red) is anchored at the C-terminal LRR repeats of SKP2, stabilized by a rigid hotspot involving Arg294 and Arg344 (yellow spheres). Conversely, the Akt1 substrate (blue) shifts toward the N-terminal/Middle LRR repeats, utilizing a distinct network of residues including Lys119, Asn146, Asp150, and Glu194 (green spheres). This spatial shift reorients the substrate to favor K48-linked (p27) versus K63-linked (Akt1) chain assembly. **(E)** Tabulated data derived from PDBePISA analysis summarizes the global energetics and residue-level parameters for both complexes. The analysis highlights the larger interface area of p27 (1000.4 Å), significant denser hydrogen bond and salt bridge network of Akt1, reveals the thermodynamic and structural rationale for their specific linkage preferences.

Further, to clarify the molecular recognition differences between SKP2 with p27 and Akt1 at the atomic level, we performed a comparative structural superimposition of the docking solutions (Figure 4). By aligning the conserved SKP2 backbone (Chain A), we achieved a remarkably low RMSD of 0.615 Å (3,915 atoms), establishing a stable structural framework for side-by-side assessment. This superimposition reveals that while both substrates target the concave surface of the SKP2 leucine-rich repeat (LRR) domain, they adopt distinct orientations and engage different “atomic hotspots” (Figures 4A,D). These divergent binding footprints demonstrate SKP2’s capacity to accommodate chemically diverse substrates ranging from the intrinsically disordered p27 to the globular Akt1 kinase through a flexible recognition mechanism.

To address the concern regarding potential bias from HADDOCK-defined active sites, we independently validated the docking poses using AlphaFold 3 (AF3). This “blind” folding approach, which does not require predefined residues, showed exceptional structural convergence with our physics-based models.

For the SKP2-p27 complex, the HADDOCK pose and the top-ranked AF3 model (Model 0) exhibited an all-atom RMSD of 0.758 Å (2,045 atoms) (Figure 4B). Similarly, the SKP2-Akt1 complex demonstrated high-confidence agreement, with an all-atom RMSD of 1.116 Å (2,148 atoms) (Figure 4C). The high degree of structural similarity (RMSD < 1.5 Å) between two fundamentally different methodologies HADDOCK’s interaction-driven docking and AF3’s deep-learning-based folding confirms that the predicted interfaces are structurally robust and biologically relevant (Lensink and Wodak, 2010; Rettie et al., 2025). This dual-validation approach provides a high-confidence structural foundation for the subsequent molecular dynamic’s simulations.

To further quantify these divergent binding modes, the superimposed SKP2-p27 and SKP2-Akt1 structures were visualized in PyMOL, followed by a comprehensive interface analysis using PDBePISA (Figures 4D,E). The analysis revealed that p27 is anchored primarily at the C-terminal LRR repeats of SKP2, forming a compact and well-defined interaction network involving 36 interface residues and 125 atoms, with an interface area of 1000.4 Å^2^ and a solvation energy of −2.7 kcal/mol. This interaction is stabilized by a focused network of 6 hydrogen bonds and 5 salt bridges, centered around key hotspot residues such as Arg294, Lys295, and Arg344.

In contrast, Akt1 shifts its docking footprint toward the N-terminal and middle LRR repeats, engaging a more distributed and extensive interaction network comprising 28 interface residues and 93 atoms, with an interface area of 941.4 Å^2^ and a solvation energy of −1.3 kcal/mol. This complex exhibits a denser bonding pattern with 10 hydrogen bonds and 11 salt bridges, involving key residues such as Lys119, Asn146, Asp150, and Glu194.

This spatial divergence observed in the superimposed structures leads to distinct substrate orientations on the SKP2 scaffold, thereby defining different interface architectures. Such differences in binding region, residue composition, and interaction networks determine how each substrate is positioned relative to the ubiquitination machinery. Because ubiquitination specificity depends on substrate positioning and interaction geometry, these structural differences provide a plausible basis for the preference toward K48-linked ubiquitination in SKP2–p27 and K63-linked ubiquitination in SKP2-Akt1, representing a key novel aspect of our study.

These results show SKP2 binds p27 and Akt1 using different shapes. Such selective binding backs the main idea in breast cancer, SKP2 enables double tagging for breakdown by linking tightly to various targets, thus affecting how cells divide and grow abnormally.

### 3.4 Structural effects of ubiquitin isopeptide conjugation on SKP2 binding to p27 and Akt1

**FIGURE 5:**
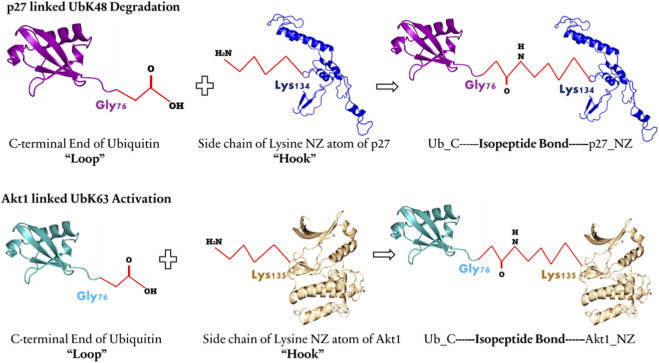
Computational modeling of isopeptide-linked ubiquitin conjugates with substrate proteins p27 and Akt1. The structural modeling approach used to construct the ubiquitin–substrate isopeptide linkage representing the post-ubiquitination (post-transfer) state relevant to SKP2-mediated regulation in breast cancer. The C-terminal Gly76 of ubiquitin was covalently linked to the ε-amino group of Lys134 of p27 and Lys135 of Akt1 using a loop-and-hook configuration to achieve an appropriate spatial orientation. The resulting isopeptide bond geometry is consistent with native ubiquitin conjugation, enabling structural analysis of SKP2 interactions with K48-linked ubiquitin–p27 and K63-linked ubiquitin-Akt1 conjugates.

To represent the post-ubiquitination (post-transfer) state relevant to degradation (K48-linked Ubiquitin) and signaling activation (K63-linked Ubiquitin), isopeptide bonds were successfully constructed between the C-terminal Gly76 of ubiquitin and the ε-amino group of the target lysine’s Lys134 on p27 and Lys135 on Akt1 forming UbK48–p27 and UbK63–Akt1 conjugates, respectively. As shown in Figure 5, manual bond construction using PyMOL and Chimera yielded a stable C-N amide linkage consistent with native ubiquitination geometry.

During isopeptide bond construction, deletion of the OXT atom from Gly76 and one NZ hydrogen from the substrate lysine created a chemically favorable environment for amide linkage. Both modeling platforms generated identical topologies for the Gly76–Lys bond, confirming reproducibility. Subsequent loop refinement in MODELLER resolved steric clashes near the linkage site, improved local Ramachandran statistics, reduced backbone strain, and corrected side-chain orientations around the modified lysines.

Energy minimization using AMBER/CHARMM force-field tools further stabilized the isopeptide region. Standard C–N bond lengths (≈1.32–1.35 Å) and restored amide planarity were observed, with side-chain torsions falling within expected ranges. No steric overlaps remained in either complex. Chemical validation in Avogadro confirmed correct atom types, hybridization states, and amide topology, with no valence or geometry violations detected. Both conjugates exhibited full chemical integrity.

The final optimized structures were exported for molecular docking with SKP2, treating each conjugate as a single covalently linked entity. Visual inspection Chimera and PyMOL showed the isopeptide area kept its shape throughout. Following water refinement and RMSD-based clustering, HADDOCK grouped the SKP2-ubiquitin-p27 docking solutions into 10 clusters comprising 92 structures, whereas the SKP2-ubiquitin-Akt1 complex yielded 8 clusters comprising 88 structures. Detailed cluster statistics, including cluster size, HADDOCK scores, RMSD distributions, and Z-scores, are provided in Supplementary Tables S2, S4. As shown in Table 2 together with Figure 3, docking results revealed notable binding for both complexes. The SKP2-UbK48-p27 complex yielded a HADDOCK score of −120.9 ± 2.1, accompanied by a remarkably low RMSD of 0.3 ± 0.2 Å and a Z-score of −1.9, indicating a highly stable and well-defined interaction. By contrast, the SKP2-UbK63-Akt1 complex produced a comparatively weaker HADDOCK score of −78.1 ± 0.8, with a substantially higher RMSD of 7.2 ± 0.0 Å and a Z-score of −1.2, reflecting reduced structural convergence and lower binding stability.

Energy estimates showed substantial van der Waals interactions (−62.6 ± 2.1; −39.7 ± 1.0), whereas electrostatic contributions were favorable as well (−168.3 ± 34.7; −338.1 ± 8.4). However, restraint energies were moderate (79.9 ± 19.9; 149.5 ± 22.3), whereas buried nonpolar surface was large throughout (1900.5 ± −68.6 Å^2^; 1895.7 ± 27.5). Results indicate SKP2 binds ubiquitin-tagged targets via robust, wide interfaces. This interplay aligns with its function in enabling two-step ubiquitination.

Overall, these results indicate that SKP2 engages post-translationally modified (ubiquitin-conjugated) substrates through broad and energetically favorable interfaces, consistent with its established role in ubiquitin-mediated substrate recognition and processing.

### 3.5 Interface characterization and binding affinity estimation of docked complexes

Analysis of the docked complexes revealed a network of stabilizing contacts that define SKP2’s interaction with its diverse substrates. In the SKP2-p27 pair, the interface is characterized by key hydrogen bonds between PHE20-ASP159 (2.94 Å), ASP268-TYR74 (2.79 Å), and LYS295-GLU72 (3.04 Å). Crucially, a highly stable salt bridge was identified at ARG344-GLU80 (2.60 Å), a site consistent with the established substrate-binding pocket in the Leucine-Rich Repeat (LRR) domain of SKP2. Upon covalent attachment of ubiquitin (UbK48-p27), the core binding architecture remained stable, supplemented by a new auxiliary contact between HIS491 and GLU64 (3.53 Å). This suggests that K48-linkage preserves the canonical orientation required for proteasomal degradation. For the SKP2-Akt1 interaction, the interface was anchored by a LYS119-ASP113 hydrogen bond and salt bridge network (2.61 Å), alongside a cluster of GLN172-TYR27 interactions (2.76 Å). These residues are localized within the Akt1 Pleckstrin Homology (PH) domain, which is the experimentally validated target for SKP2-mediated K63-linked ubiquitination. In the SKP2-UbK63-Akt1 complex, these foundational contacts were retained, while an additional ASP133-LYS135 salt bridge (2.62 Å) emerged. This broadening of the interaction surface following ubiquitin attachment supports a mechanism where K63-linkage facilitates Akt1 membrane recruitment and activation rather than degradation. These residue-level networks are summarized in Table 3 and visualized in Supplementary Figure S1, depicting the precise spatial distribution of stabilizing forces across the SKP2 binding cleft.

**TABLE 3:** Residue-residue contacts, interaction categories, and closest atomic distances mapped at SKP2 protein-protein interfaces.

Complex	Residue 1 (chain A)	Residue 2 (chain B)	Interaction types	Closest distance (Å)
SKP2–p27	PHE20	ASP159	Hydrogen bond	2.94
	ASP268	TYR74	Hydrogen bond	2.79
	THR270	TYR74	Hydrogen bond	2.76
	ARG294	GLU75	Hydrogen bond	2.76
	LYS295	GLY72	Hydrogen bond	3.04
	ARG344	GLN77	Hydrogen bond	3.14
	ARG344	GLU80	Hydrogen bond, Salt bridge	2.60
	LYS125	GLU185	Salt bridge	3.56
	GLU271	LYS73	Salt bridge	3.69
SKP2-UbK48-p27	PHE511	HIS68	Hydrogen bond	2.87
	LYS404	GLU24	Salt bridge	2.44
	LYS483	GLU18	Salt bridge	2.04
	HIS491	GLU64	Salt bridge	3.53
SKP2–Akt1	GLU116	LYS251	Salt bridge	3.63
	LYS119	ASP113	Hydrogen bond, Salt bridge	2.61
	ASN146	ASP134	Hydrogen bond	2.73
	ASN146	ASP136	Hydrogen bond	3.14
	HIS148	GLU79	Hydrogen bond, Salt bridge	2.79
	ASP150	ARG57	Hydrogen bond, Salt bridge	2.62
	ASP171	LYS135	Hydrogen bond, Salt bridge	2.63
	GLN172	TYR27	Hydrogen bond	2.76
	GLU194	ARG25	Hydrogen bond, Salt bridge	2.60
	SER196	ARG25	Hydrogen bond	3.12
SKP2-UbK63-Akt1	MET36	SER247	Hydrogen bond	3.10
	ASP98	ARG333	Hydrogen bond, Salt bridge	2.60
	GLU103	ARG25	Hydrogen bond, Salt bridge	2.63
	LYS125	SER110	Hydrogen bond	2.79
	ASP133	LYS135	Hydrogen bond, Salt bridge	2.62
	ARG84	ASP153	Salt bridge	3.83

The data capture structural rearrangements across both axes: reduced stabilizing contacts in the SKP2–p27 complex upon K48-linked ubiquitination, and enhanced interface interactions in the SKP2–Akt1 complex upon K63-linked ubiquitination. Together, these comparisons highlight how linkage type remodels interface architecture and dictates complex stability.

Binding affinity analysis with PRODIGY (Table 2) conducted on the representative models from the top-ranked docking clusters, revealed that SKP2 engages its substrates with consistently high-affinity energetics. The SKP2-p27 pair demonstrated the highest stability, with a free energy of −10.8 kcal/mol and a Kd of 1.2 × 10^−8^ M, providing evidence of a robust fit for the native substrate. Following the attachment of the K48-ubiquitin linker, the binding affinity remained nearly identical (ΔG of −10.6 kcal/mol; Kd of 1.6 × 10^−8^ M), indicating that high-affinity compatibility persists post-ubiquitination. Similarly, the SKP2-Akt1 pairing showed a binding energy of −10.6 kcal/mol (Kd of 1.6 × 10^−8^ M), while the UbK63-linked Akt1 assembly yielded comparable values (ΔG of −10.6 kcal/mol; Kd of 1.8 × 10^−8^ M).

These minor numerical shifts suggest a thermodynamic trade-off, where the increase in interfacial interaction pairs and electrostatic anchoring is balanced by a concomitant desolvation penalty. Collectively, these results highlight that ubiquitylation broadens the interaction surface and reinforces the structural stability of the docking station while maintaining a consistent low-nanomolar binding affinity across both target systems. Supported by contact patterns in Table 3, results highlight SKP2’s flexibility and consistent nanomolar affinity despite different targets.

### 3.6 MD analysis shows opposite structural effects of K48 and K63 ubiquitination on SKP2

**FIGURE 6:**
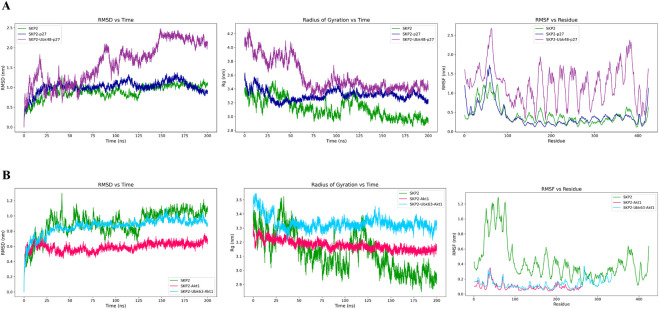
Comparative dynamic stability profiles of SKP2 complexes assessed by RMSD, radius of gyration (Rg), and RMSF over 200 ns simulations. **(A)** SKP2 alone, SKP2–p27, and SKP2–UbK48–p27 complexes. The plots highlight progressive increases in RMSD and Rg together with elevated residue-level fluctuations in the K48-ubiquitinated complex, consistent with conformational loosening and degradation-oriented destabilization. **(B)** SKP2 alone, SKP2–Akt1, and SKP2-UbK63-Akt1 complexes. Here, RMSD and Rg values remain comparatively lower, and RMSF fluctuations are minimized, indicating that K63-linked ubiquitination stabilizes the SKP2-Akt1 interface and supports an activation-favoured conformation.

In this study, RMSD, RMSF, and radius of gyration (Rg) were used to assess the structural stability and conformational dynamics of SKP2 in different modeled states. Average results (mean ± SD) appear in Table 4, while Figure 6A shows movement patterns for SKP2-p27, whereas Figure 6B displays them for SKP2–Akt1, allowing side-by-side assessment of both ubiquitination routes.

**TABLE 4:** Summary of molecular dynamics outcomes for SKP2 and Its Complexes.

MD parameter	Definition/Equation	Duration (Nano-second)	SKP2	SKP2–p27	SKP2-UbK48-p27	SKP2-Akt1	SKP2-UbK63-Akt1
RMSD (Å) *Root Mean Square Deviation*	Measures overall structural deviation	200ns	0.90 ± 0.16	1.01 ± 0.15	1.61 ± 0.50	0.59 ± 0.05	0.86 ± 0.10
RMSF (Å) *Root Mean Square Fluctuation*	Residue-wise flexibility	200ns	0.43 ± 0.25	0.40 ± 0.28	1.23 ± 0.46	0.09 ± 0.04	0.13 ± 0.05
Rg (nm) *Radius of Gyration*	Compactness of the structure	200ns	3.12 ± 0.13	3.30 ± 0.07	3.57 ± 0.22	3.17 ± 0.03	3.33 ± 0.04
SASA (nm^2^) *Solvent Accessible Surface Area*	Protein surface area exposed to solvent	200ns	227.71 ± 8.34	388.12 ± 15.20	428.90 ± 16.26	351.59 ± 8.05	395.99 ± 5.98
H-bonds (count) *Hydrogen Bonds*	Number of intermolecular H-bonds	200ns	—	17.67 ± 4.57	10.96 ± 3.94	12.91 ± 2.54	14.42 ± 3.34

This table presents the averaged structural and interaction metrics obtained from the MD simulations of SKP2 and its different complex states. The values reflect overall stability, flexibility, conformational compactness, solvent exposure, and intermolecular interaction strength across the trajectories, allowing comparative evaluation of how each binding partner influences SKP2 dynamics.

Looking at RMSD, SKP2 by itself stayed steady (0.90 ± 0.16 nm). Once bound to p27, values went up slightly (1.01 ± 0.15 nm), suggesting some structural adjustment. The shift was markedly clearer in the SKP2-UbK48-p27 setup, where RMSD reached 1.61 ± 0.50 nm. This significant rise indicates structural “loosening” specifically tied to tagging for degradation, providing the necessary conformational freedom for proteasomal engagement. In contrast, SKP2-Akt1 interactions by themselves (0.09 ± 0.04 nm) and with UbK63 (0.13 ± 0.05 nm) displayed minimal movement, indicating a more compact structure optimized for stable signaling.

Regional changes detected via RMSF confirmed these findings. Although SKP2 shifted slightly (0.42 ± 0.25 nm), its link with p27 remained stable (0.40 ± 0.28 nm). In contrast, when UbK48 joined the SKP2-p27 complex, motion spiked significantly (1.23 ± 0.46 nm). This “Flexible-Signal” represents a biological prerequisite for degradation, as the proteasome requires a partially destabilized substrate to initiate unfolding. On the other hand, SKP2-Akt1 remained rigid (0.09 ± 0.04 nm); adding UbK63 caused only a negligible change (0.13 ± 0.05 nm). This “Stable-Lock” configuration maintains the tighter, active shape required for signal transduction.

In line with these findings, Rg analysis provided additional mechanistic insight. SKP2 by itself formed a tight shape (3.12 ± 0.13 nm). When linked to p27, it became slightly larger (3.30 ± 0.07 nm), but this expansion increased significantly with the attachment of UbK48 (3.57 ± 0.22 nm). This increased Rg hints at the more open, “accessible” forms required for substrates tagged for proteasomal breakdown. Conversely, SKP2-Akt1 kept a dense configuration (3.17 ± 0.03 nm), while the UbK63 version showed only mild stretching (3.33 ± 0.04 nm), matching the precise structural shifts tied to signal triggering.

To validate the reliability of the initial docking poses, we monitored the spatiotemporal stability of key intermolecular interactions across the 200 ns MD trajectories. This analysis confirms that the critical hydrogen bonding networks identified during the static docking phase are not transient but persist under dynamic conditions.

As summarized in Table 5, the core “anchor” interactions for all four complexes remained remarkably intact. For the SKP2-p27 and SKP2-UbK48-p27 assemblies, primary contacts such as ARG294-GLU75 and LYS483-GLU18 were maintained throughout the simulation. Similarly, the SKP2-Akt1 and SKP2-UbK63-Akt1 complexes exhibited high interfacial conservation, with the ASP171-LYS135 and ASP98-ARG333 bonds persisting from the initial docking through the final 200 ns snapshot.

**TABLE 5:** Spatiotemporal stability of key protein-protein interfaces during MD simulations: This table highlights the persistence of critical contacts initially identified via molecular docking across the MD trajectories.

Complex	Interaction type	Snapshots at different time duration			
		50 ns	100 ns	150 ns	200 ns
SKP2-p27	Hydrogen bond	ARG294-GLU75 (2.640 Å)	PHE20-ASP158 (3.126 Å)	PHE20-ASP159 (2.588 Å)	GLU271-LYS73 (2.754 Å)
SKP2-UbK48-p27	Hydrogen bond	LYS483-GLU18 (2.857 Å)	LYS483-GLU18 (3.135 Å)	PHE511-HIS68 (2.894 Å)	LYS483-GLU18 (2.727 Å)
SKP2-Akt1	Hydrogen bond	ASP171-LYS135 (2.759 Å); ASP150-ARG57 (2.693 Å); LYS119-ASP113 (2.823 Å)	GLU194-ARG25 (2.747 Å); ASP171-LYS135 (2.664 Å)	ASP150-ARG57 (3.984 Å); GLU194-ARG25 (2.779 Å); ASP171-LYS135 (2.926 Å); LYS119-ASP113 (2.617 Å)	LYS119-ASP113 (2.881 Å); GLU194-ARG25 (2.518 Å); ASP171-LYS135 (2.631 Å); ASP150-ARG57 (2.767 Å)
SKP2-UbK63-Akt1	Hydrogen bond	ASP98-ARG333 (2.847 Å); ARG84-ASP153 (2.909 Å)	ASP98-ARG333 (2.670 Å); ARG84-ASP153 (2.807 Å)	ARG84-ASP153 (2.616 Å)	ASP98-ARG333 (2.729 Å); ARG84-ASP153 (2.693 Å)

The sustained presence of these interactions throughout the simulation period validates the structural integrity and high-confidence stability of the predicted protein-protein interfaces under dynamic conditions.

The visual evolution of these interfaces, represented in the snapshots below Table 5, illustrates the transition from the static docking pose to a refined, dynamic binding state. While the initial docking contacts remained stable, the MD trajectories also revealed the formation of new, supplementary interactions that further optimized the binding interfaces over time. These emergent contacts, which provide additional structural reinforcement, are detailed in Supplementary Tables S5–S8, along with corresponding interaction figures at each time point (50, 100, 150, and 200 ns).

The sustained presence of the docking-derived contacts, coupled with the recruitment of new stabilizing bonds, confirms that the predicted interfaces are energetically favorable and structurally robust across the entire simulation window.

These connected findings show two different structural paths. UbK48-linked ubiquitination induces higher flexibility and interfacial instability to facilitate its function in proteasome breakdown. In contrast, the UbK63 modification acts as a mechanical stabilizer that preserves a tight structure, thereby “locking” the complex into a ready-to-activate state. Thus, the distinct structural signatures we observed reinforce our central conclusion that dual ubiquitination channels SKP2 toward either degradation or activation through separate conformational pathways.

### 3.7 SASA and hydrogen-bond analysis reveals distinct stabilization patterns between UbK48- and UbK63-Linked SKP2 complexes

**FIGURE 7:**
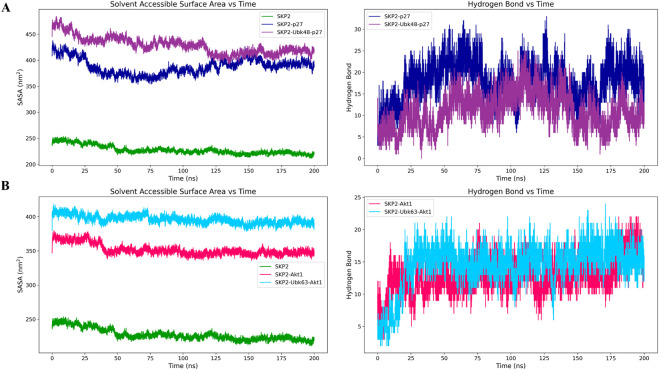
Solvent accessible surface area (SASA) and hydrogen-bond (H-bond) dynamics of SKP2 complexes over 200 ns simulations. **(A)** SASA profiles of SKP2 alone, SKP2-p27, and SKP2-UbK48-p27, together with H-bond counts for SKP2-p27 and SKP2-UbK48-p27. The progressive rise in SASA values, coupled with reduced H-bonding in the K48-ubiquitinated complex, reflects structural loosening and destabilization consistent with proteasomal degradation. **(B)** SASA profiles of SKP2 alone, SKP2-Akt1, and SKP2-UbK63-Akt1, along with H-bond counts for SKP2-Akt1 and SKP2-UbK63-Akt1. Here, SASA increases are accompanied by enhanced H-bonding in the K63-ubiquitinated complex, indicating a stable yet solvent-exposed architecture that supports Akt1-mediated activation.

In our analysis, the SASA and hydrogen-bond (H-bond) profiles provided additional clarity on how p27-and Akt1-associated ubiquitination modulates SKP2 complex stability. The averaged values (mean ± SD) are summarized in Table 4, while the dynamic trajectories are illustrated in Figure 7A for the SKP2-p27 axis and Figure 7B for the SKP2-Akt1 axis.

#### 3.7.1 SASA analysis

SASA reflects the extent of solvent exposure and compactness of a protein complex during simulation. A progressive increase in SASA indicates structural expansion and reduced packing, whereas lower values correspond to compact and stable conformations. Along the p27 ubiquitination axis (Figure 7A), we observed that SKP2-p27 exhibited a marked increase in SASA (388.12 ± 15.20 nm^2^) compared to SKP2 alone (227.71 ± 8.35 nm^2^), highlighting structural expansion upon substrate binding. This effect was further amplified in the SKP2-UbK48-p27 complex, where SASA rose to 428.90 ± 16.26 nm^2^. Such enhanced solvent exposure is consistent with a degradation-oriented conformation, supporting the notion that UbK48-linked ubiquitination destabilizes SKP2 for proteasomal processing. In contrast, the Akt1 ubiquitination axis (Figure 7B) revealed a different trajectory. SKP2-Akt1 showed a substantial increase in SASA (351.59 ± 8.05 nm^2^), reflecting a more open interface relative to SKP2 alone. Upon adding UbK63-linked ubiquitin, SASA rose slightly to 395.99 ± 5.98 nm^2^. The pattern - SKP2 alone, then SKP2-Akt1, followed by SKP2-UbK63-Akt1 - matches what is typically seen in cells, since UbK63 modification tends to expose more surface to water without disrupting structure, which supports protein activity instead of breakdown.

#### 3.7.2 Hydrogen-bond dynamics

Hydrogen bonds are critical determinants of inter-protein stability and recognition. Consistent with the dynamic plots, the averaged H-bond values in Table 4 revealed distinct patterns between degradation-associated and activation-associated complexes. On the p27 axis (Figure 7A), we observed that SKP2-p27 maintained strong inter-protein interactions, averaging 17.67 ± 4.57 hydrogen bonds. With UbK48-linked ubiquitin added, bond count fell to 10.96 ± 3.94. Such decrease suggests fewer stabilizing interactions; it aligns with instability seen in complexes marked for proteasome degradation.

The Akt1 axis (Figure 7B) revealed a contrasting pattern. SKP2-Akt1 formed 12.91 ± 2.54 hydrogen bonds, consistent with a moderately stable interface. With UbK63 ubiquitination, the bond count rose to 14.42 ± 3.34, indicating that additional stabilizing contacts were established. This increase supports the idea that UbK63 modification strengthens the SKP2-Akt1 complex, favoring an activation-biased conformation. For clarity, we note that values for SKP2 alone are not reported, since hydrogen bonds were calculated only for inter-molecular interactions.

Our SASA and H-bond analyses highlight two clearly divergent structural outcomes. Along the p27 axis, UbK48-linked ubiquitination produced markedly higher SASA values while simultaneously reducing the number of hydrogen bonds. This combination points to structural loosening and destabilization, a profile consistent with proteasomal degradation. In contrast, the Akt1 axis displayed the opposite tendency: UbK63-linked ubiquitination was associated with elevated SASA together with an increase in hydrogen bonding. This pattern reflects a stable yet solvent-exposed complex, supporting Akt1-mediated activation. Collectively, these observations demonstrate that SKP2 is directed toward distinct conformational fates degradation under UbK48 modification and activation under UbK63 modification.

### 3.8 PCA and FEL reveal distinct conformational signatures of UbK48- and UbK63-linked ubiquitination

**FIGURE 8:**
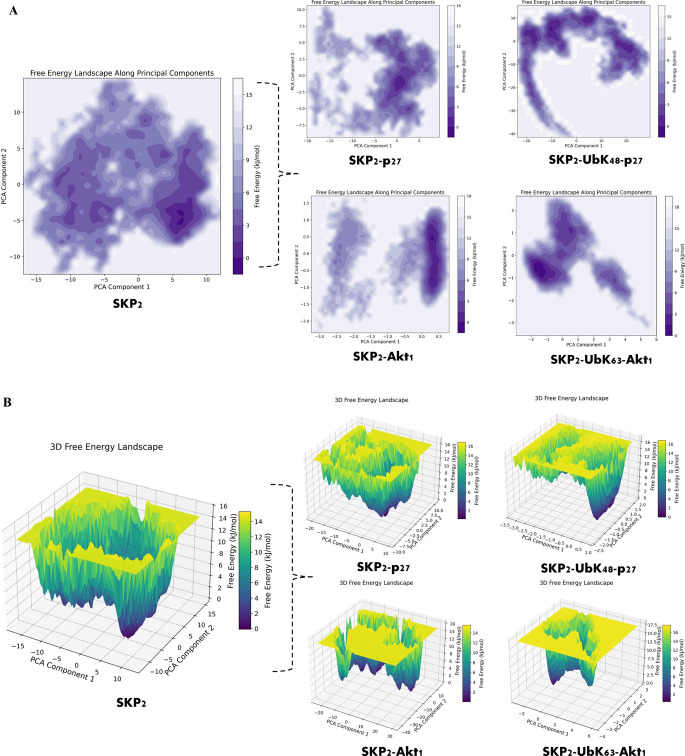
**(A)** Two-dimensional free energy landscapes (FEL) mapped onto PC1 and PC2 for SKP2 and its complexes. SKP2 alone explores a wide conformational space, whereas p27 binding restricts its motions, with K48-linked ubiquitination producing deeper minima indicative of a more degradation-prone state. In contrast, Akt1 binding generates a compact conformational basin that becomes further stabilized by K63-linked ubiquitination, reflecting an activation-oriented structural shift. **(B)** Corresponding 3D FEL surfaces showing the depth and distribution of energy minima across systems. SKP2-p27 and SKP2-UbK48-p27 exhibit increasingly stabilized wells, while SKP2-Akt1 and SKP2-UbK63-Akt1 display broader but energetically favorable basins. Together, the landscapes demonstrate that ubiquitin linkage type distinctly remodels SKP2 dynamics toward either degradation (K48) or activation (K63).

To decipher how ubiquitin linkage type modulates SKP2-substrate recognition and regulatory outcomes, we performed principal component analysis (PCA) together with free energy landscape (FEL) mapping across the full 200 ns molecular dynamics trajectories for SKP2 alone and in complexes with p27 and Akt1 (Figures 8A,B). By integrating these approaches, we were able to visualize the dominant collective motions and the corresponding energetic preferences that define the conformational space of each system.

During the 200 ns simulation, SKP2 in its unbound state exhibited a highly dispersed PCA distribution accompanied by a broad, multi-basin FEL. This profile reflects extensive structural plasticity and the absence of a dominant low-energy conformation. Once p27 was introduced, however, the wide ensemble collapsed into a more compact and energetically focused landscape, suggesting that p27 imposes conformational restraint on SKP2. Incorporation of UbK48-linked ubiquitin further remodeled this ensemble, producing a narrow, funnel-shaped low-energy basin in both 2D and 3D FEL profiles. The deep and sharply defined minimum indicates stabilization of a single degradation-competent conformation, consistent with the canonical role of UbK48 chains in marking substrates and in this case the SKP2–p27 recognition interface for proteolytic processing.

In contrast, the Akt1 axis revealed a markedly different conformational regime over the same 200 ns window. The SKP2-Akt1 system formed a well-defined, centralized low-energy well, signifying a more stable interaction compared to SKP2-p27. When UbK63-linked ubiquitin was introduced, this stabilization was further intensified, generating a broader but deeper energy basin with reduced conformational dispersion in the PCA plot. Such a pattern suggests that UbK63 ubiquitination does not restrict SKP2 into a degradation-targeted configuration; instead, it reinforces a structurally coherent, activation-favoured architecture that enables prolonged engagement with Akt1.

Viewed collectively, the PCA-FEL analyses across 200 ns simulations reveal a mechanistic dichotomy. UbK48-linked ubiquitination drives SKP2 toward a highly ordered, energetically minimized conformation compatible with p27 turnover, whereas UbK63-linked ubiquitination enhances conformational stabilization of SKP2-Akt1, supporting an activation-oriented functional state. These structural insights provide a mechanistic explanation for the differential biological outcomes observed experimentally, demonstrating that the ubiquitin-linkage code directly encodes distinct conformational states that govern SKP2’s substrate selection and regulatory fate.

### 3.9 UbK48 and UbK63 ubiquitination show opposing effects in MM/PBSA analysis

To clearly understand how ubiquitination influences SKP2 interactions, MM/PBSA binding free energy calculations were performed for four systems: SKP2-p27, SKP2-UbK48-p27, SKP2-Akt1, and SKP2-UbK63-Akt1. In all calculations, SKP2 (6742 atoms) was defined as molecule A, while the substrate was treated as a single molecular entity (molecule B): p27 (3031 atoms), UbK48-p27 (4262 atoms), Akt1 (4222 atoms), and UbK63-Akt1 (5443 atoms). Thus, the reported binding free energies represent only the non-covalent interaction between SKP2 and the entire substrate.

**FIGURE 9:**
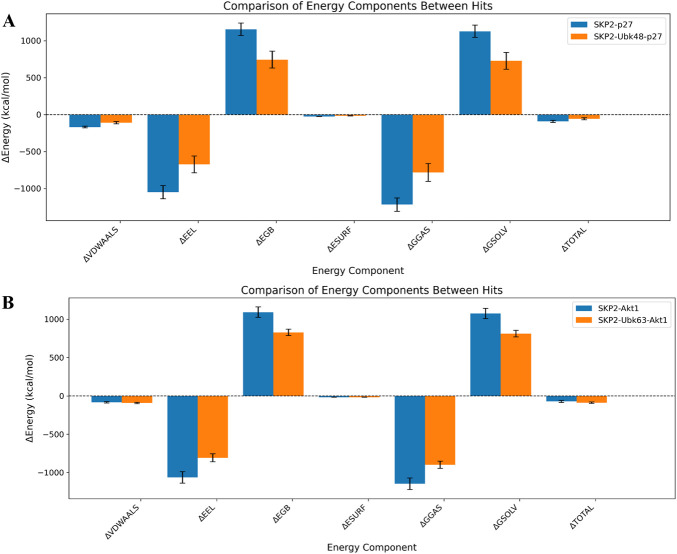
MM/PBSA binding free energy evaluation of SKP2 complexes. **(A)** Energy decomposition for SKP2-p27 and SKP2-UbK48-p27, showing that K48-linked ubiquitination reduces the binding affinity and destabilizes the SKP2-p27 interaction. **(B)** Energy profiles for SKP2-Akt1 and SKP2-UbK63-Akt1, indicating that K63-linked ubiquitination enhances intermolecular interactions and stabilizes the SKP2-Akt1 complex.

The calculated energetics (Table 6) show that SKP2-p27 forms a stable complex (−92.73 kcal/mol), primarily driven by strong electrostatic interactions (ΔEEL = −1050.55 kcal/mol). Structurally, p27 fits well within the SKP2 interface, as shown in Figure 9A. Upon K48-linked ubiquitination, the binding affinity decreases (−61.63 kcal/mol), with a marked reduction in electrostatic interactions (−1050.55 to −702.14 kcal/mol). As observed in Figure 9A, the addition of K48-linked ubiquitin introduces perturbations at the binding interface, leading to destabilization of the SKP2–p27 complex.

**TABLE 6:** Comprehensive MM/PBSA binding free-energy decomposition and comparative analysis of SKP2 interactions with p27 and Akt1 in their ubiquitinated and non-ubiquitinated states.

Energy component	SKP2-p27 (Average)	SKP2-UbK48-p27 (Average)	SKP2-Akt1 (Average)	SKP2-UbK63-Akt1 (Average)
➢ bond energy (ΔBOND)	0.00	−0.00	0.00	0.00
➢ Angle energy (ΔANGLE)	−0.00	0.00	0.00	0.00
➢ Dihedral energy (ΔDIHED)	−0.00	0.00	−0.00	0.00
➢ Urey–Bradley term (ΔUB)	−0.00	−0.00	−0.00	−0.00
➢ Improper torsion (ΔIMP)	−0.00	0.00	−0.00	0.00
➢ CMAP correction (ΔCMAP)	0.00	−0.00	−0.00	0.00
➢ van der waals energy (ΔVDW)	−168.84	−120.37	−84.01	−92.07
➢ electrostatic energy (ΔEEL)	−1050.55	−702.14	−1063.66	−807.46
➢ 1–4 van der waals (Δ1–4 VDW)	0.00	0.00	−0.00	−0.00
➢ 1–4 electrostatic (Δ1–4 EEL)	0.00	−0.00	−0.00	−0.00
➢ Polar Solvation free energy (ΔEGB)	1153.10	779.88	1091.97	827.58
➢ Non-polar Solvation free energy (ΔESURF)	−26.44	−18.99	−16.67	−16.74
➢ Gas-phase free energy (ΔGGAS)	−1219.39	−822.52	−1147.66	−899.53
➢ Solvation free energy (ΔGSOLV)	1126.66	760.88	1075.30	810.84
➢ **total binding free energy (ΔGTOTAL)**	✓ **–92.73**	✓ **–61.63**	✓ **–72.37**	✓ **–88.69**
➢ **Overall Structural interpretation**	**Binding of UbK48 induces interface perturbation and destabilizes the SKP2-p27 complex**		**Binding of UbK63 enhances interactions and stabilizes the SKP2-Akt1 complex**	

The bold values in table represent the total binding free energy.

In contrast, the SKP2-Akt1 complex shows stable binding (−72.37 kcal/mol), supported by strong electrostatic interactions (ΔEEL = −1063.66 kcal/mol). The interface remains well-defined, as shown in Figure 9B. Following K63-linked ubiquitination, the binding affinity remains favorable (−88.69 kcal/mol). Although electrostatic contributions decrease (to −807.46 kcal/mol), this is compensated by solvation effects. As illustrated in Figure 9B, the interaction interface remains preserved, indicating stabilization of the SKP2-Akt1 complex.

Overall, these results demonstrate that K48-linked ubiquitination induces destabilization of the SKP2-p27 interaction, whereas K63-linked ubiquitination promotes stabilization of the SKP2-Akt1 interaction, consistent with the observed structural arrangements in Figure 9.

## 4 Discussion

In this study, we aimed to understand how SKP2 differentiates between its two major substrates, p27 and Akt1, in the context of K48- and K63-linked ubiquitination. Our structural and computational analyses show that ubiquitin linkage type induces distinct conformational and energetic changes, thereby modulating SKP2-substrate interactions in a linkage-specific manner. This concept fits emerging findings showing how the ubiquitin system controls cancer-related signals in precise, adaptable ways (Ohtake et al., 2016; Tracz and Bialek, 2021; Waltho et al., 2024).

Our model suggests every protein in this process has distinct structural patterns, which influence how it binds. These shapes define contact areas through specific arrangements rather than random forms. Each element follows a consistent layout tied to its role. Their configuration effects partnering by exposing particular regions. Instead of uniform folds, variations support selective binding behavior. These differences help to explain why SKP2 binds p27 and Akt1 through separate conformational routes. Previous studies have noted that SKP2 recognizes substrates through a combination of rigid and flexible regions (Gao et al., 2009; Grice and Nathan, 2016), and our topological mapping supports this paradigm. Specifically, we observed that p27 forms a localized, compact interfacial assembly upon binding, whereas Akt1 engages through a significantly broader contact area that remains stable even under modification. These distinct interfacial architectures provide a structural basis for the contrasting biological fates degradation versus signaling activation of the two substrates.

A comparative analysis of the SKP2-p27 and SKP2-Akt1 interfaces reveals that while both complexes are initially stabilized by a robust network of electrostatic anchors, their energetic trajectories diverge significantly following ubiquitin attachment. Our docking results (Table 3) demonstrate that the SKP2–p27 interface is initially reinforced by critical interactions, including the ASP268-TYR74 hydrogen bond and a prominent salt bridge at ARG344-GLU80 (2.60 Å). However, our binding-energy analysis shows that UbK48 linkage induces a measurable weakening of this association. This energetic destabilization likely facilitates the transition of the polyubiquitinated substrate from the E3 ligase to the 26 S proteasome for degradation, a mechanism consistent with the established structural role of the SKP2 LRR domain in managing proteolytic turnover (Cai et al., 2020; Kamel et al., 2025; Tsvetkov et al., 1999; Zafonte et al., 2000). In contrast, the UbK63-linked Akt1 complex exhibits a different thermodynamic profile, where ubiquitin attachment not only preserves but actively enhances substrate recognition through residues like LYS119 and GLN172. The emergence of new stabilizing contacts, such as the ASP133-LYS135 salt bridge (2.62 Å), suggests that K63-linked chains act as a structural scaffold that stabilizes the kinase in an active conformation. This finding aligns with experimental evidence showing that SKP2-mediated K63-ubiquitination of the Akt1 PH domain is a non-proteolytic event that promotes membrane localization and pathway activation rather than degradation (Chan et al., 2012; Wang et al., 2019). Collectively, these results suggest that the geometric specificity of the isopeptide bond acts as a regulatory switch, where UbK48-linked complexes are primed for dissociation and decay, while UbK63-linked assemblies are stabilized for downstream oncogenic signaling (Cai et al., 2020).

The transition from initial substrate recruitment to the ubiquitinated state represents a critical regulatory juncture in the SKP2 pathway. In the present study, the modeled systems correspond to the post-transfer state, in which ubiquitin is already covalently attached to the substrate. Our comparative analysis of the SKP2–substrate interfaces, both with and without the ubiquitin linkage indicates that the presence of ubiquitin acts as a stabilizing structural anchor that modulates the conformational landscape of the complex.

For the p27 substrate, which is characterized by high initial conformational variability (Global RMSD of 23.7 ± 7.4 Å), incorporation of the K48-linked ubiquitin results in a highly converged, “locked” orientation, as evidenced by an RMSD of 0.3 ± 0.2 Å and an improved HADDOCK score of −120.9 ± 2.1. This structural stabilization is followed by a strategic “structural loosening” during molecular dynamics simulations, where the Radius of Gyration (Rg) increases to 3.57 ± 0.22 nm and intermolecular hydrogen bonds decrease to 10.96 ± 3.94. This behavior suggests enhanced flexibility that may facilitate downstream proteasomal targeting.

Conversely, the SKP2–Akt1 complex maintains a compact assembly upon incorporation of K63-linked ubiquitin (Rg of 3.33 ± 0.04 nm), accompanied by an increased hydrogen-bond network (14.42 ± 3.34), supporting sustained structural stability required for signaling activation.

Collectively, these findings indicate that ubiquitin linkage type differentially modulates the structural and dynamic properties of SKP2–substrate complexes potentially influencing their downstream functional outcomes. In this study, the enzymatic transfer of ubiquitin from the E2 enzyme to the substrate was not explicitly modeled, as such processes require quantum mechanical/molecular mechanical (QM/MM) approaches.

Our computational structural analysis shows that ubiquitination modulates SKP2-substrate interactions through changes in binding energetics and conformational flexibility. For the SKP2-UbK48-p27 complex, K48-linked ubiquitination is associated with reduced binding affinity (−61.63 kcal/mol) and increased structural fluctuations (RMSF 1.23 Å), indicating a more flexible and less stable interaction interface. In contrast, the SKP2-UbK63-Akt1 complex shows stronger binding (−88.69 kcal/mol) and lower fluctuations (RMSF 0.13 Å), reflecting a more stable and well-defined interaction. Overall, these observations indicate that different ubiquitin linkages differentially influence interaction stability and conformational dynamics, thereby modulating SKP2–substrate recognition at the structural level.

The dynamic signatures from RMSD, RMSF, and radius of gyration provide a mechanistic explanation for these outcomes. UbK48 introduces flexibility into the SKP2-p27 assembly, producing an expanded and unstable conformation. Such destabilization reflects the early steps of proteasomal targeting a process previously described in cancer-associated turnover of p27 (Ravaioli et al., 2008). On the other hand, UbK63 maintains a compact and stable SKP2-Akt1 arrangement that favors sustained signaling. This behavior reinforces earlier reports that K63 ubiquitination acts as a scaffold-building mechanism during kinase activation (Chan et al., 2012; Shao et al., 2023).

PCA and FEL analyses provide further insight by showing that UbK48 drives SKP2 into a single highly ordered minimum, which is characteristic of degradation-ready complexes. In contrast, UbK63 generates a deep but broad basin that supports structural coherence without forcing collapse into a rigid state. These energy landscapes match experimental descriptions of conformational control through linkage-specific ubiquitin chains (Feng et al., 2024; Kelso et al., 2021).

Finally, our MM/PBSA results (Table 6) provide supportive evidence consistent with these structural observations. A relative shift in interaction energy is observed upon ubiquitination, where the addition of UbK48 is associated with a less favorable interaction profile for the SKP2–p27 complex (from −92.73 to −61.63 kcal/mol), while UbK63 shows a comparatively more favorable interaction profile for the SKP2–Akt1 system (from −72.37 to −88.69 kcal/mol).

These trends are consistent with experimental studies reporting distinct roles of K48- and K63-linked ubiquitination, where K48 linkage is associated with reduced interaction stability, whereas K63 linkage is associated with more stable interaction profiles (Genheden and Ryde, 2015; Weng et al., 2019; Zhu et al., 2022).

A critical consideration in our structural modeling was the use of a monoubiquitin unit to represent the ubiquitination event. While *in vivo* signaling typically involves the assembly of polyubiquitin chains, the structural ‘decision’ to initiate K48 or K63 linkages is primarily dictated by the geometric orientation of the first isopeptide bond relative to the SKP2 binding cleft (Komander and Rape, 2012; Pickart and Eddins, 2004). By focusing on this 1:1 substrate-ubiquitin initiation stage, we were able to delineate the atomistic transitions required for linkage commitment without the excessive conformational entropy and structural ‘noise’ associated with longer, disordered polyubiquitin chains (Faggiano et al., 2016). Crucially, our structural analysis confirms that the target lysine residues (Lys48 and Lys63) remain highly solvent-exposed and oriented away from the primary SKP2-substrate interface. This outward-facing orientation ensures these sites are sterically accessible for the recruitment of additional ubiquitin molecules, potentially facilitating the assembly of polyubiquitin chains or the formation of K48/K63 branched chains, which have recently been shown to convert K63-linked “seeds” into proteasomal degradation signals (Hehl et al., 2023; Ohtake et al., 2018)**.** Although the inclusion of a longer chain would likely enhance the overall binding avidity through secondary distal contacts, it is anticipated that the core interfacial determinants identified in our simulations such as the stabilizing salt bridges in the SKP2-UbK63-Akt1 complex are established at this monoubiquitination stage (Husnjak and Dikic, 2012; Komander and Rape, 2012; Lemma et al., 2023).

Our findings reveal that SKP2 utilizes a “conformational sorting” mechanism to distinguish between degradation-bound and activation-bound substrates. This discrimination is achieved through substrate-specific binding signatures at the atomic level: p27 engages SKP2 through a flexible, polar “induced-fit” interface, while Akt1 is captured through a rigid, electrostatic-driven “ionic lock.” These unique recognition modes directly influence the downstream ubiquitination machinery. The flexibility of the p27 complex facilitates an expanded, solvent-exposed conformation (SASA = 428.90 nm^2^) that spatially favors K48-linked polyubiquitination, the hallmark of proteasomal breakdown. Conversely, the high structural stiffness of the Akt1 complex (RMSF = 0.09 Å) maintains a compact, signaling-competent architecture optimized for K63-linked ubiquitin attachment. The high degree of structural convergence between our physics-based docking and independent AlphaFold 3 predictions (RMSD < 1.2 Å) confirms that these divergent pathways are driven by structurally robust and biologically relevant binding modes.

## 5 Conclusion

In conclusion, this study provides an atomistic-level structural framework for understanding SKP2-mediated dual ubiquitination, a key regulatory axis in breast cancer progression. By integrating high-resolution molecular docking, AlphaFold 3 validation, and extensive 200 ns molecular dynamics simulations, we characterize how SKP2 engages distinct substrates as well as their interactive association with different structural outcomes.

Our findings suggest that the molecular recognition of p27 and Akt1 is associated with distinct structural behaviors that influence isopeptide-linked conformations. The flexible, hydrogen-bond-driven recognition of p27 corresponds to increased structural loosening consistent with K48-linked degradation, whereas the more rigid, salt-bridge-driven recognition of Akt1 is associated with a compact and stable assembly consistent with K63-linked signaling.

From a translational perspective, identifying these linkage-specific conformational features extends beyond static models and supports the concept of a dynamic SKP2 interaction interface with potential therapeutic relevance. The interfacial interactions and salt-bridge networks observed in the SKP2-substrate models provide a structural framework for the rational design of small-molecule inhibitors. Such approaches may enable selective modulation of oncogenic Akt1 signaling or stabilization of p27 levels, thereby offering a potential strategy to address SKP2-driven drug resistance. Overall, this computational framework connects molecular-level insights with therapeutic exploration and provides a testable basis for the development of linkage-selective intervention strategies in aggressive breast cancer.

## Data Availability

The original contributions presented in the study are included in the article/Supplementary Material, further inquiries can be directed to the corresponding author.
