# Supplementary material for: Molecular dynamics simulations for understanding dual ubiquitination mechanisms to consider S-phase kinase-associated protein 2 (SKP2) as a potential drug target in breast cancer
Source: Front Chem. 2026 May 29;14:1786015. doi: 10.3389/fchem.2026.1786015 (PMC13260631; doi:10.3389/fchem.2026.1786015)
Supplement: Supplementary file 1 [file DataSheet1.docx]

**Supplementary Table 1: Total Docking clusters for the complex SKP2-p27**

| **Complex** | **Cluster** | **HADDOCK Score (Mean ± SD)** | **RMSD (Å) (Mean ± SD)** | **Z-score** | **VdW (Mean ± SD)** | **Electrostatic (Mean ± SD)** | **Desolvation (Mean ± SD)** | **Restraint Energy (Mean ± SD)** | **BSA (Å²) (Mean ± SD)** |
| --- | --- | --- | --- | --- | --- | --- | --- | --- | --- |
| SKP2–p27 | Cluster 2 | -103.8 +/- 7.8 | 23.7± 7.4 | -2.0 | -51.1 ± 6.3 | -192.1 ± 37.9 | -23.1 +/- 7.1 | 89.1 +/- 61.8 | 1838.3 +/- 200.2 |
| SKP2–p27 | Cluster 6 | -88.9 ± 6.6 | 28.3 ± 0.5 | -0.9 | -37.8 ± 8.0 | -279.8 ± 81.6 | -5.5 ± 6.7 | 103.5 ± 43.0 | 1604.3 ± 62.6 |
| SKP2–p27 | Cluster 3 | -87.8 ± 8.0 | 30.4 ± 0.7 | -0.8 | -36.8 ± 6.5 | -330.6 ± 20.9 | -3.2 ± 2.8 | 183.3 ± 41.8 | 1666.1 ± 59.8 |
| SKP2–p27 | Cluster 1 | -84.7 ± 2.9 | 27.5 ± 1.4 | -0.4 | -48.5 ± 4.0 | -78.4 ± 23.6 | -31.4 ± 6.7 | 108.9 ± 36.0 | 1361.6 ± 152.8 |
| SKP2–p27 | Cluster 5 | -84.0 ± 17.6 | 25.5 ± 0.8 | -0.3 | -33.0 ± 8.3 | -196.3 ± 43.3 | -30.5 ± 1.9 | 187.3 ± 60.5 | 1589.2 ± 265.8 |
| SKP2–p27 | Cluster 7 | -80.4 ± 7.1 | 19.3 ± 1.5 | 0.1 | -26.3 ± 6.1 | -272.0 ± 29.3 | -10.4 ± 2.9 | 107.5 ± 53.4 | 1387.5 ± 138.9 |
| SKP2–p27 | Cluster 14 | -79.1 ± 14.0 | 6.9 ± 2.7 | 0.3 | -30.0 ± 9.0 | -246.2 ± 108.2 | -7.1 ± 7.2 | 71.9 ± 42.7 | 1445.3 ± 55.0 |
| SKP2–p27 | Cluster 11 | -75.0 ± 12.5 | 23.7 ± 1.8 | 0.8 | -28.1 ± 7.3 | -164.9 ± 47.3 | -25.4 ± 3.8 | 114.8 ± 79.5 | 1125.9 ± 318.1 |
| SKP2–p27 | Cluster 13 | -70.3 ± 4.7 | 18.1 ± 0.4 | 1.3 | -36.3 ± 5.8 | -187.9 ± 32.5 | -8.5 ± 4.9 | 120.2 ± 54.7 | 1458.6 ± 33.1 |
| SKP2–p27 | Cluster 12 | -67.3 ± 18.9 | 27.2 ± 0.2 | 1.7 | -45.2 ± 9.7 | -61.3 ± 22.4 | -27.1 ± 7.3 | 173.5 ± 51.4 | 1443.9 ± 247.8 |


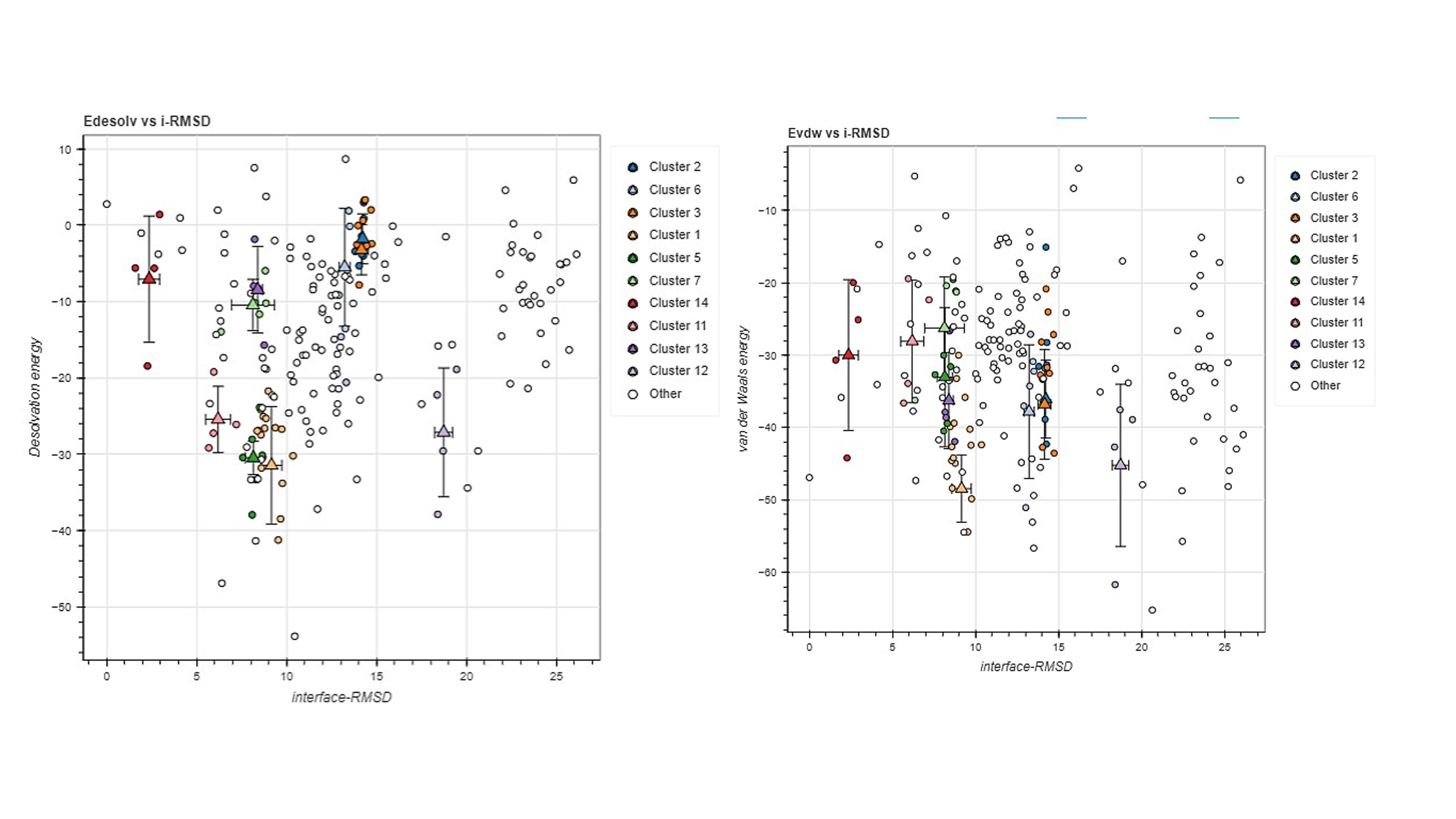

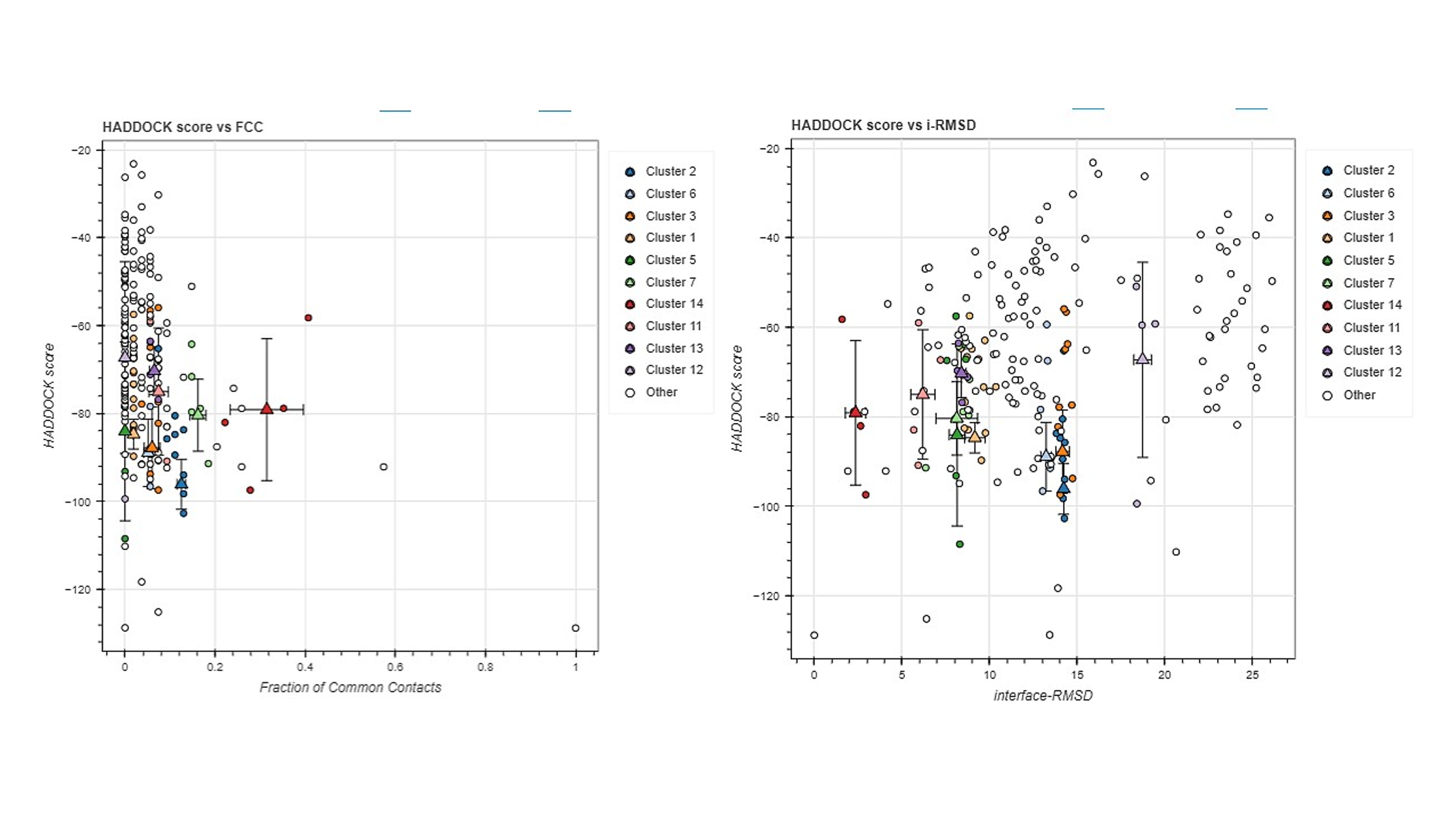
**Model Analysis**

**
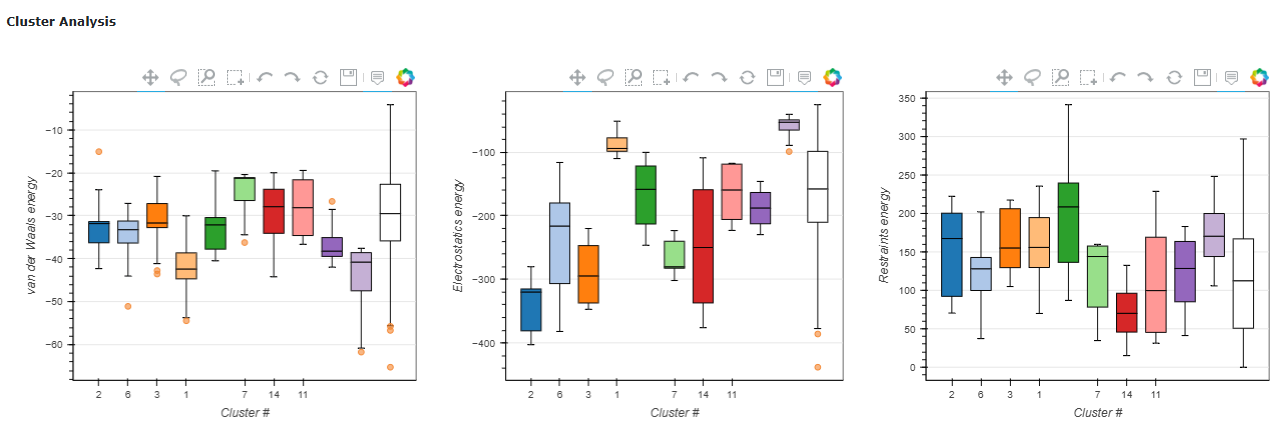
**
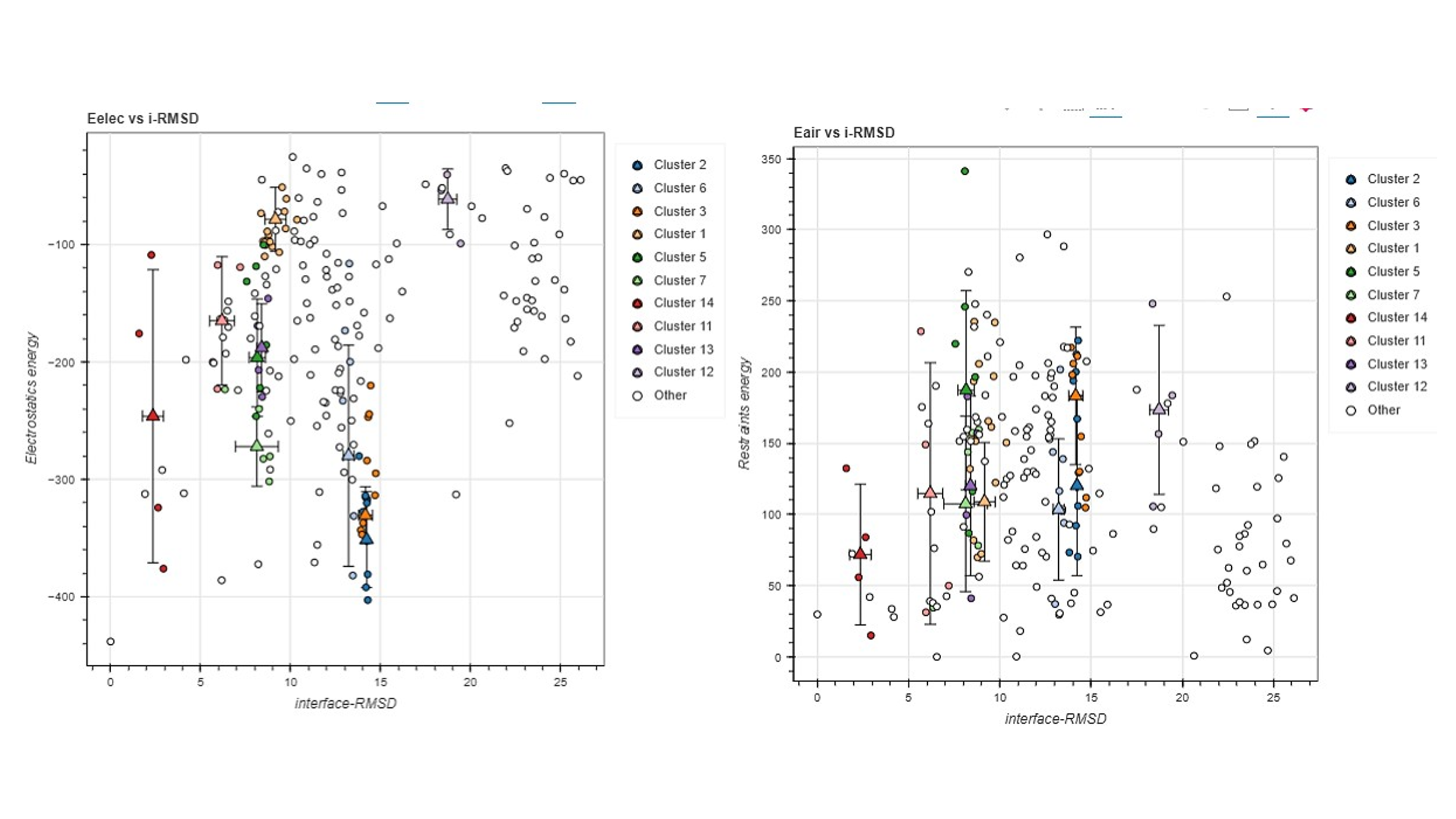


| **Complex** | **Cluster** | **HADDOCK Score (Mean ± SD)** | **RMSD (Å) (Mean ± SD)** | **Z-score** | **VdW (Mean ± SD)** | **Electrostatic (Mean ± SD)** | **Desolvation (Mean ± SD)** | **Restraint Energy (Mean ± SD)** | **BSA (Å²) (Mean ± SD)** |
| --- | --- | --- | --- | --- | --- | --- | --- | --- | --- |
| SKP2–UbK48–p27 | Cluster 1 | -120.9 +/- 2.1 | 0.3 +/- 0.2 | -1.9 | -62.6 +/- 2.1 | -168.3 +/- 34.7 | -23.1 +/- 7.1 | 79.9 +/- 19.9 | 1838.3 +/- 200.2 |
| SKP2–UbK48–p27 | Cluster 2 | -69.1 ± 4.2 | 27.3 ± 0.3 | -0.5 | -17.5 ± 4.7 | -302.1 ± 50.8 | 5.0 ± 4.4 | 38.6 ± 5.1 | 1358.9 ± 55.5 |
| SKP2–UbK48–p27 | Cluster 4 | -66.8 ± 5.7 | 2.8 ± 0.4 | -0.3 | -36.1 ± 4.5 | -91.5 ± 17.3 | -17.6 ± 3.7 | 52.6 ± 19.2 | 1099.8 ± 108.3 |
| SKP2–UbK48–p27 | Cluster 3 | -63.1 ± 3.8 | 16.2 ± 0.4 | 0.0 | -24.1 ± 5.6 | -213.7 ± 39.0 | -1.5 ± 1.7 | 52.9 ± 55.1 | 1131.6 ± 166.2 |
| SKP2–UbK48–p27 | Cluster 6 | -62.4 ± 12.8 | 22.4 ± 0.3 | 0.0 | -23.4 ± 3.7 | -241.7 ± 72.5 | 6.7 ± 1.4 | 26.4 ± 15.1 | 1093.2 ± 102.7 |
| SKP2–UbK48–p27 | Cluster 7 | -53.7 ± 15.6 | 9.9 ± 1.4 | 0.7 | -24.6 ± 8.9 | -148.1 ± 40.4 | -6.2 ± 9.8 | 66.9 ± 55.1 | 928.9 ± 112.9 |
| SKP2–UbK48–p27 | Cluster 5 | -51.1 ± 1.7 | 18.7 ± 1.0 | 0.9 | -17.3 ± 3.3 | -172.4 ± 23.1 | -0.6 ± 3.6 | 12.0 ± 18.9 | 820.1 ± 67.7 |
| SKP2–UbK48–p27 | Cluster 8 | -44.9 ± 17.3 | 24.1 ± 0.5 | 1.3 | -17.9 ± 7.6 | -168.5 ± 40.9 | 5.3 ± 3.4 | 14.0 ± 14.0 | 827.0 ± 180.4 |

**Supplementary Table 2: Total Docking clusters for the complex SKP2-UbK48-p27**


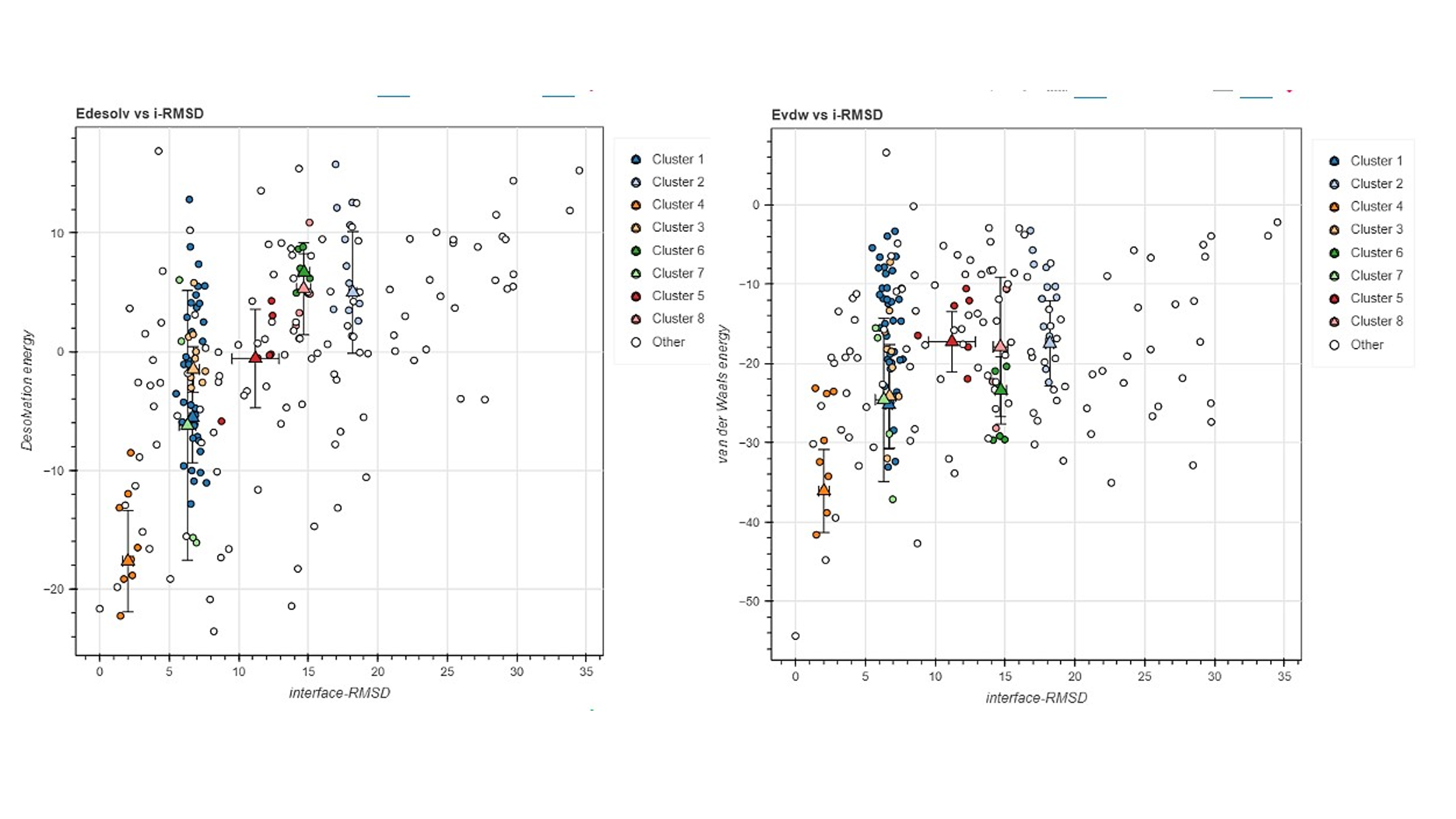

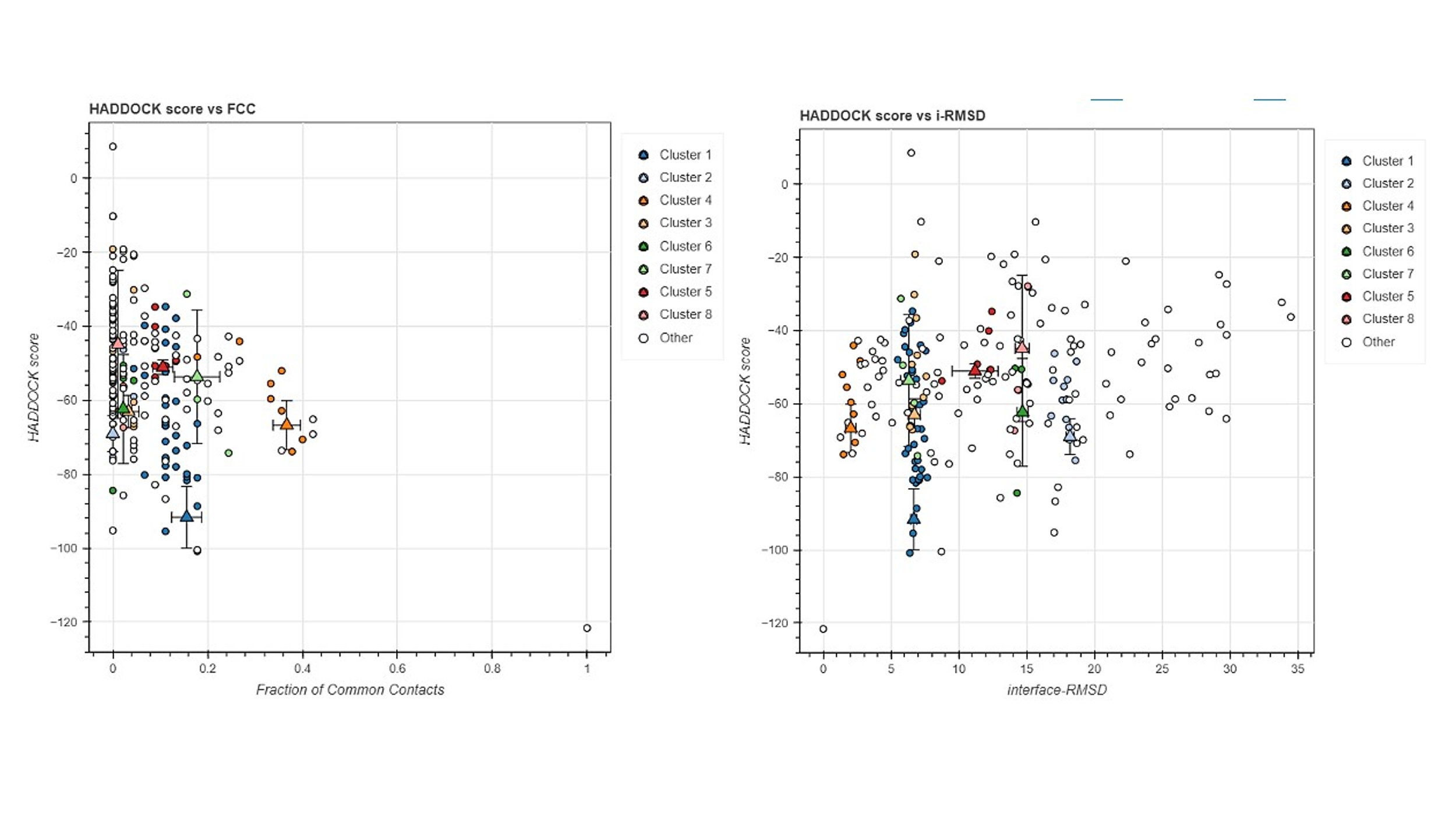
**Model Analysis**

**
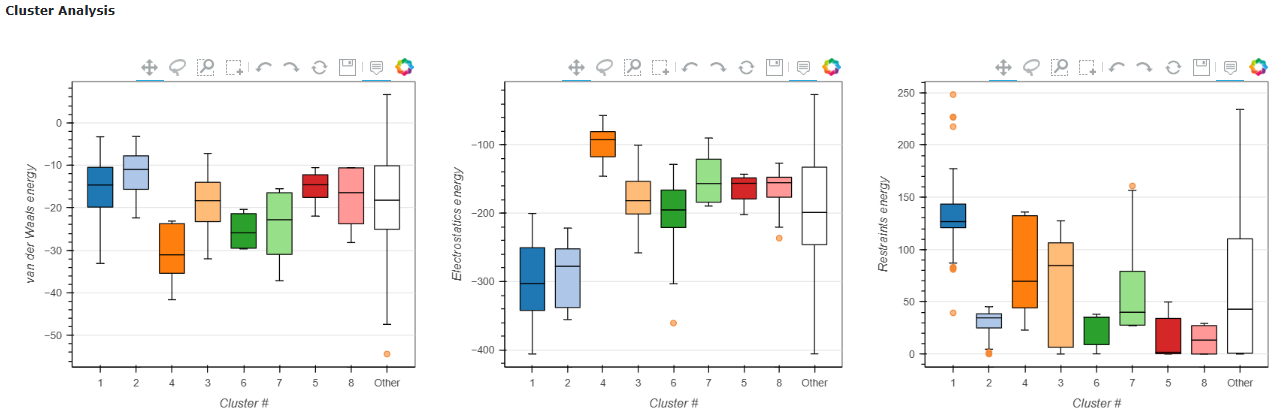
**
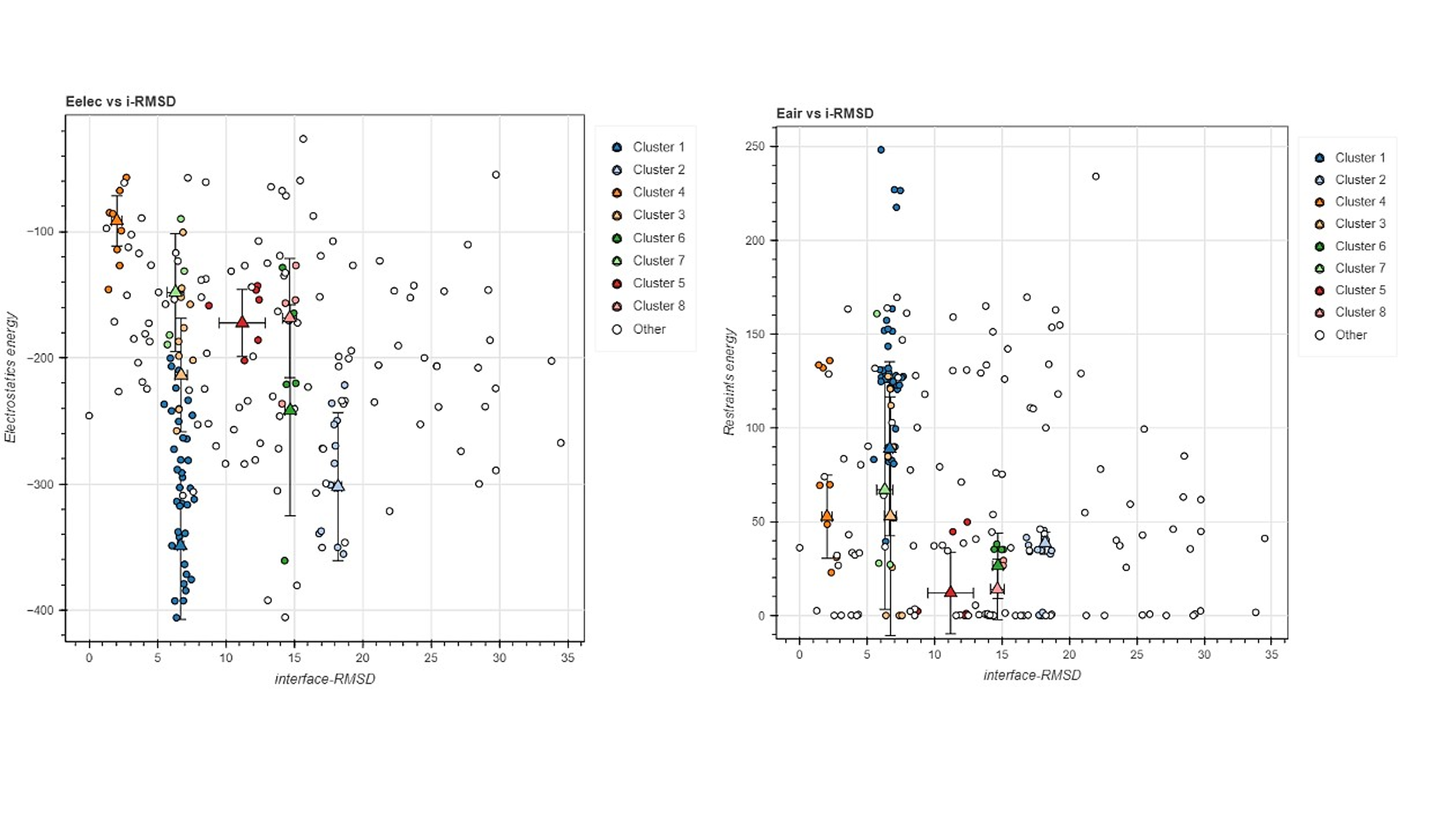


**Supplementary Table 3: Total Docking clusters for the complex SKP2-Akt1**

| **Complex** | **Cluster** | **HADDOCK Score (Mean ± SD)** | **RMSD (Å) (Mean ± SD)** | **Z-score** | **VdW (Mean ± SD)** | **Electrostatic (Mean ± SD)** | **Desolvation (Mean ± SD)** | **Restraint Energy (Mean ± SD)** | **BSA (Å²) (Mean ± SD)** |
| --- | --- | --- | --- | --- | --- | --- | --- | --- | --- |
| SKP2–Akt1 | Cluster 1 | -85.5 ± 8.7 | 8.4 ± 0.2 | -1.3 | -42.0 ± 3.4 | -323.6 ± 32.9 | 2.2 ± 4.6 | 190.2 ± 54.3 | 1964.0 ± 101.3 |
| SKP2–Akt1 | Cluster 2 | -92.1 ± 1.2 | 6.1 ± 0.8 | -1.2 | -32.5 ± 3.1 | -255.8 ± 32.1 | -11.2 ± 2.4 | 27.2 ± 13.6 | 1434.2 ± 44.2 |
| SKP2–Akt1 | Cluster 3 | -78.7 ± 3.4 | 19.3 ± 0.2 | -0.4 | -40.2 ± 8.1 | -169.8 ± 44.6 | -16.6 ± 4.0 | 120.1 ± 51.9 | 1437.7 ± 160.8 |
| SKP2–Akt1 | Cluster 4 | -57.1 ± 8.6 | 12.7 ± 0.4 | 0.8 | -32.0 ± 5.5 | -99.0 ± 26.2 | -13.6 ± 3.6 | 82.8 ± 54.1 | 1256.4 ± 248.2 |
| SKP2–Akt1 | Cluster 5 | -70.2 ± 19.2 | 19.9 ± 0.4 | 0.0 | -31.1 ± 2.6 | -148.3 ± 92.5 | -12.8 ± 4.1 | 33.0 ± 1.4 | 1245.0 ± 227.3 |
| SKP2–Akt1 | Cluster 6 | -44.4 ± 4.6 | 6.0 ± 0.3 | 1.5 | -18.3 ± 6.0 | -94.7 ± 15.1 | -8.3 ± 2.1 | 11.2 ± 15.2 | 797.4 ± 98.1 |
| SKP2–Akt1 | Cluster 7 | -56.9 ± 9.2 | 5.1 ± 0.5 | 0.8 | -32.5 ± 3.2 | -136.2 ± 31.2 | -12.1 ± 1.3 | 149.1 ± 11.5 | 1272.4 ± 148.0 |


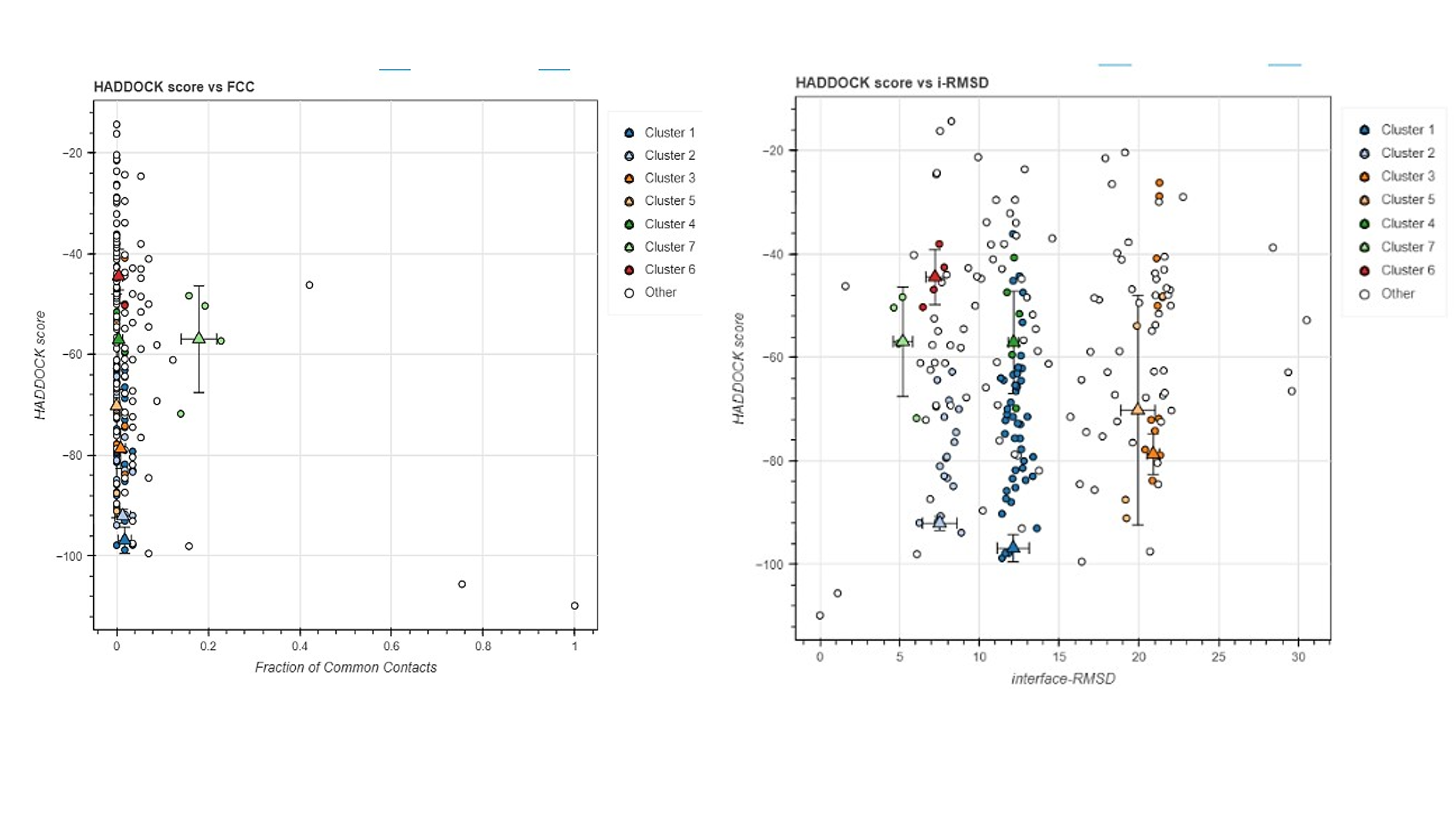

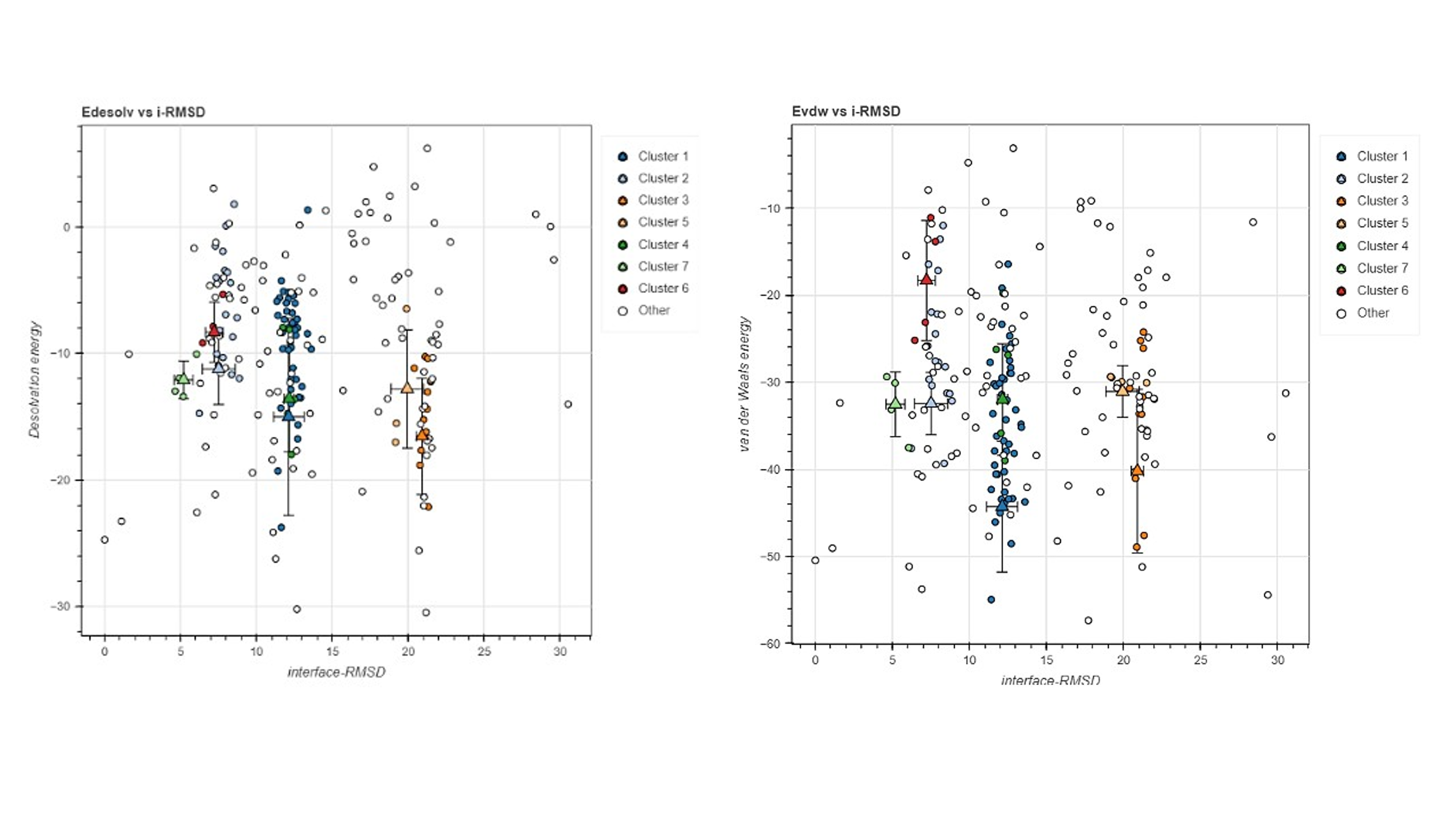
**Model Analysis**

**
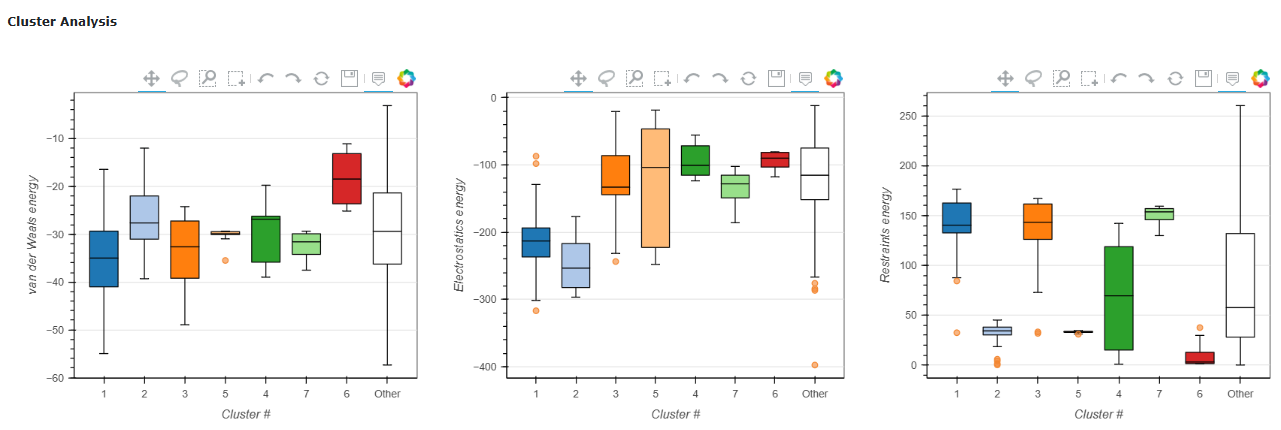
**
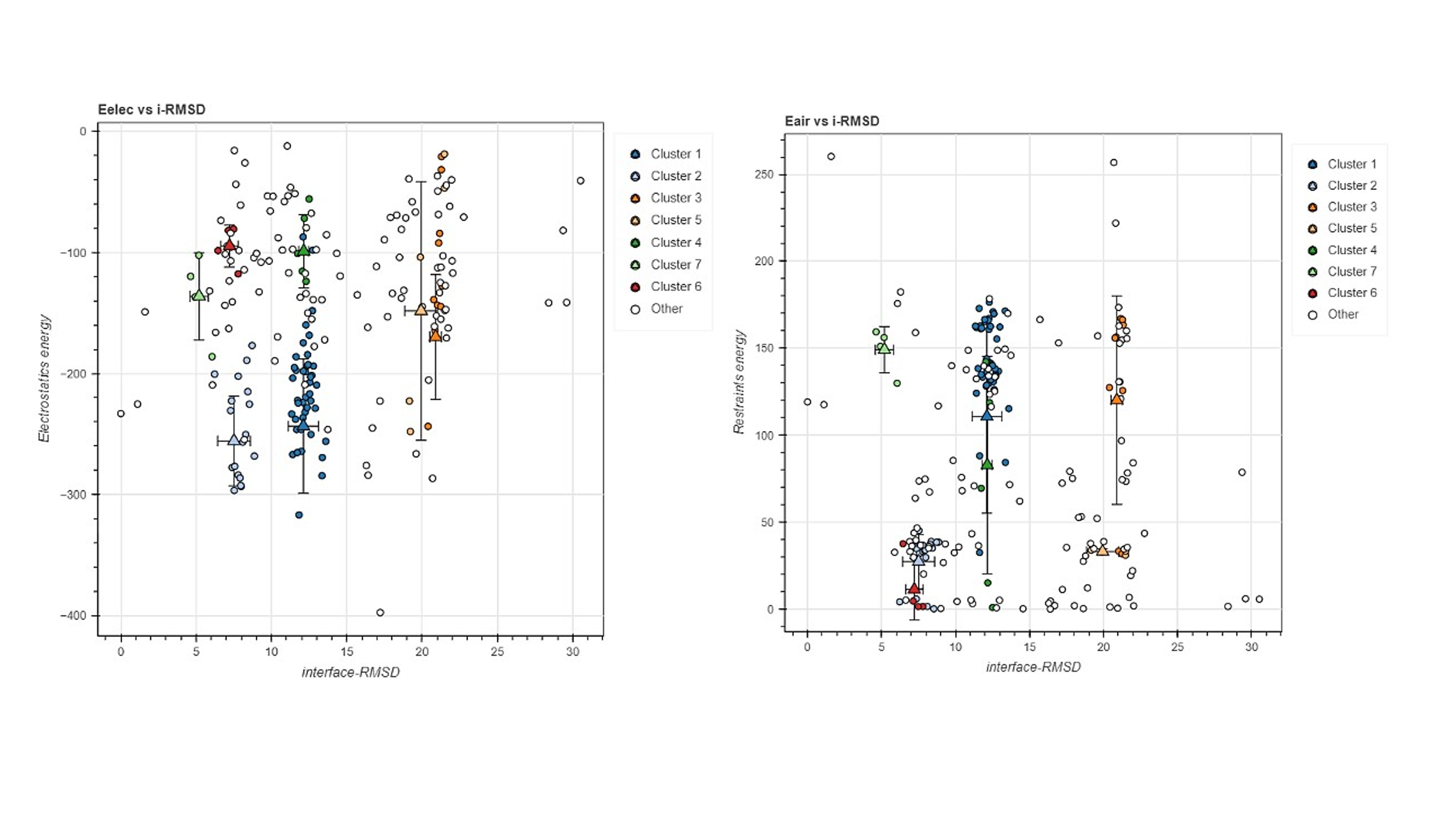


| **Complex** | **Cluster** | **HADDOCK Score (Mean ± SD)** | **RMSD (Å) (Mean ± SD)** | **Z-score** | **VdW**  **(Mean ± SD)** | **Electrostatic (Mean ± SD)** | **Desolvation (Mean ± SD)** | **Restraint Energy (Mean ± SD)** | **BSA (Å²) (Mean ± SD)** |
| --- | --- | --- | --- | --- | --- | --- | --- | --- | --- |
| SKP2–UbK63–Akt1 | Cluster 1 | -78.1 ± 0.8 | 7.2 +/- 0.0 | -1.2 | -39.7 ± 1.0 | -338.1 ± 8.4 | 14.3 ± 1.3 | 149.5 ± 22.3 | 1895.7 ± 27.5 |
| SKP2–UbK63–Akt1 | Cluster 2 | -109.2 ± 3.5 | 18.6 ± 0.3 | -0.4 | -65.1 ± 4.3 | -140.8 ± 8.6 | -35.1 ± 3.5 | 192.2 ± 38.7 | 2015.4 ± 34.3 |
| SKP2–UbK63–Akt1 | Cluster 3 | -116.2 ± 2.6 | 13.0 ± 0.5 | -0.7 | -52.4 ± 2.6 | -252.2 ± 12.9 | -20.4 ± 2.0 | 69.8 ± 55.9 | 1917.4 ± 43.8 |
| SKP2–UbK63–Akt1 | Cluster 4 | -92.1 ± 7.6 | 23.4 ± 0.5 | 0.3 | -46.1 ± 6.5 | -172.5 ± 27.1 | -25.5 ± 3.7 | 141.0 ± 54.0 | 2057.9 ± 151.0 |
| SKP2–UbK63–Akt1 | Cluster 5 | -92.4 ± 16.6 | 16.1 ± 0.7 | 0.3 | -60.4 ± 6.8 | -86.1 ± 47.4 | -31.9 ± 2.9 | 171.5 ± 47.0 | 1900.1 ± 131.2 |
| SKP2–UbK63–Akt1 | Cluster 6 | -73.4 ± 10.2 | 25.2 ± 0.4 | 1.1 | -34.0 ± 8.9 | -161.1 ± 10.2 | -21.4 ± 2.7 | 143.1 ± 31.9 | 1610.9 ± 163.2 |
| SKP2–UbK63–Akt1 | Cluster 7 | -136.3 ± 8.1 | 3.5 ± 0.4 | -1.1 | -54.7 ± 9.4 | -276.4 ± 35.7 | -46.7 ± 3.2 | 204.1 ± 69.2 | 2420.5 ± 205.9 |
| SKP2–UbK63–Akt1 | Cluster 8 | -88.0 ± 1.9 | 5.0 ± 0.3 | 0.5 | -40.9 ± 3.7 | -225.5 ± 8.6 | -15.8 ± 4.3 | 137.3 ± 48.5 | 1753.7 ± 101.0 |
| SKP2–UbK63–Akt1 | Cluster 9 | -78.2 ± 6.8 | 26.0 ± 0.1 | 0.9 | -39.7 ± 11.7 | -141.8 ± 28.9 | -28.7 ± 2.6 | 185.9 ± 51.5 | 1556.5 ± 156.0 |
| SKP2–UbK63–Akt1 | Cluster 10 | -70.3 ± 5.5 | 1.7 ± 0.5 | 1.2 | -35.3 ± 10.0 | -165.7 ± 39.1 | -14.9 ± 3.9 | 131.1 ± 69.5 | 1577.5 ± 148.5 |

**Supplementary Table 4: Total Docking clusters for the complex SKP2-UbK63-Akt1**


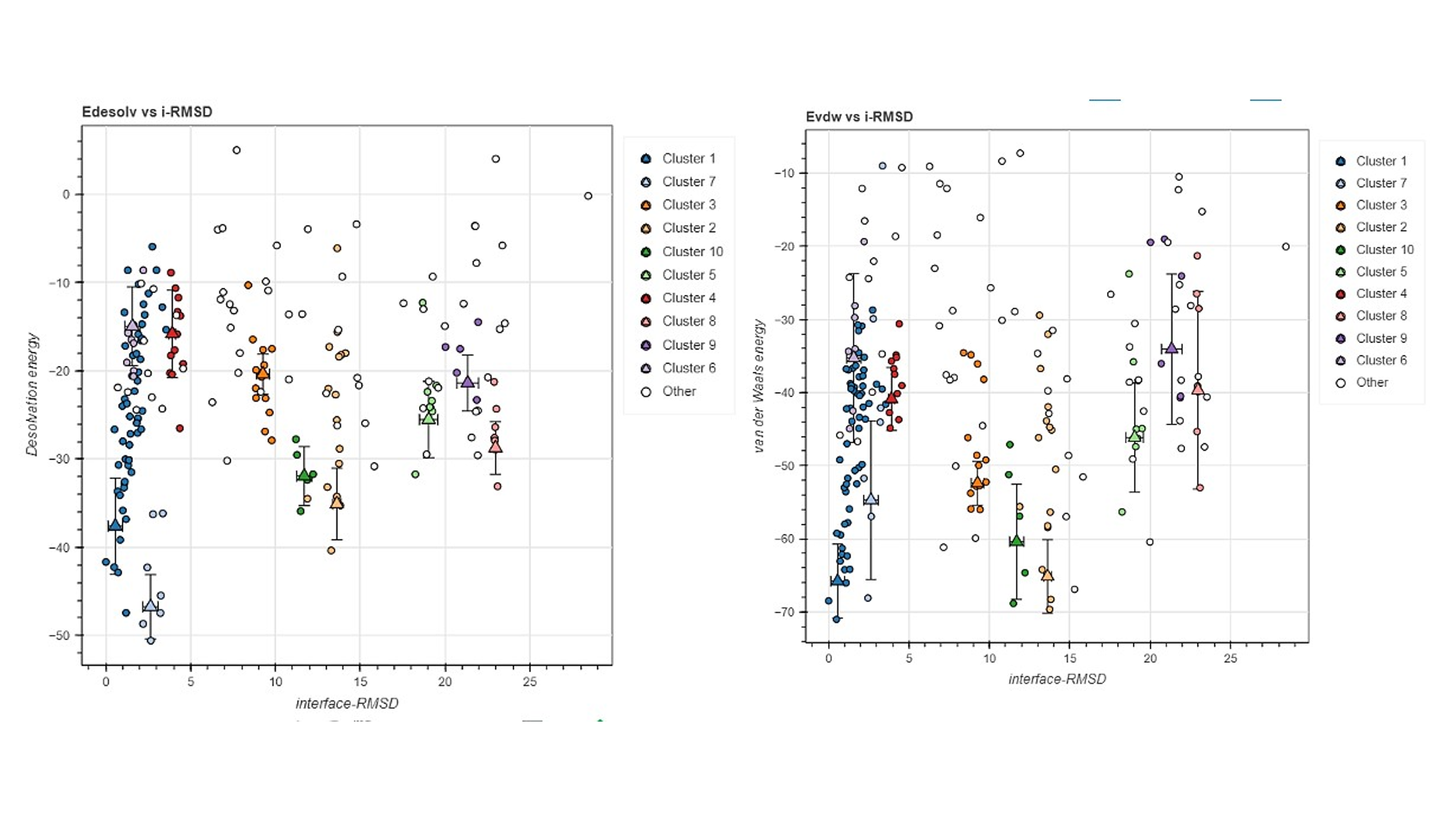

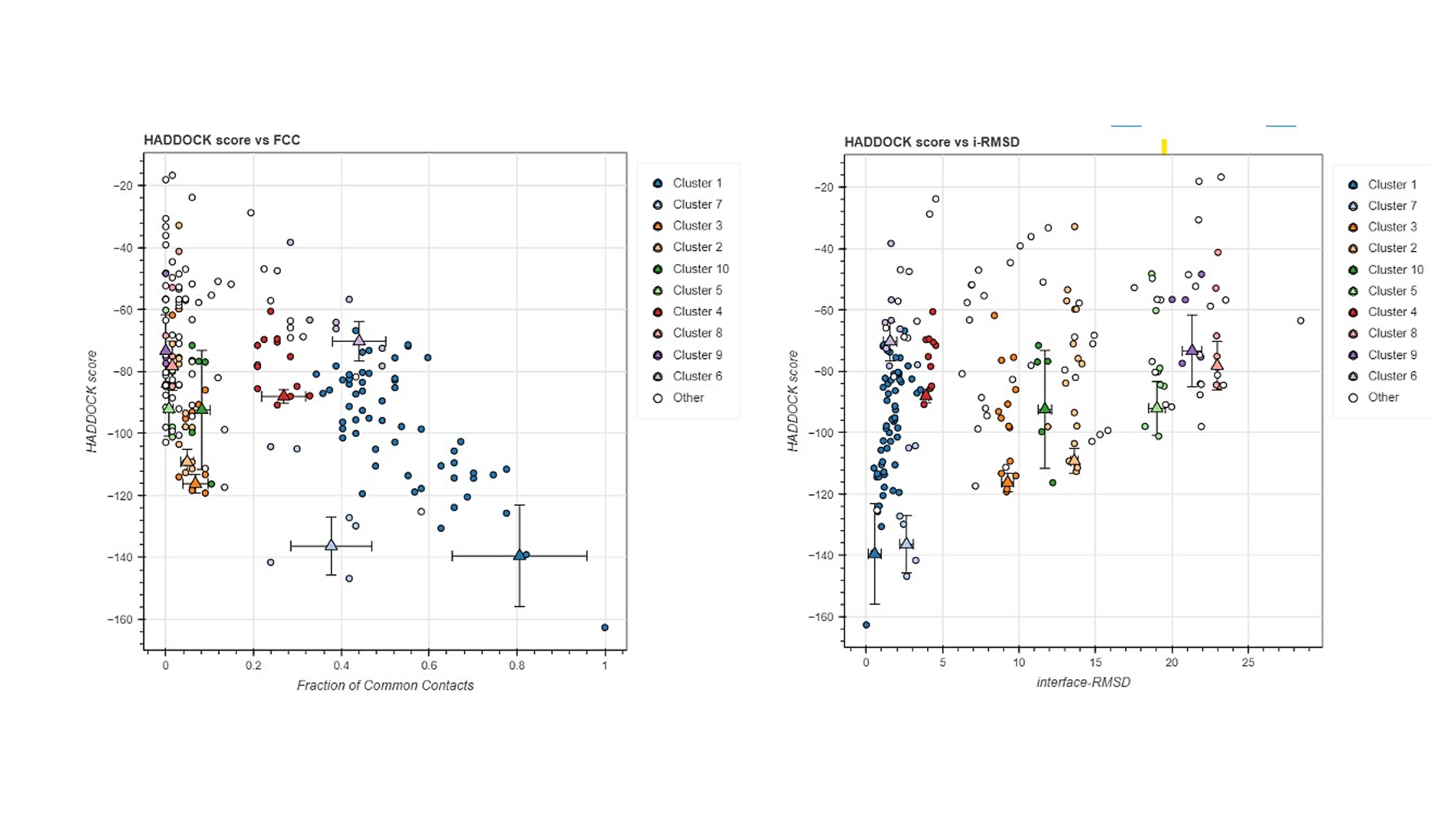
**Model Analysis**


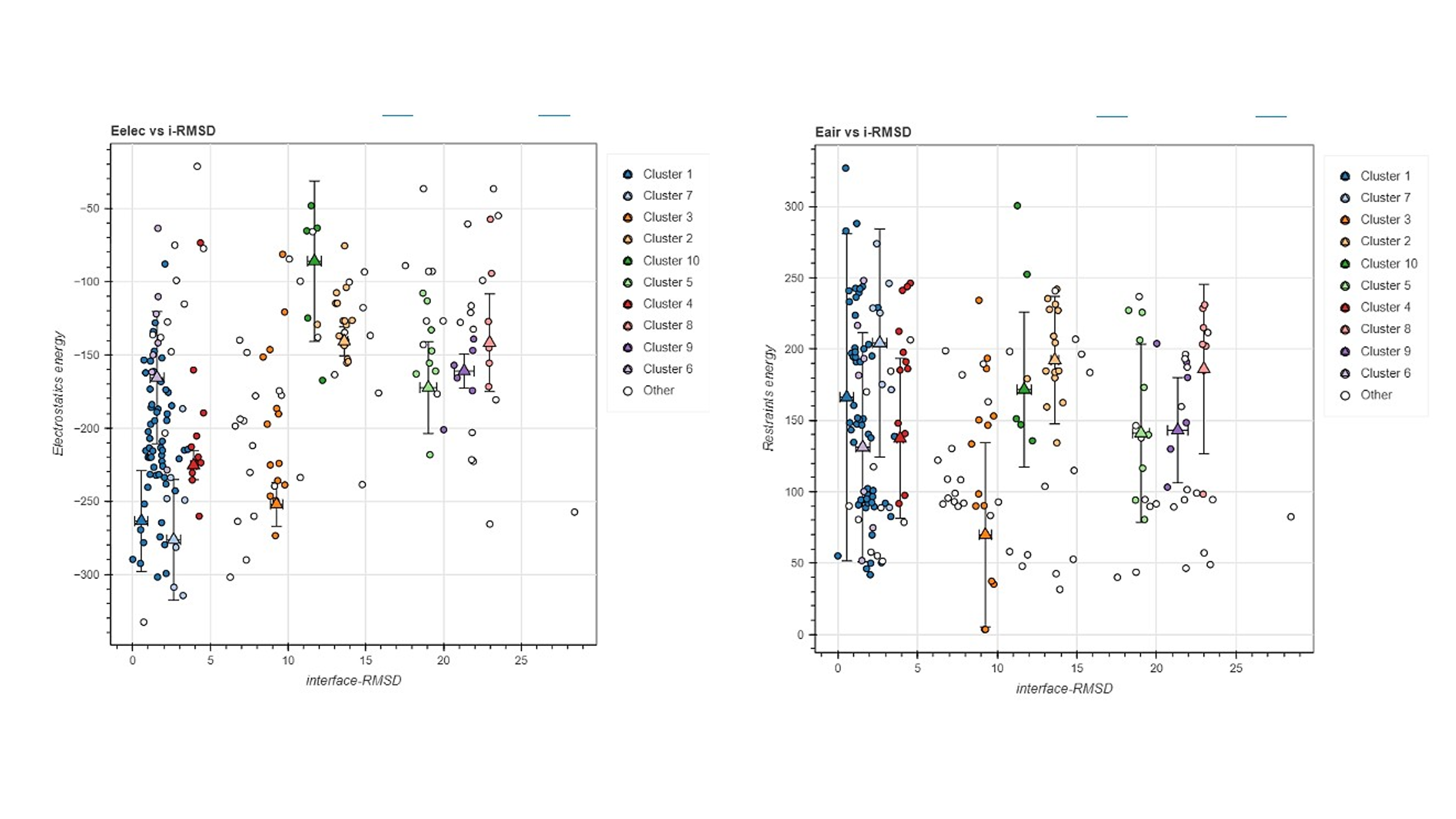


**
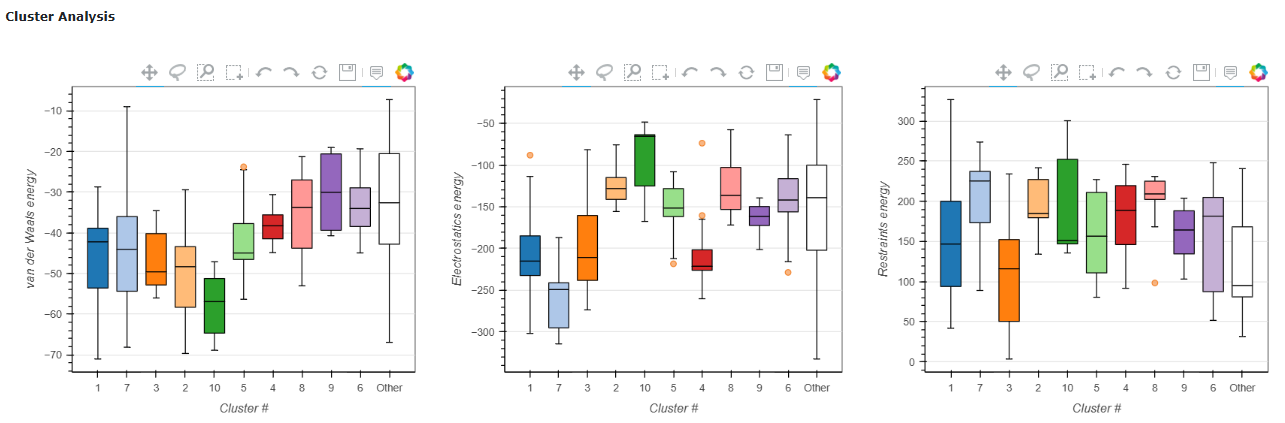
**

**
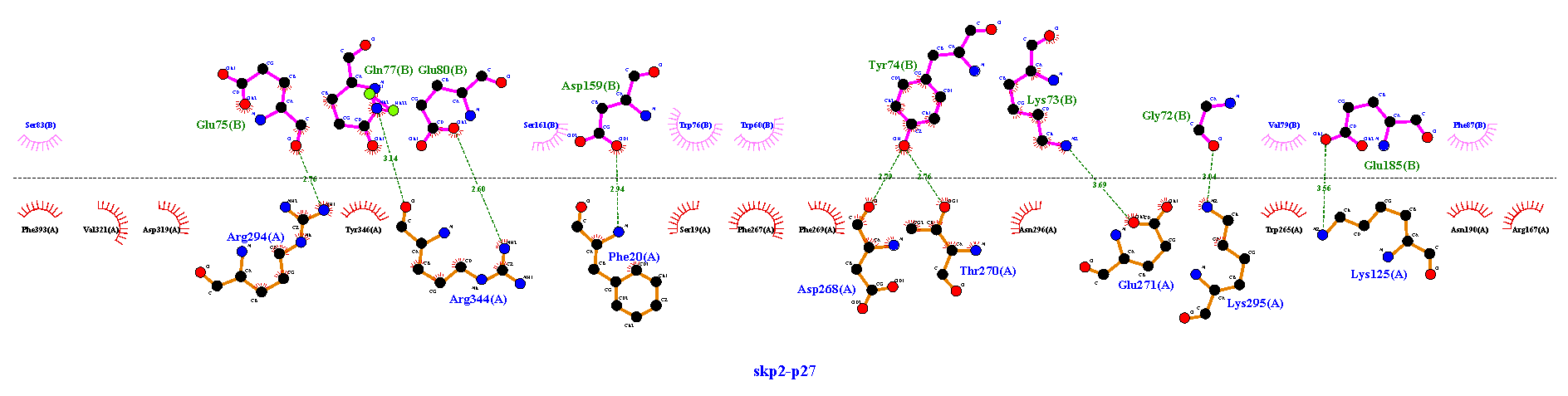

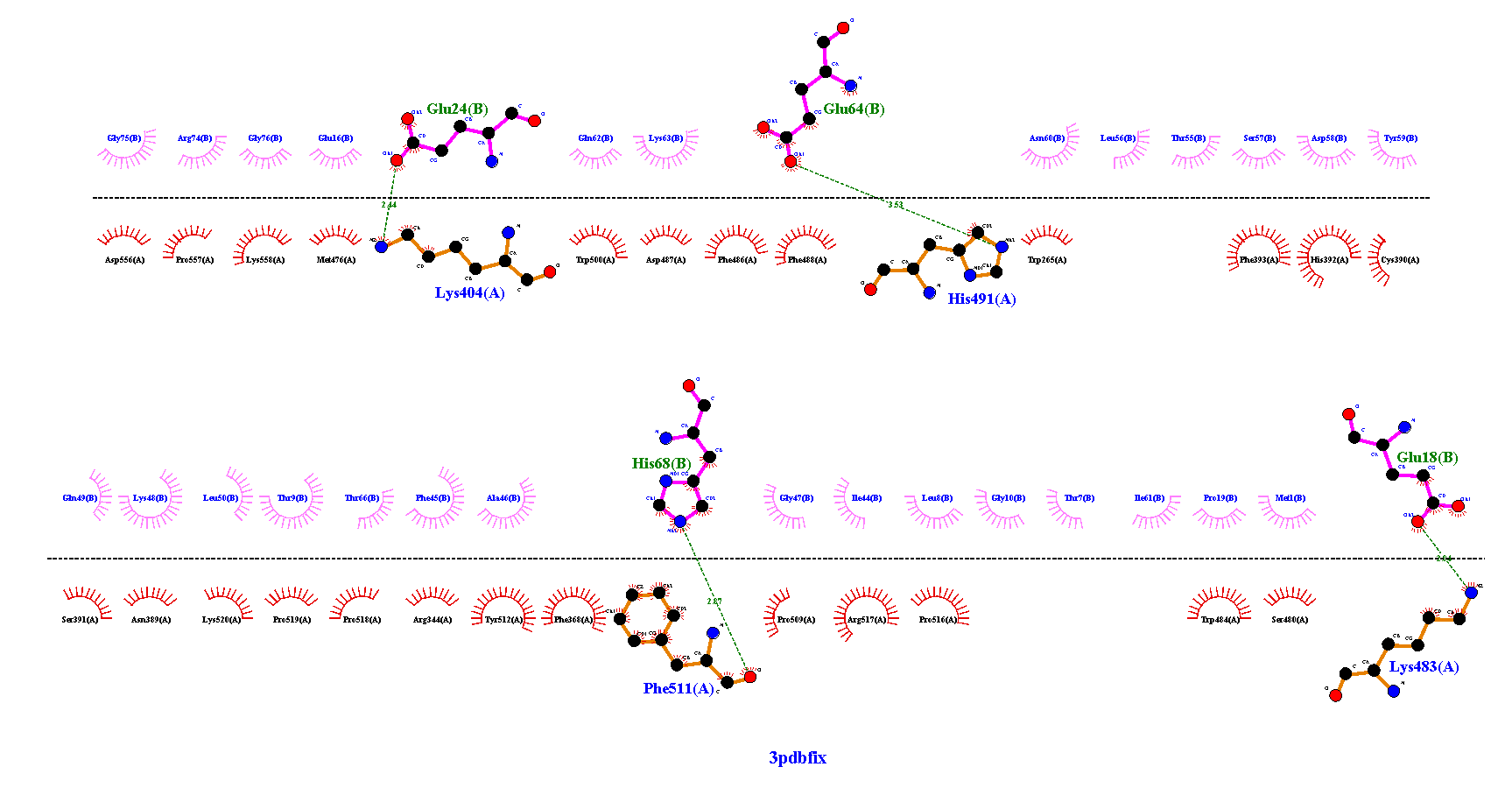

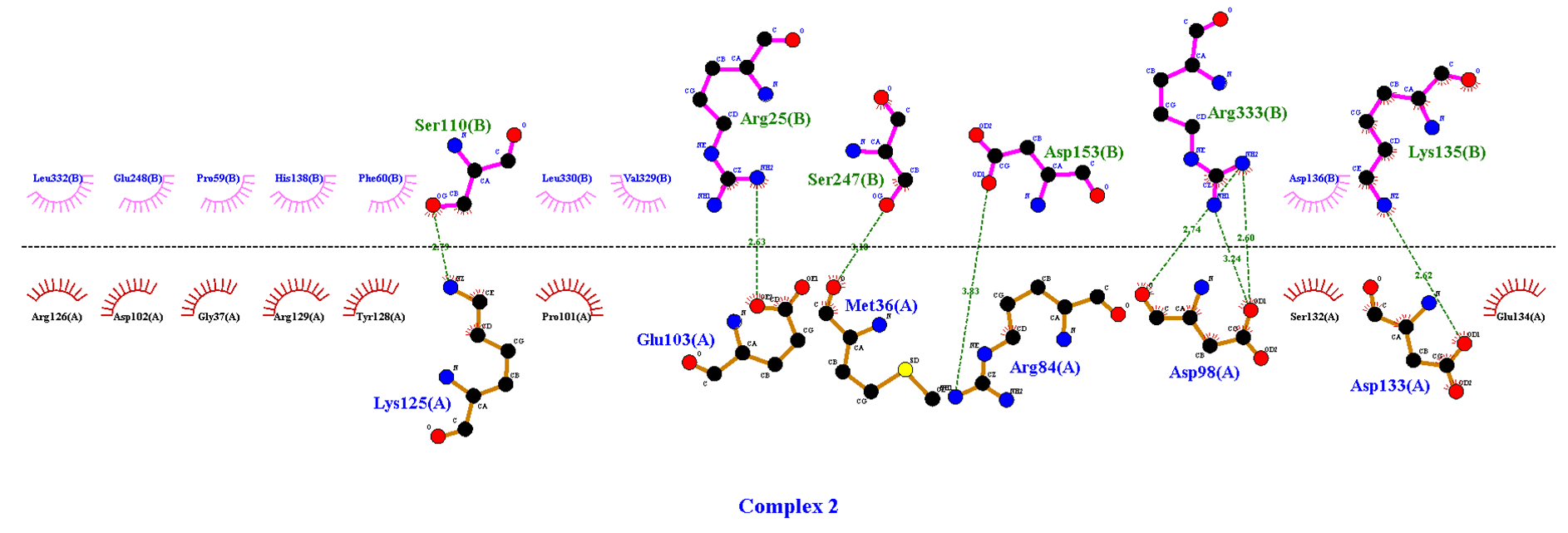

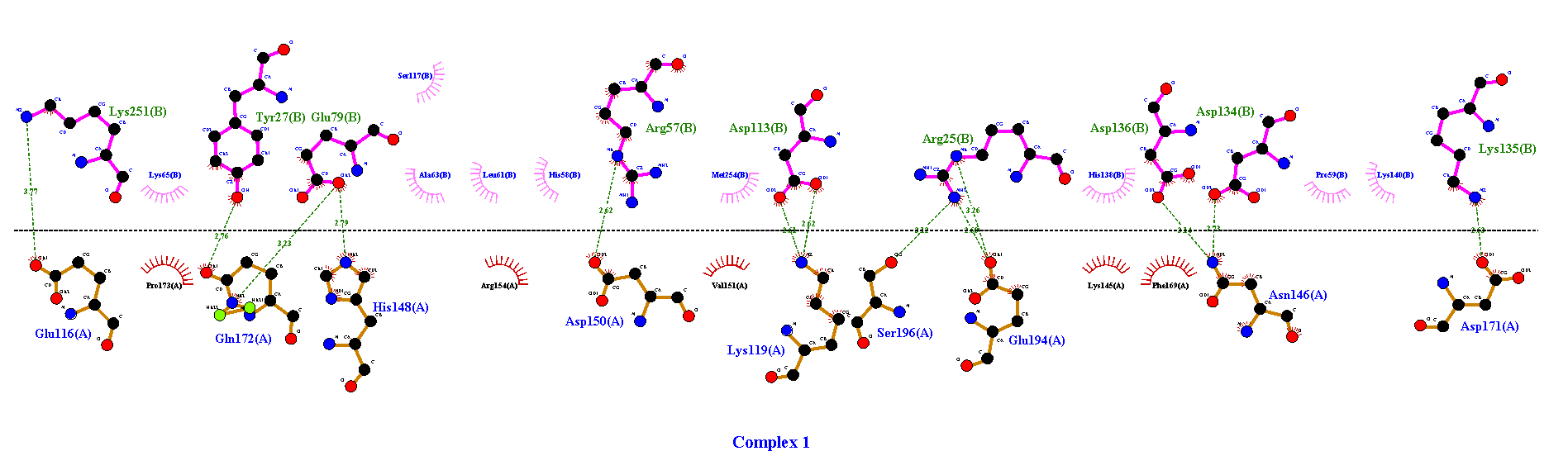
Supplementary Figure 1: Interaction interface analysis**

SKP2+UbK48+ p27

SKP2+UbK63+Akt1

SKP2+p27

SKP2+Akt1

| **Complex** | **Snap-Duration** | **Residue 1 (Chain A)** | **Residue 2 (Chain B)** | **Interaction Type** | **Closest Distance (Å)** |
| --- | --- | --- | --- | --- | --- |
| **SKP2–p27** | **50 ns** | GLU63 | ARG194 | Hydrogen bond | 3.608 |
|  |  | LYS420 | ASP139 | Hydrogen bond | 2.751 |
|  |  | LYS420 | ASP136 | Hydrogen bond | 2.720 |
|  |  | ASP319 | TRP76 | Hydrogen bond | 2.743 |
|  |  | ARG294 | TYR74 | Hydrogen bond | 2.845 |
|  |  | ARG294 | GLU75 | Hydrogen bond | 2.640 |
|  |  | ARG167 | SER83 | Hydrogen bond | 2.657 |
|  |  | LYS119 | SER178 | Hydrogen bond | 2.840 |
|  |  | LYS119 | ASP136 | Hydrogen bond | 2.669 |
|  |  | ARG84 | GLU185 | Hydrogen bond | 2.663 |
|  |  | ARG84 | GLU185 | Hydrogen bond | 2.805 |
|  |  | LYS71 | ASP158 | Hydrogen bond | 2.689 |
|  |  | ARG67 | PRO155 | Hydrogen bond | 2.792 |
|  |  | ARG67 | ALA156 | Hydrogen bond | 2.840 |
|  |  | HIS61 | ALA21 | Hydrogen bond | 2.759 |
|  |  | ARG15 | GLU49 | Hydrogen bond | 2.829 |
|  |  | GLU49 | GLU14 | Hydrogen bond | 2.761 |
|  |  | LYS153 | GLU45 | Hydrogen bond | 2.680 |
|  |  | ARG166 | GLU42 | Hydrogen bond | 2.774 |
|  |  | THR157 | GLU42 | Hydrogen bond | 2.902 |
|  |  | THR157 | GLU42 | Hydrogen bond | 2.851 |
|  |  | GLU42 | SER160 | Hydrogen bond | 2.864 |
|  |  | SER39 | GLN163 | Hydrogen bond | 2.740 |
| **SKP2–p27** | **100 ns** | GLU358 | ARG5 | Hydrogen bond | 3.508 |
|  |  | GLU355 | MET1 | Hydrogen bond | 2.666 |
|  |  | GLU194 | LYS59 | Hydrogen bond | 2.833 |
|  |  | GLU176 | LYS134 | Hydrogen bond | 2.811 |
|  |  | ASP150 | LYS134 | Hydrogen bond | 2.675 |
|  |  | LYS119 | SER178 | Hydrogen bond | 2.865 |
|  |  | LYS119 | ASP136 | Hydrogen bond | 2.629 |
|  |  | ASP76 | ARG195 | Hydrogen bond | 2.845 |
|  |  | ASP76 | ARG195 | Hydrogen bond | 3.044 |
|  |  | PHE20 | ASP158 | Hydrogen bond | 3.126 |
|  |  | GLU45 | LYS153 | Hydrogen bond | 2.701 |
|  |  | GLU42 | LYS190 | Hydrogen bond | 2.715 |
|  |  | GLU42 | THR157 | Hydrogen bond | 2.501 |
|  |  | GLU42 | THR157 | Hydrogen bond | 2.779 |
|  |  | MET36 | LYS189 | Hydrogen bond | 2.838 |
|  |  | TRP24 | SER160 | Hydrogen bond | 3.269 |
|  |  | THR21 | ASN164 | Hydrogen bond | 2.674 |
| **SKP2–p27** | **150 ns** | GLU358 | ARG5 | Hydrogen bond | 3.349 |
|  |  | GLU194 | LYS59 | Hydrogen bond | 2.947 |
|  |  | GLU358 | ASN3 | Hydrogen bond | 2.671 |
|  |  | GLU358 | SER2 | Hydrogen bond | 2.752 |
|  |  | GLU358 | MET1 | Hydrogen bond | 2.744 |
|  |  | GLU355 | MET1 | Hydrogen bond | 2.561 |
|  |  | LYS119 | ASP136 | Hydrogen bond | 2.629 |
|  |  | PHE20 | ASP159 | Hydrogen bond | 2.588 |
|  |  | GLU45 | LYS153 | Hydrogen bond | 2.633 |
|  |  | PHE20 | GLY177 | Hydrogen bond | 2.806 |
|  |  | GLU358 | SER2 | Hydrogen bond | 2.778 |
| **SKP2–p27** | **200 ns** | GLU194 | LYS59 | Hydrogen bond | 2.567 |
|  |  | LYS119 | ASP136 | Hydrogen bond | 2.716 |
|  |  | GLU358 | SER2 | Hydrogen bond | 2.647 |
|  |  | GLU358 | SER2 | Hydrogen bond | 2.637 |
|  |  | GLU358 | MET1 | Hydrogen bond | 2.562 |
|  |  | GLU355 | MET1 | Hydrogen bond | 2.683 |
|  |  | SER238 | LYS81 | Hydrogen bond | 3.055 |
|  |  | GLU271 | LY73 | Hydrogen bond | 2.754 |
|  |  | GLY60 | ARG196 | Hydrogen bond | 3.171 |
|  |  | ASN58 | CYS29 | Hydrogen bond | 2.826 |
|  |  | ASP47 | LYS153 | Hydrogen bond | 2.737 |
|  |  | GLY37 | VAL184 | Hydrogen bond | 2.984 |

**Supplementary Table 5:** Time evolution of key intermolecular interactions observed during the MD trajectory (50, 100, 150, and 200 ns snapshots) for the SKP2–p27 complex.

**
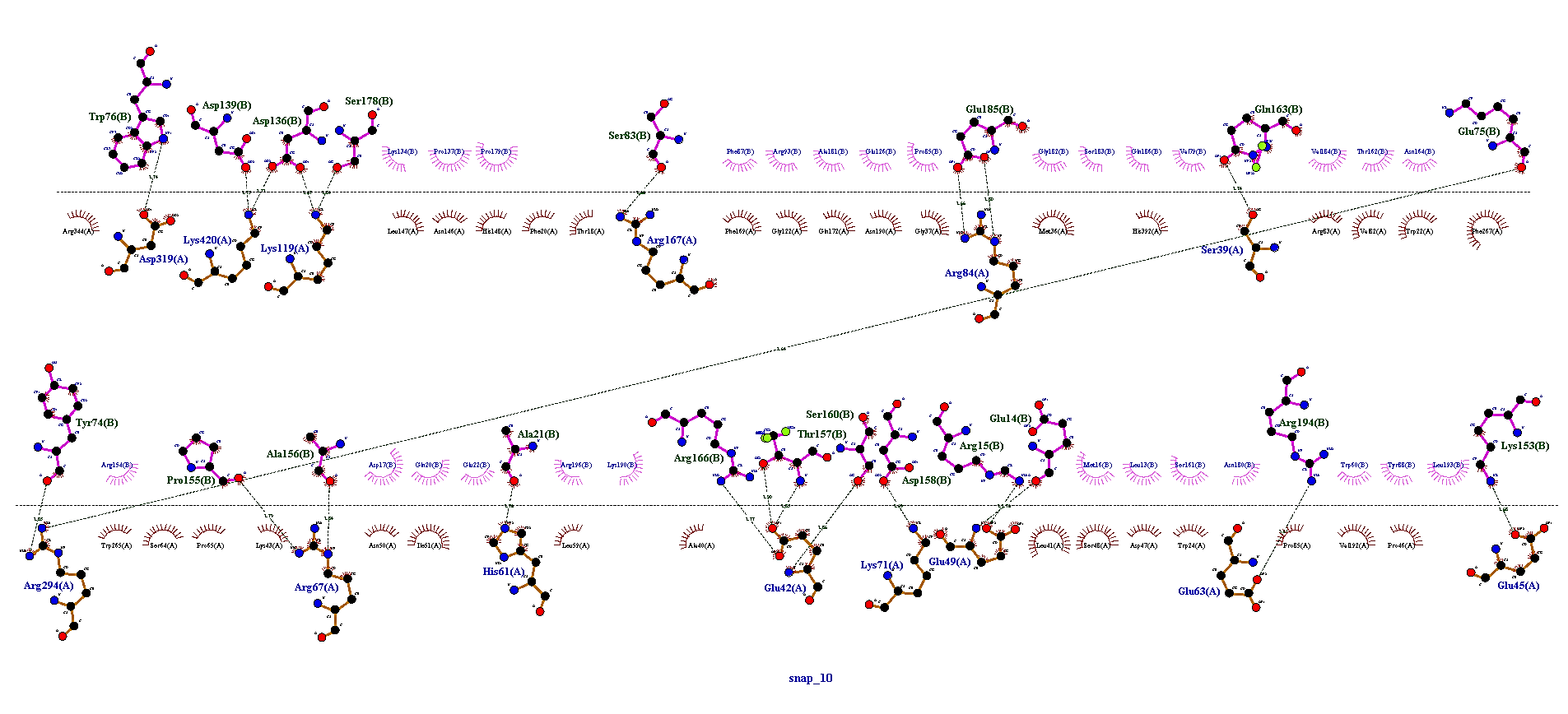
At 50 ns**

**
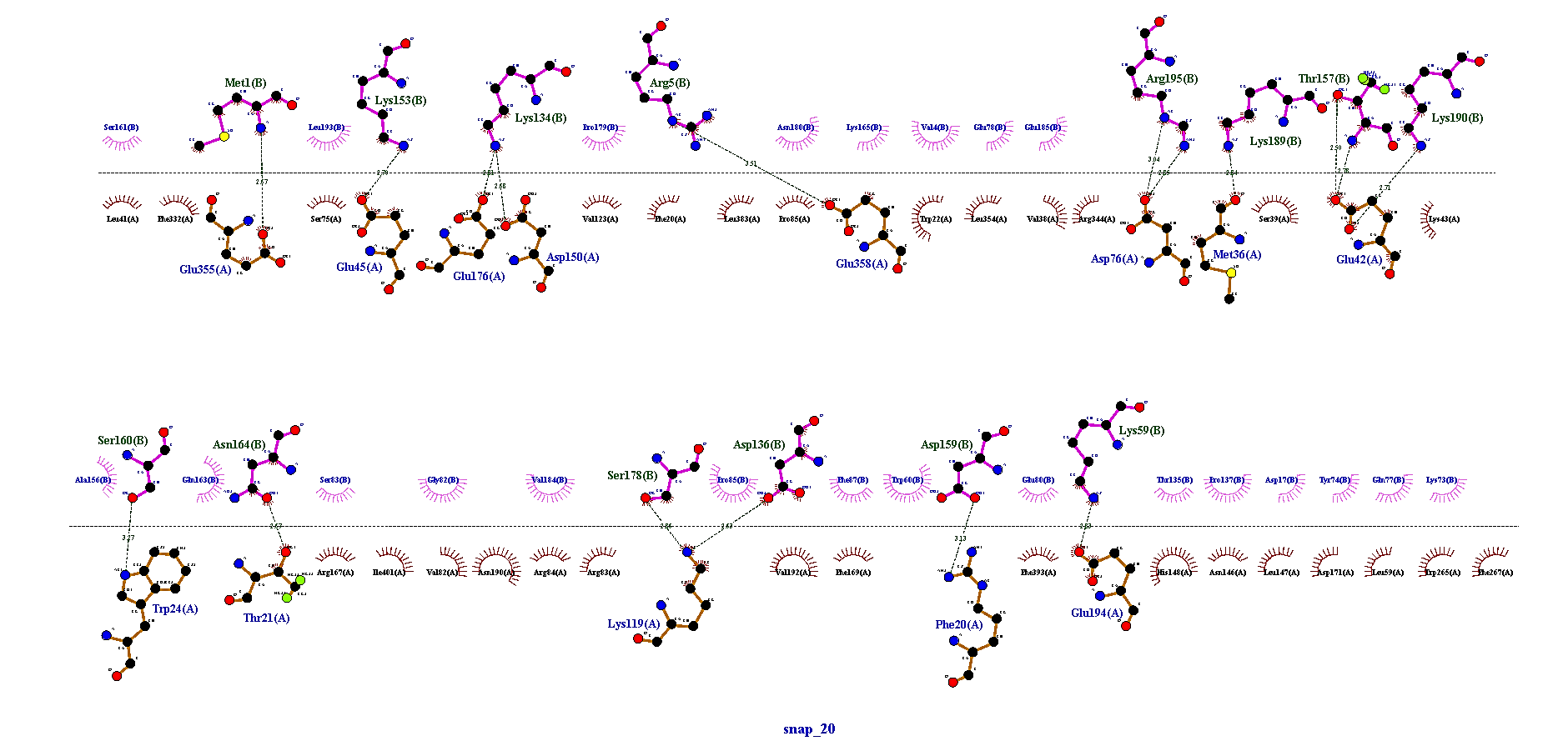
At 100 ns**

**
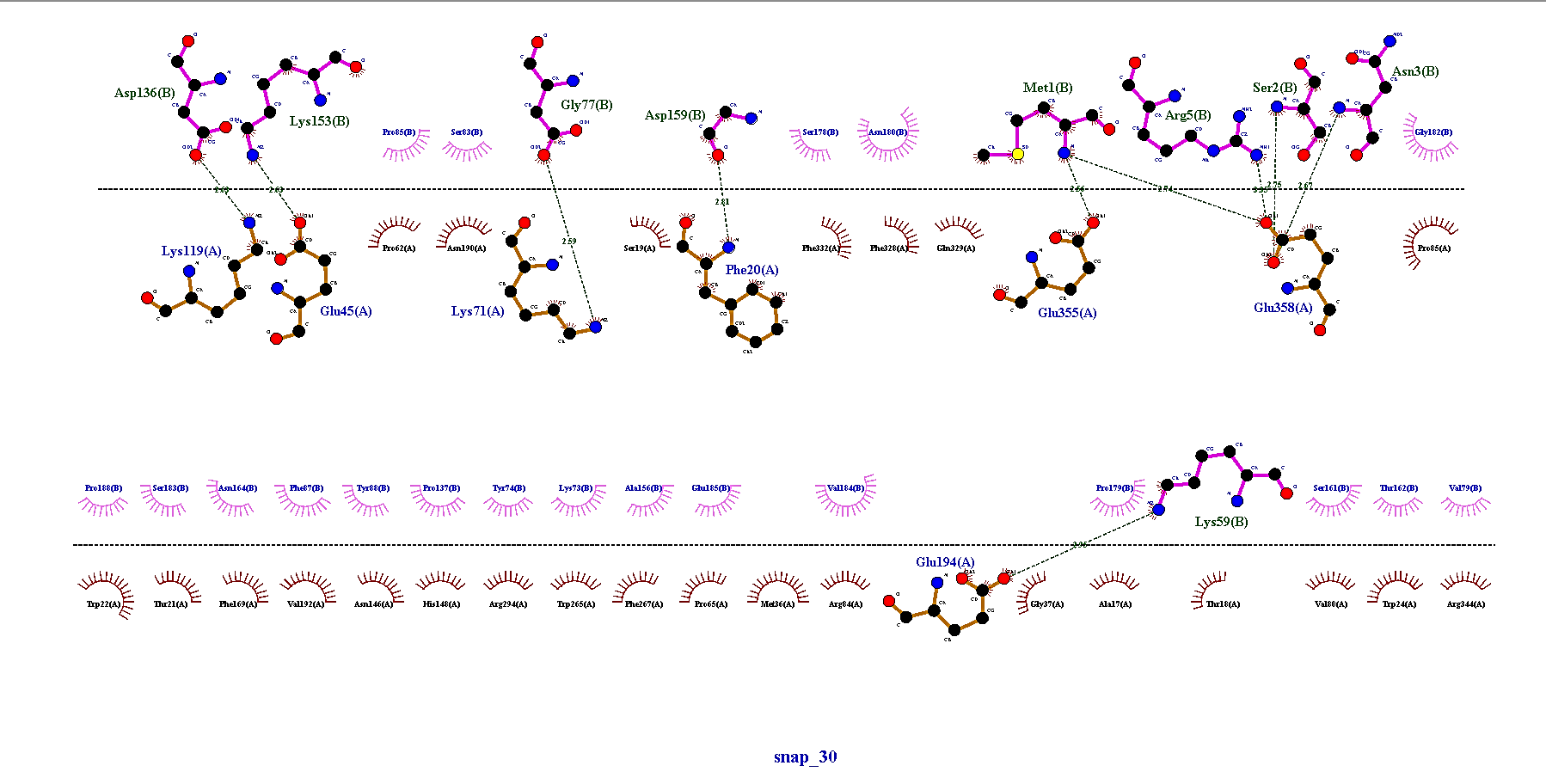
At 150 ns**

**
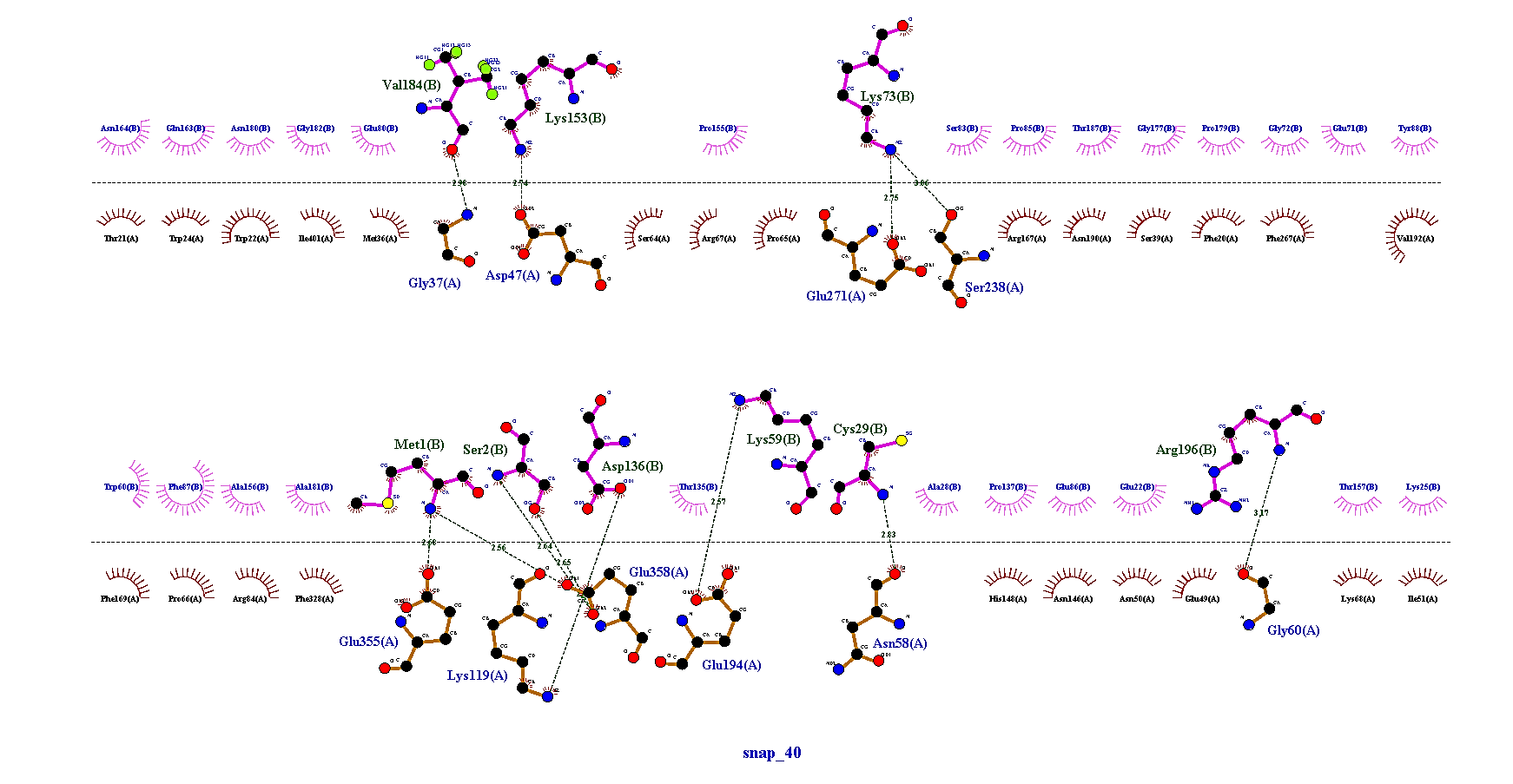
At 200 ns**

| **Complex** | **Snap-Duration** | **Residue 1 (Chain A)** | **Residue 2 (Chain B)** | **Interaction Type** | **Closest Distance (Å)** |
| --- | --- | --- | --- | --- | --- |
| SKP2–UbK48–p27 | 50 ns | GLY182 | ASP52 | Hydrogen bond | 2.913 |
|  |  | LYS483 | GLU18 | Hydrogen bond | 2.857 |
|  |  | GLN104 | GLY76 | Hydrogen bond | 2.744 |
|  |  | GLU78 | LYS48 | Hydrogen bond | 2.566 |
|  |  | GLU75 | LYS48 | Hydrogen bond | 2.675 |
| SKP2–UbK48–p27 | 100 ns | VAL184 | GLU51 | Hydrogen bond | 2.910 |
|  |  | LYS483 | GLU18 | Hydrogen bond | 3.135 |
|  |  | SER183 | GLU51 | Hydrogen bond | 2.681 |
|  |  | GLN104 | GLY76 | Hydrogen bond | 2.864 |
|  |  | GLN77 | LYS48 | Hydrogen bond | 2.800 |
| SKP2–UbK48–p27 | 150 ns | SER183 | ASP58 | Hydrogen bond | 2.541 |
|  |  | PHE511 | HIS68 | Hydrogen bond | 2.894 |
|  |  | GLN104 | GLY76 | Hydrogen bond | 2.727 |
| SKP2–UbK48–p27 | 200 ns | ASP176 | LYS48 | Hydrogen bond | 2.548 |
|  |  | GLU172 | LYS48 | Hydrogen bond | 2.615 |
|  |  | GLU171 | ARG72 | Hydrogen bond | 2.784 |
|  |  | GLU171 | ARG72 | Hydrogen bond | 2.857 |
|  |  | LYS483 | GLU18 | Hydrogen bond | 2.727 |
|  |  | ARG152 | ARG74 | Hydrogen bond | 3.013 |
|  |  | GLN104 | GLY76 | Hydrogen bond | 2.813 |
|  |  | TRP60 | ASN60 | Hydrogen bond | 2.970 |
|  |  | GLN57 | GLN62 | Hydrogen bond | 2.682 |
|  |  | GLU53 | LYS63 | Hydrogen bond | 2.626 |

**Supplementary Table 6**: Time evolution of key intermolecular interactions observed during the MD trajectory (50, 100, 150, and 200 ns snapshots) for the SKP2–UbK48–p27 complex.

**
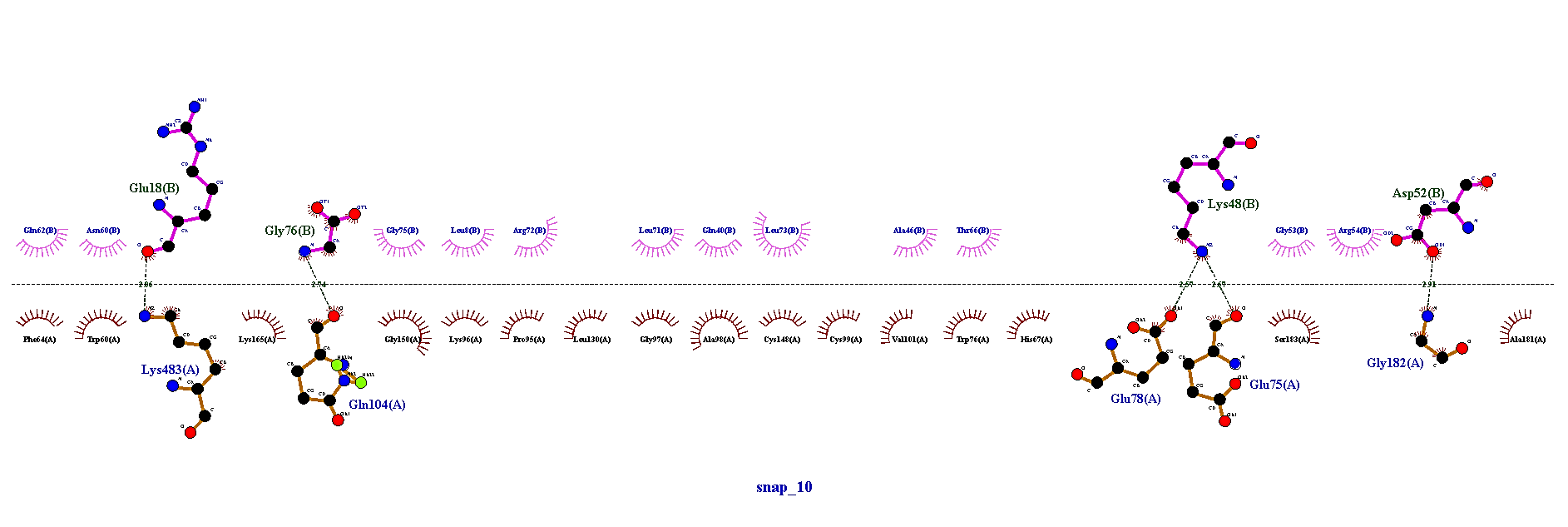
At 50 ns**

**
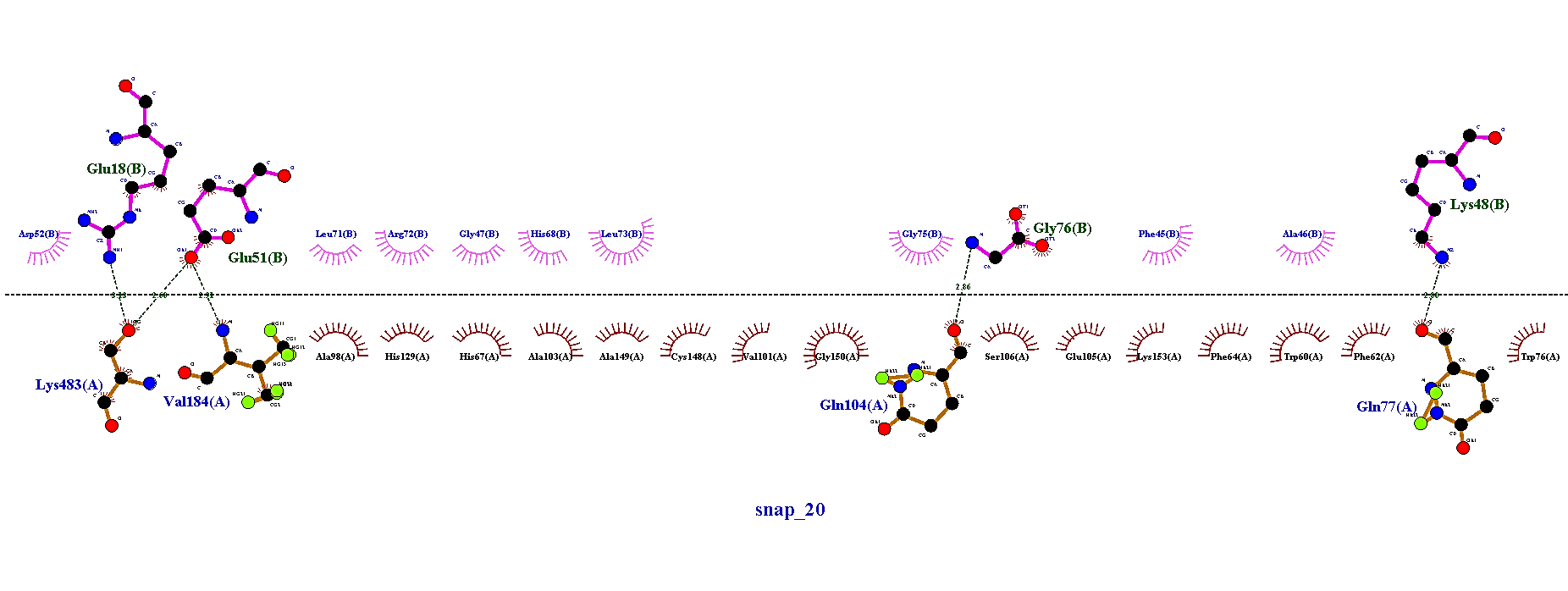
At 100 ns**

**
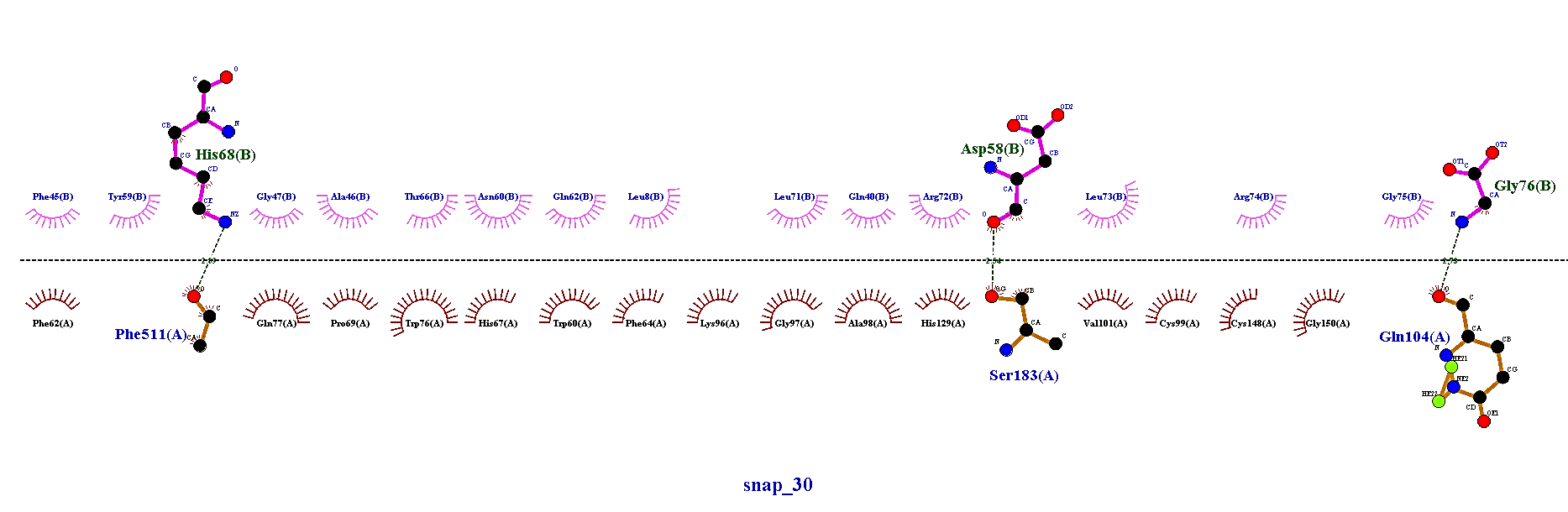
At 150 ns**

**
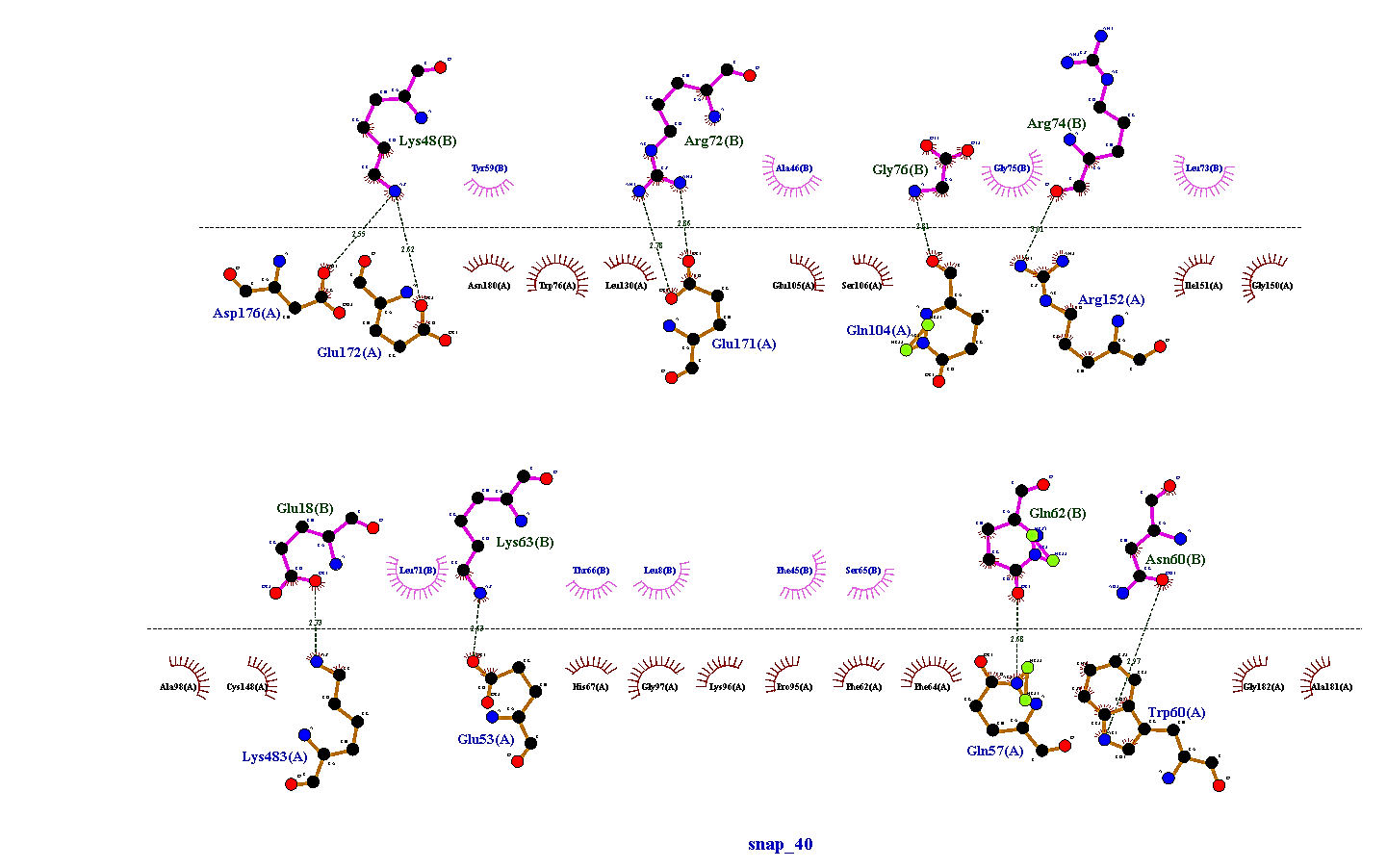
At 200 ns**

| **Complex** | **Snap-Duration** | **Residue 1 (Chain A)** | **Residue 2 (Chain B)** | **Interaction Type** | **Closest Distance (Å)** |
| --- | --- | --- | --- | --- | --- |
| SKP2-Akt1 | 50 ns | GLU194 | ARG25 | Hydrogen bond | 2.562 |
|  |  | GLN172 | GLU79 | Hydrogen bond | 2.851 |
|  |  | ASP171 | ARG25 | Hydrogen bond | 2.832 |
|  |  | ASP171 | LYS135 | Hydrogen bond | 2.759 |
|  |  | ASP150 | ARG57 | Hydrogen bond | 2.693 |
|  |  | LYS119 | ASP113 | Hydrogen bond | 2.823 |
|  |  | ARG69 | ASP176 | Hydrogen bond | 2.726 |
|  |  | ARG69 | ASP176 | Hydrogen bond | 2.540 |
|  |  | LYS68 | ASP41 | Hydrogen bond | 2.847 |
|  |  | ARG67 | PHE160 | Hydrogen bond | 3.257 |
|  |  | ARG67 | THR159 | Hydrogen bond | 2.957 |
|  |  | SER196 | LYS25 | Hydrogen bond | 2.970 |
|  |  | ASP11 | LYS251 | Hydrogen bond | 2.612 |
|  | 100 ns | GLU194 | ARG25 | Hydrogen bond | 2.747 |
|  |  | GLU194 | ARG25 | Hydrogen bond | 2.644 |
|  |  | GLU176 | ARG57 | Hydrogen bond | 2.847 |
|  |  | ALA175 | ARG57 | Hydrogen bond | 2.972 |
|  |  | ASP171 | LYS135 | Hydrogen bond | 2.664 |
|  |  | ARG69 | ASP176 | Hydrogen bond | 2.782 |
|  |  | ARG69 | THR156 | Hydrogen bond | 2.590 |
|  |  | LYS68 | ASP41 | Hydrogen bond | 2.616 |
|  |  | ARG67 | THR159 | Hydrogen bond | 2.975 |
|  |  | SER13 | LYS251 | Hydrogen bond | 2.959 |
|  |  | ASP11 | LYS251 | Hydrogen bond | 2.586 |
|  | 150 ns | ASP150 | ARG57 | Hydrogen bond | 3.984 |
|  |  | LYS68 | ASP41 | Hydrogen bond | 2.829 |
|  |  | GLU194 | ARG25 | Hydrogen bond | 2.779 |
|  |  | GLN172 | GLU79 | Hydrogen bond | 3.040 |
|  |  | ASP171 | ARG25 | Hydrogen bond | 2.803 |
|  |  | ASP171 | LYS135 | Hydrogen bond | 2.926 |
|  |  | LYS145 | GLU107 | Hydrogen bond | 2.688 |
|  |  | LYS119 | ASP113 | Hydrogen bond | 2.617 |
|  |  | GLU116 | GLN255 | Hydrogen bond | 3.101 |
|  |  | GLU116 | LYS251 | Hydrogen bond | 2.645 |
|  |  | ARG69 | ASP176 | Hydrogen bond | 2.600 |
|  |  | ARG69 | THR156 | Hydrogen bond | 2.868 |
|  |  | ARG67 | THR159 | Hydrogen bond | 2.825 |
|  |  | GLU42 | LYS152 | Hydrogen bond | 2.766 |
|  | 200 ns | LYS119 | ASP113 | Hydrogen bond | 2.881 |
|  |  | GLU116 | LYS251 | Hydrogen bond | 2.651 |
|  |  | GLU194 | ARG25 | Hydrogen bond | 2.518 |
|  |  | GLU176 | ARG57 | Hydrogen bond | 2.598 |
|  |  | GLU176 | ARG57 | Hydrogen bond | 2.868 |
|  |  | GLN172 | GLU79 | Hydrogen bond | 2.563 |
|  |  | ASP171 | LYS135 | Hydrogen bond | 2.631 |
|  |  | ASP150 | ARG57 | Hydrogen bond | 2.767 |
|  |  | ARG69 | ASP176 | Hydrogen bond | 2.628 |
|  |  | ARG69 | THR156 | Hydrogen bond | 2.846 |
|  |  | LYS68 | ASP41 | Hydrogen bond | 2.581 |
|  |  | ARG67 | THR159 | Hydrogen bond | 2.795 |
|  |  | GLU42 | LYS152 | Hydrogen bond | 2.488 |

**Supplementary Table 7**: Time evolution of key intermolecular interactions observed during the MD trajectory (50, 100, 150, and 200 ns snapshots) for the SKP2–Akt1 complex.

**
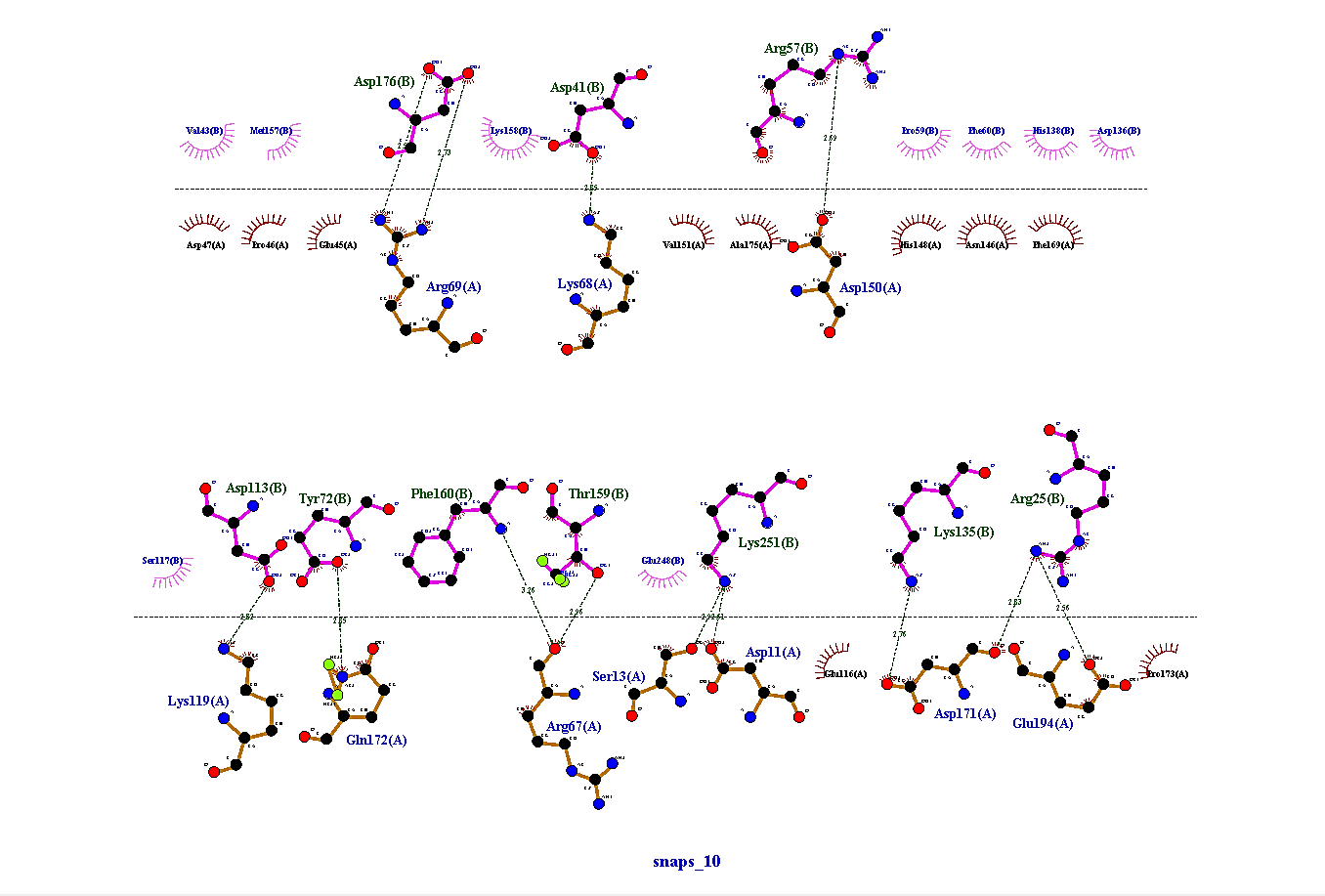
At 50 ns**

**
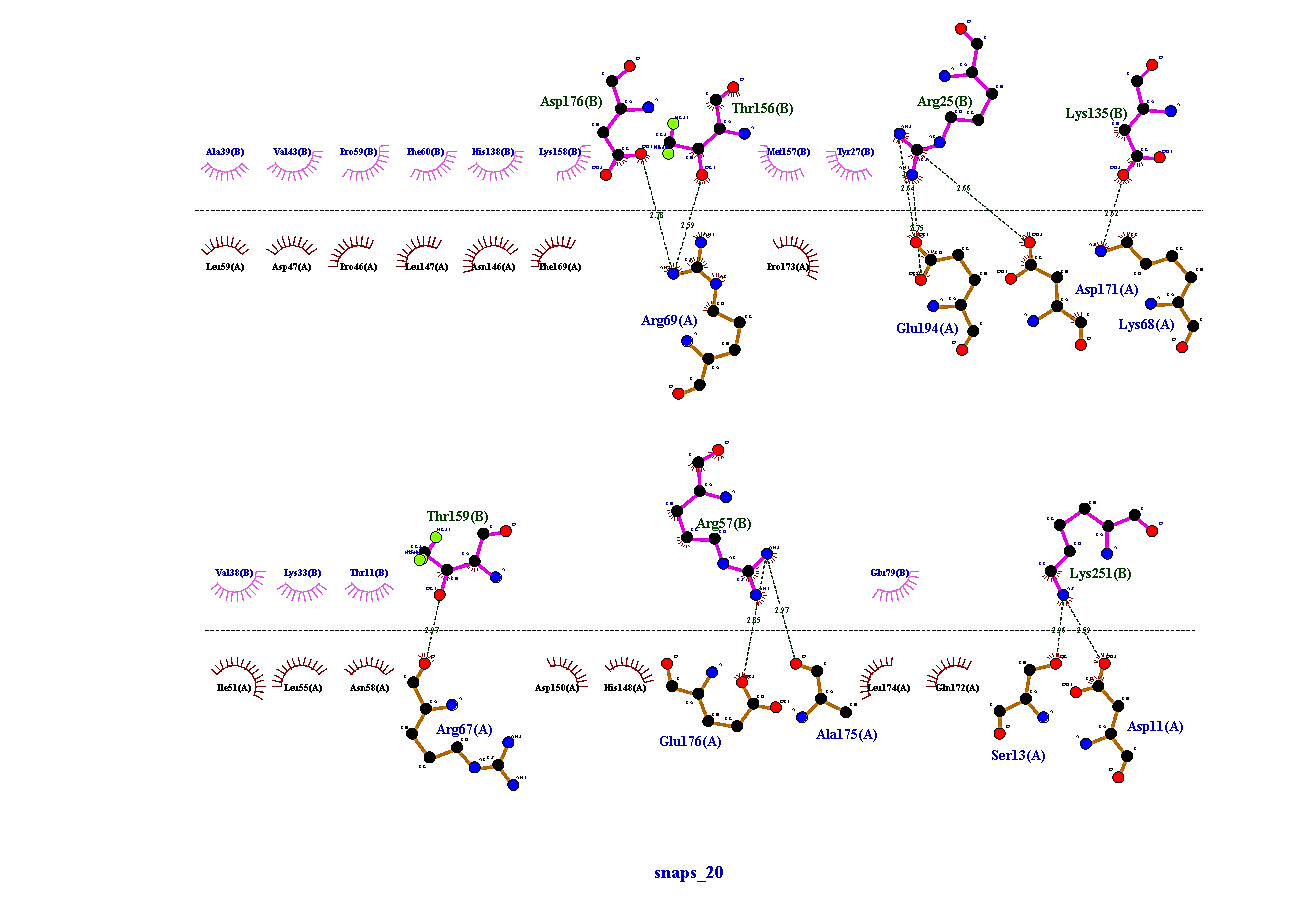
At 100 ns**

**At 150 ns**

**
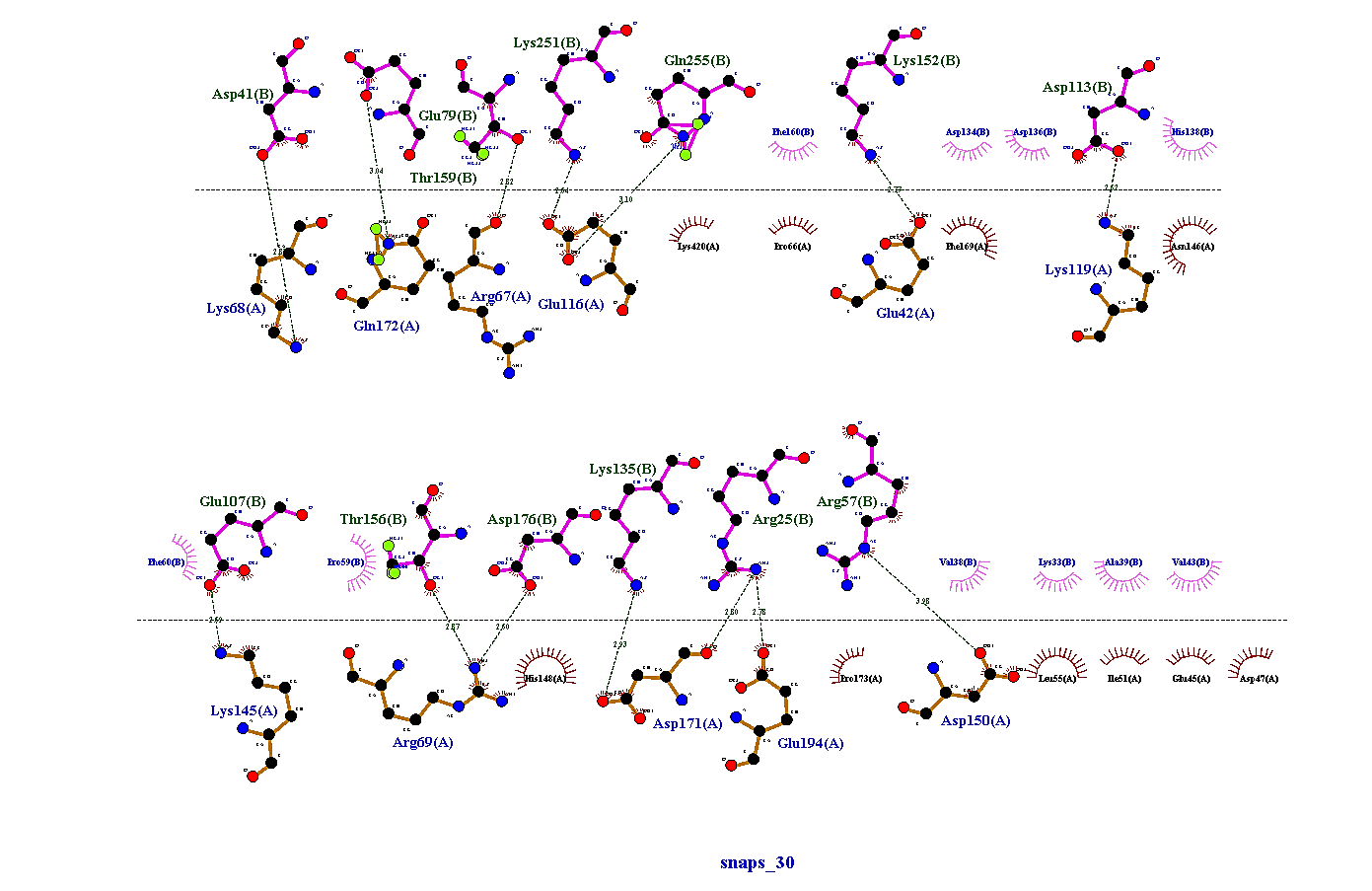
**

**
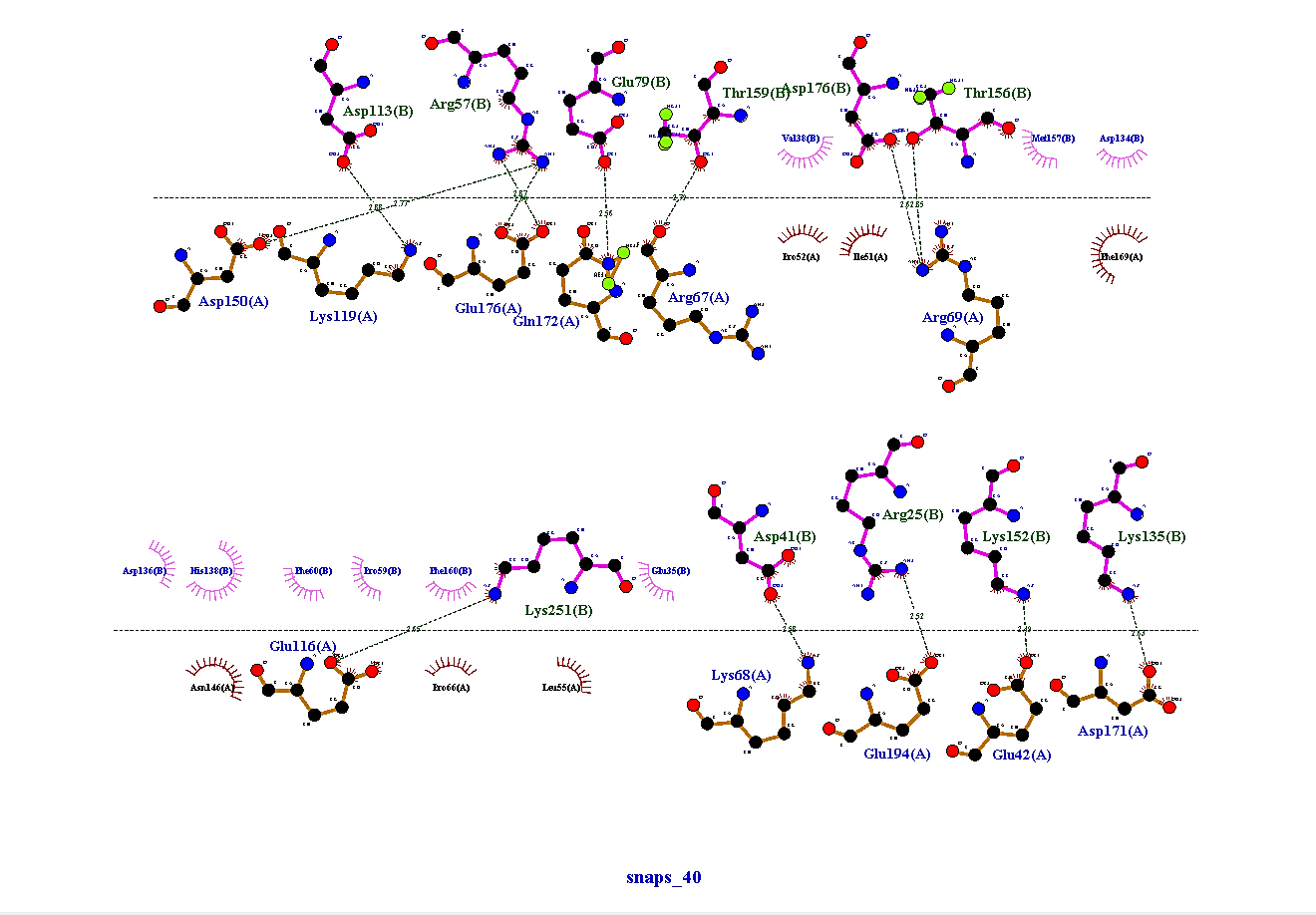
At 200 ns**

| **Complex** | **Snap-Duration** | **Residue 1 (Chain A)** | **Residue 2 (Chain B)** | **Interaction Type** | **Closest Distance (Å)** |
| --- | --- | --- | --- | --- | --- |
| SKP2-UbK63-Akt1 | 50 ns | LYS4 | GLU323 | Hydrogen bond | 3.414 |
|  |  | ARG182 | ASP136 | Hydrogen bond | 2.663 |
|  |  | HIS177 | GLU93 | Hydrogen bond | 2.790 |
|  |  | ARG154 | ASP99 | Hydrogen bond | 2.751 |
|  |  | GLU134 | LYS135 | Hydrogen bond | 2.664 |
|  |  | ARG129 | GLU107 | Hydrogen bond | 2.683 |
|  |  | ARG126 | HIS256 | Hydrogen bond | 2.665 |
|  |  | ASP98 | ARG333 | Hydrogen bond | 2.847 |
|  |  | ARG84 | ASP153 | Hydrogen bond | 2.909 |
|  |  | ARG84 | ASP153 | Hydrogen bond | 2.775 |
|  |  | ARG69 | GLU252 | Hydrogen bond | 2.544 |
|  |  | ARG67 | GLU226 | Hydrogen bond | 2.687 |
|  |  | ARG67 | GLU226 | Hydrogen bond | 2.611 |
|  | 100 ns | ARG182 | ASP136 | Hydrogen bond | 2.884 |
|  |  | ARG182 | ASP136 | Hydrogen bond | 2.658 |
|  |  | HIS177 | GLU93 | Hydrogen bond | 2.733 |
|  |  | ARG129 | ASP136 | Hydrogen bond | 2.737 |
|  |  | ARG126 | HIS256 | Hydrogen bond | 2.765 |
|  |  | ASP102 | GLN261 | Hydrogen bond | 2.738 |
|  |  | SER99 | LEU330 | Hydrogen bond | 2.798 |
|  |  | ASP98 | ARG333 | Hydrogen bond | 2.670 |
|  |  | ASP98 | ARG333 | Hydrogen bond | 2.626 |
|  |  | GLU90 | ARG333 | Hydrogen bond | 2.607 |
|  |  | ARG89 | GLY334 | Hydrogen bond | 3.254 |
|  |  | ARG84 | ASP153 | Hydrogen bond | 2.807 |
|  |  | ARG69 | GLU252 | Hydrogen bond | 2.837 |
|  |  | ARG67 | GLU226 | Hydrogen bond | 2.653 |
|  |  | ARG67 | GLU226 | Hydrogen bond | 2.852 |
|  | 150 ns | ARG182 | ASP136 | Hydrogen bond | 2.673 |
|  |  | ARG182 | ASP136 | Hydrogen bond | 2.690 |
|  |  | HIS177 | GLU93 | Hydrogen bond | 2.701 |
|  |  | ARG129 | GLU107 | Hydrogen bond | 2.627 |
|  |  | ARG129 | GLU107 | Hydrogen bond | 2.698 |
|  |  | ASP98 | ARG333 | Hydrogen bond | 2.749 |
|  |  | ASP98 | ARG333 | Hydrogen bond | 2.633 |
|  |  | ARG89 | ARG333 | Hydrogen bond | 2.719 |
|  |  | ARG84 | ASP153 | Hydrogen bond | 2.616 |
|  |  | ARG84 | ASP153 | Hydrogen bond | 3.257 |
|  |  | ARG69 | GLU252 | Hydrogen bond | 2.636 |
|  |  | ARG67 | GLU252 | Hydrogen bond | 3.275 |
|  |  | ARG67 | GLU226 | Hydrogen bond | 2.808 |
|  |  | ARG67 | GLU226 | Hydrogen bond | 2.501 |
|  |  | ARG67 | GLU252 | Hydrogen bond | 2.863 |
|  | 200 ns | ARG182 | ASP136 | Hydrogen bond | 2.845 |
|  |  | ARG129 | GLU107 | Hydrogen bond | 2.583 |
|  |  | ARG129 | GLU107 | Hydrogen bond | 2.720 |
|  |  | ASP102 | GLN261 | Hydrogen bond | 2.864 |
|  |  | SER99 | LEU330 | Hydrogen bond | 3.052 |
|  |  | ASP98 | ARG333 | Hydrogen bond | 2.729 |
|  |  | ASP98 | ARG333 | Hydrogen bond | 2.757 |
|  |  | ARG89 | ARG333 | Hydrogen bond | 2.772 |
|  |  | ARG84 | ASP153 | Hydrogen bond | 2.693 |
|  |  | ARG69 | GLU252 | Hydrogen bond | 2.551 |
|  |  | ARG67 | GLU226 | Hydrogen bond | 2.720 |
|  |  | ARG67 | GLU226 | Hydrogen bond | 2.957 |
|  |  | ARG67 | GLU252 | Hydrogen bond | 2.760 |

**Supplementary Table 8**: Time evolution of key intermolecular interactions observed during the MD trajectory (50, 100, 150, and 200 ns snapshots) for the SKP2–UbK63–Akt1 complex.

**
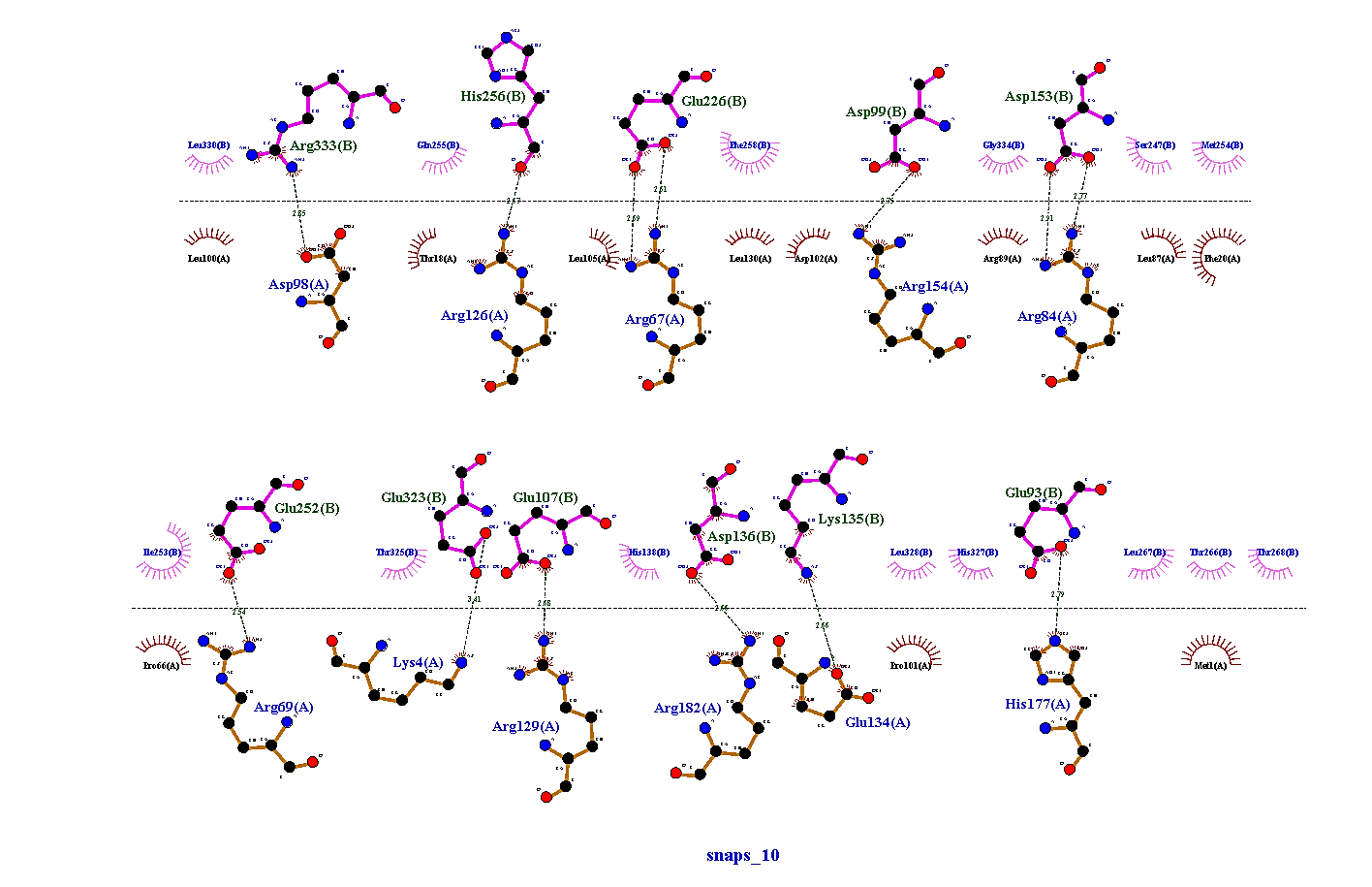
At 50 ns**

**
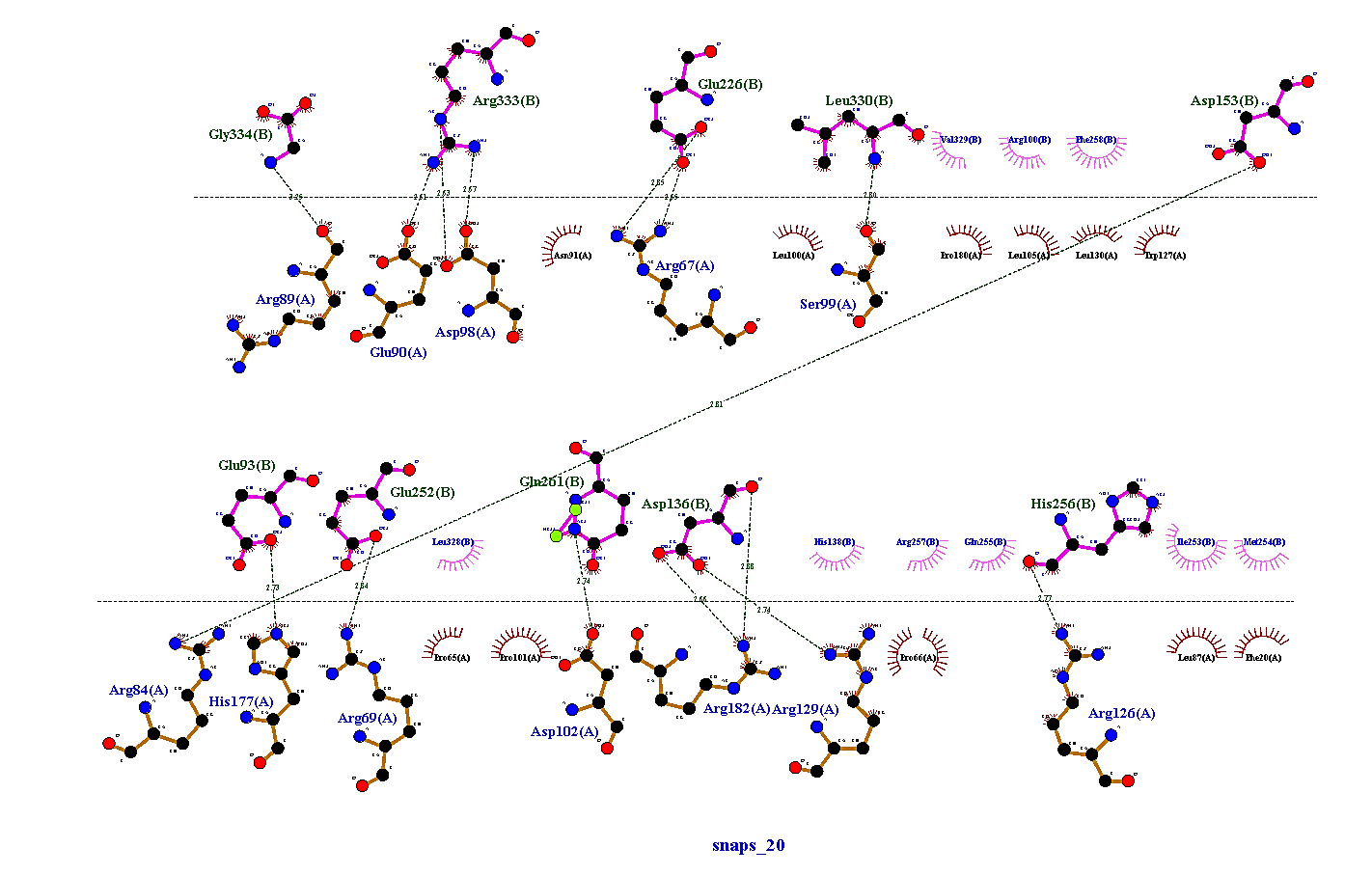
At 100 ns**

**
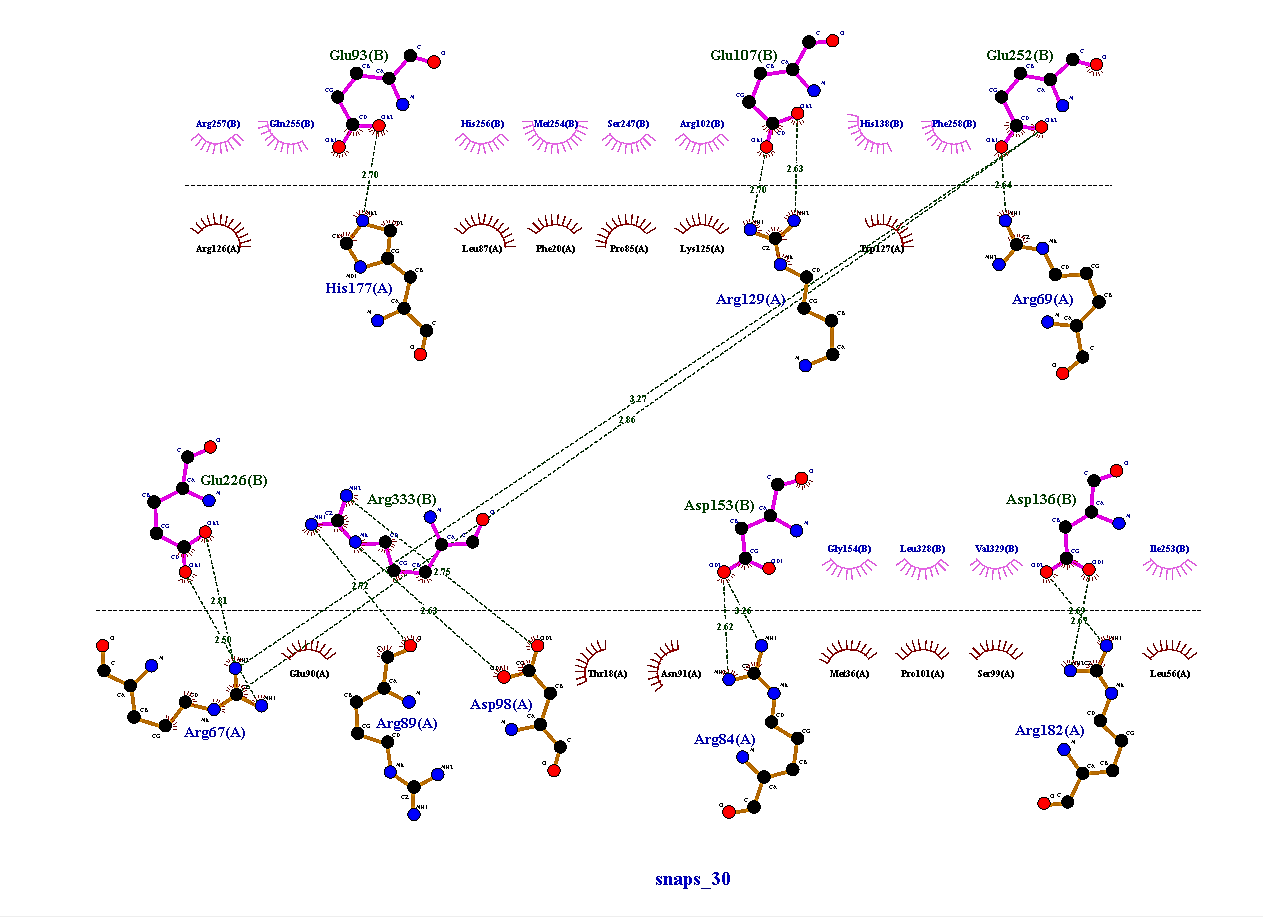
At 150 ns**

**
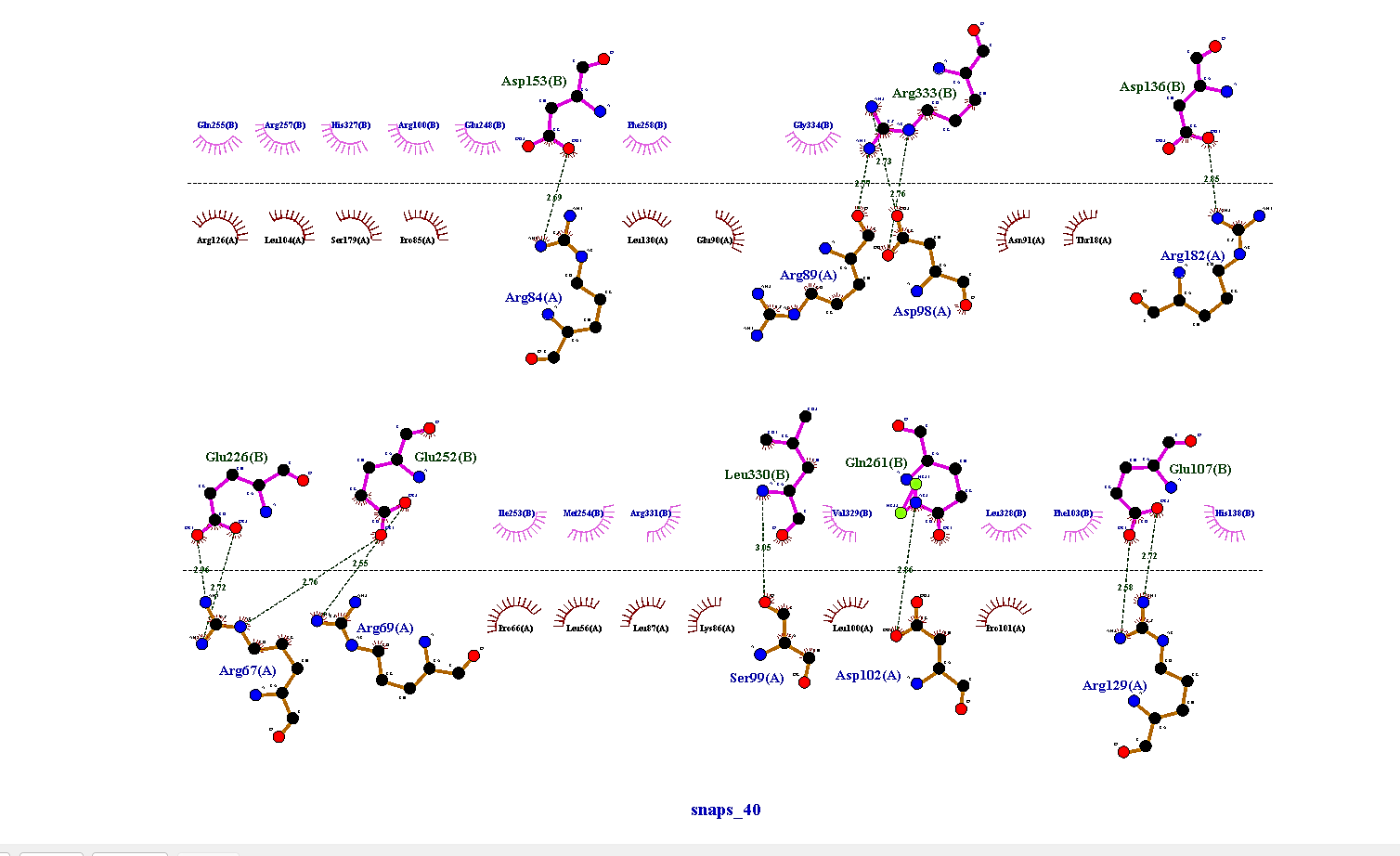
At 200 ns**
